# Supplementary figures and images for: A shared transcriptional code orchestrates temporal patterning of the central nervous system
Source: PLoS Biol. 2021 Nov 12;19(11):e3001450. doi: 10.1371/journal.pbio.3001450 (PMC8612522; doi:10.1371/journal.pbio.3001450)

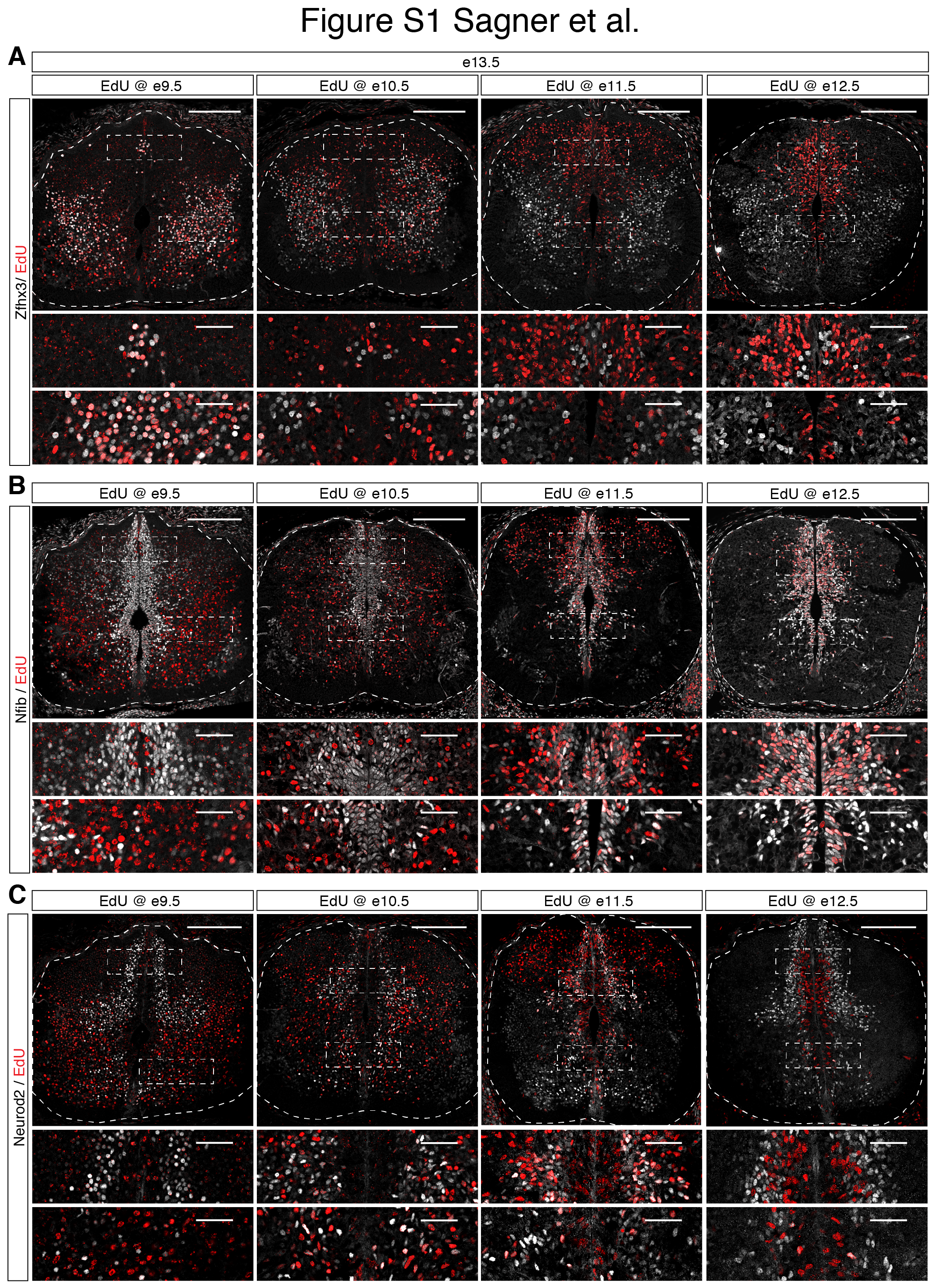

Supplement: S1 Fig — (A-C). Colocalization between Zfhx3 (A), Nfib (B), Neurod2 (C), and EdU administered at e9.5, e10.5, e11.5, or e12.5 (from left to right) in e13.5 spinal cord sections. Scale bars in overview pictures = 200 μm, insets = 50 μm. TF, transcription factor. (PNG) [file pbio.3001450.s001.png]

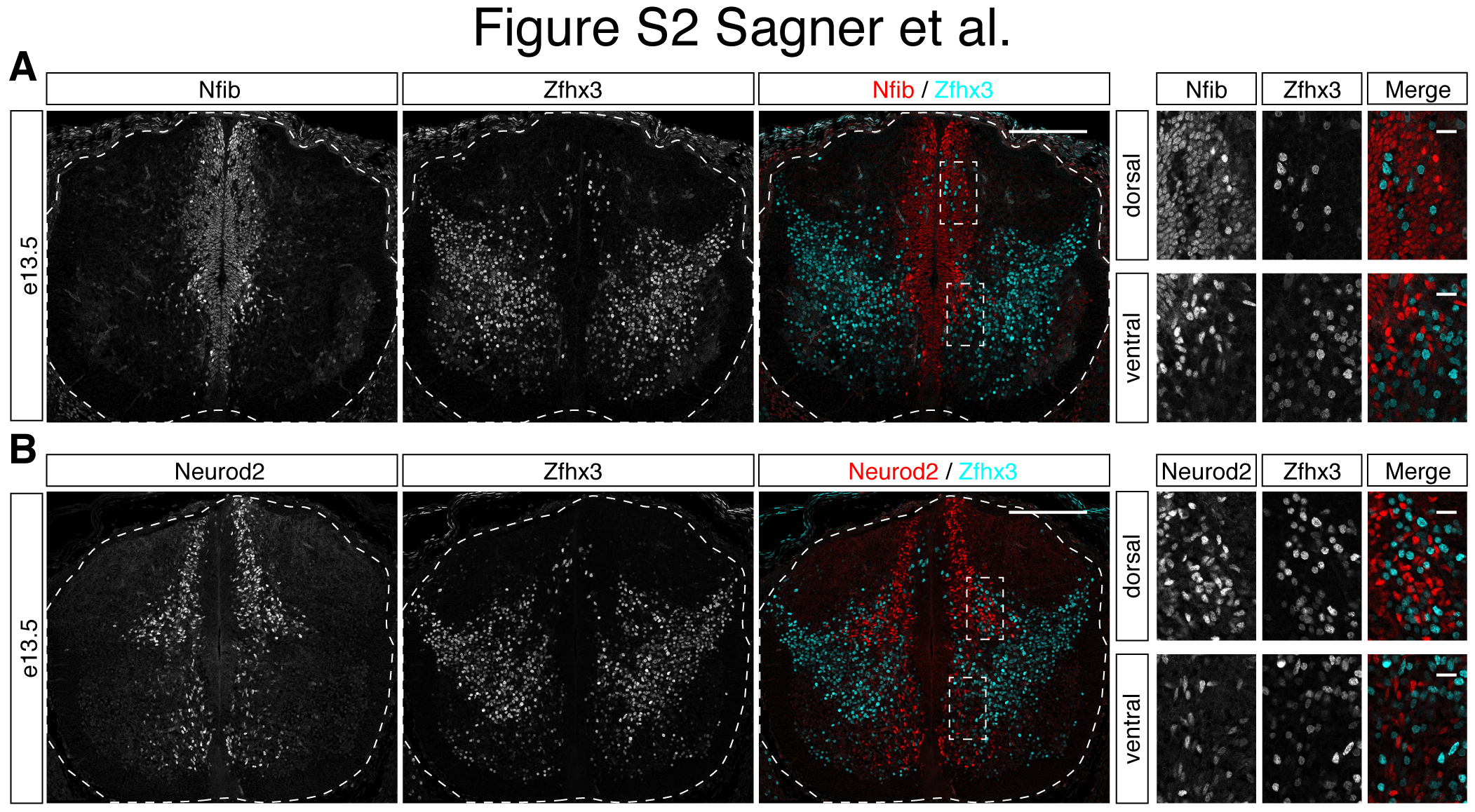

Supplement: S2 Fig — (A, B) Zfhx3 and Nfib (A) or Zfhx3 and Neurod2 (B) are expressed in mutually exclusive populations of neurons in the spinal cord. Scale bars in overview pictures = 200 μm, insets = 20 μm. TF, transcription factor. (PNG) [file pbio.3001450.s002.png]

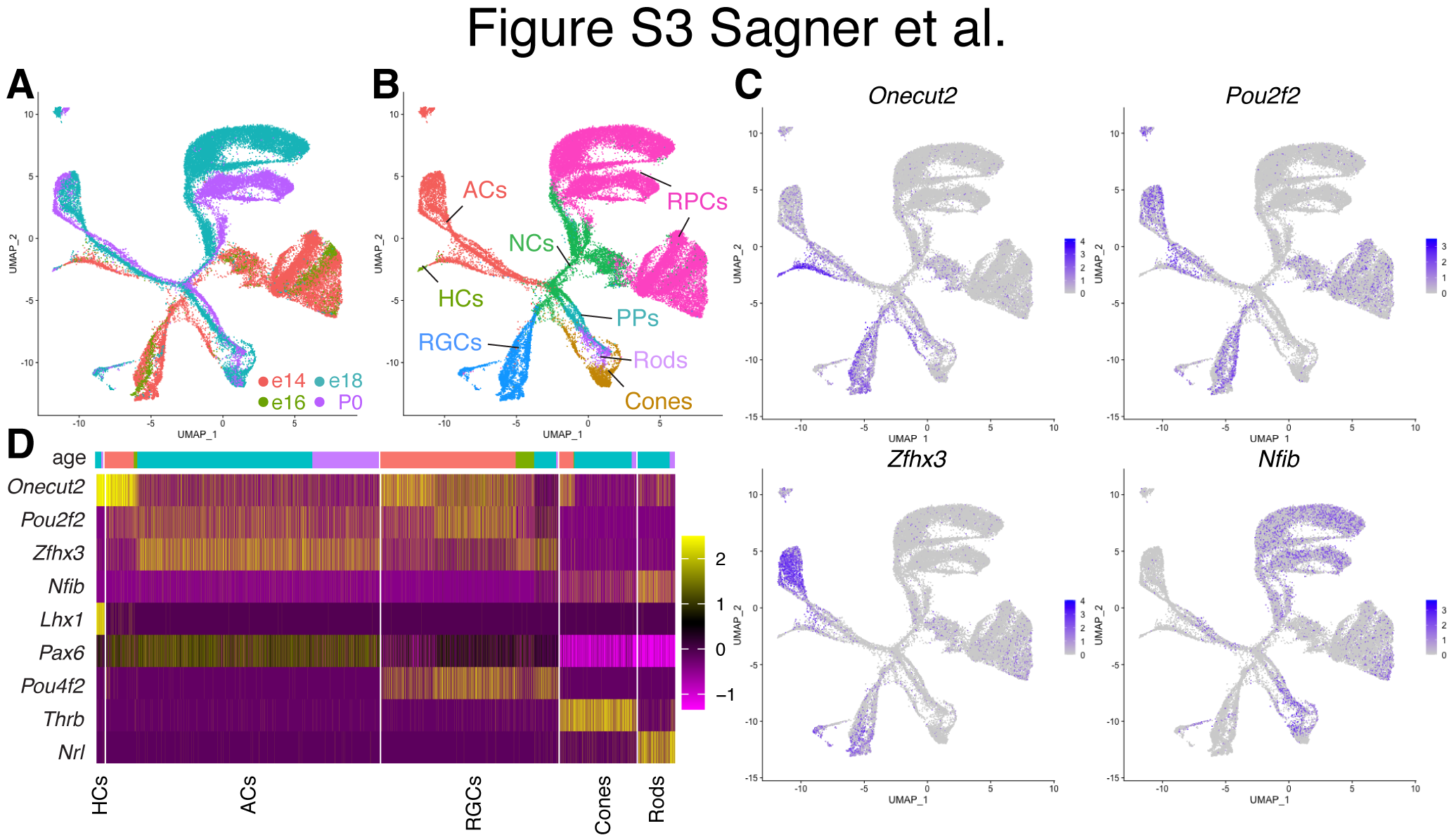

Supplement: S3 Fig — (A) UMAP representation of scRNAseq data from the developing mouse retina [43] color coded by developmental stage. (B) Same UMAP representation as (A) color coded for cell identity. (C) Expression levels of Onecut2, Pou2f2, Zfhx3, and Nfib in individual cells. (D) Heatmap indicating expression levels of the temporal TFs (Onecut2, Pou2f2, Zfhx3, and Nfib) and known marker genes (Lhx1, Pax6, Pou4f2, Thrb, and Nrl) in different types of retinal neurons stratified by developmental age. AC, amacrine cell; HC, horizontal cell; RGC, retinal ganglion cell; RPC, retinal progenitor cell; NCs, neurogenic cell; PP, photoreceptor precursor; scRNAseq, single-cell RNA sequencing; TF, transcription factor; UMAP, Uniform Manifold Approximation and Projection. (PNG) [file pbio.3001450.s003.png]

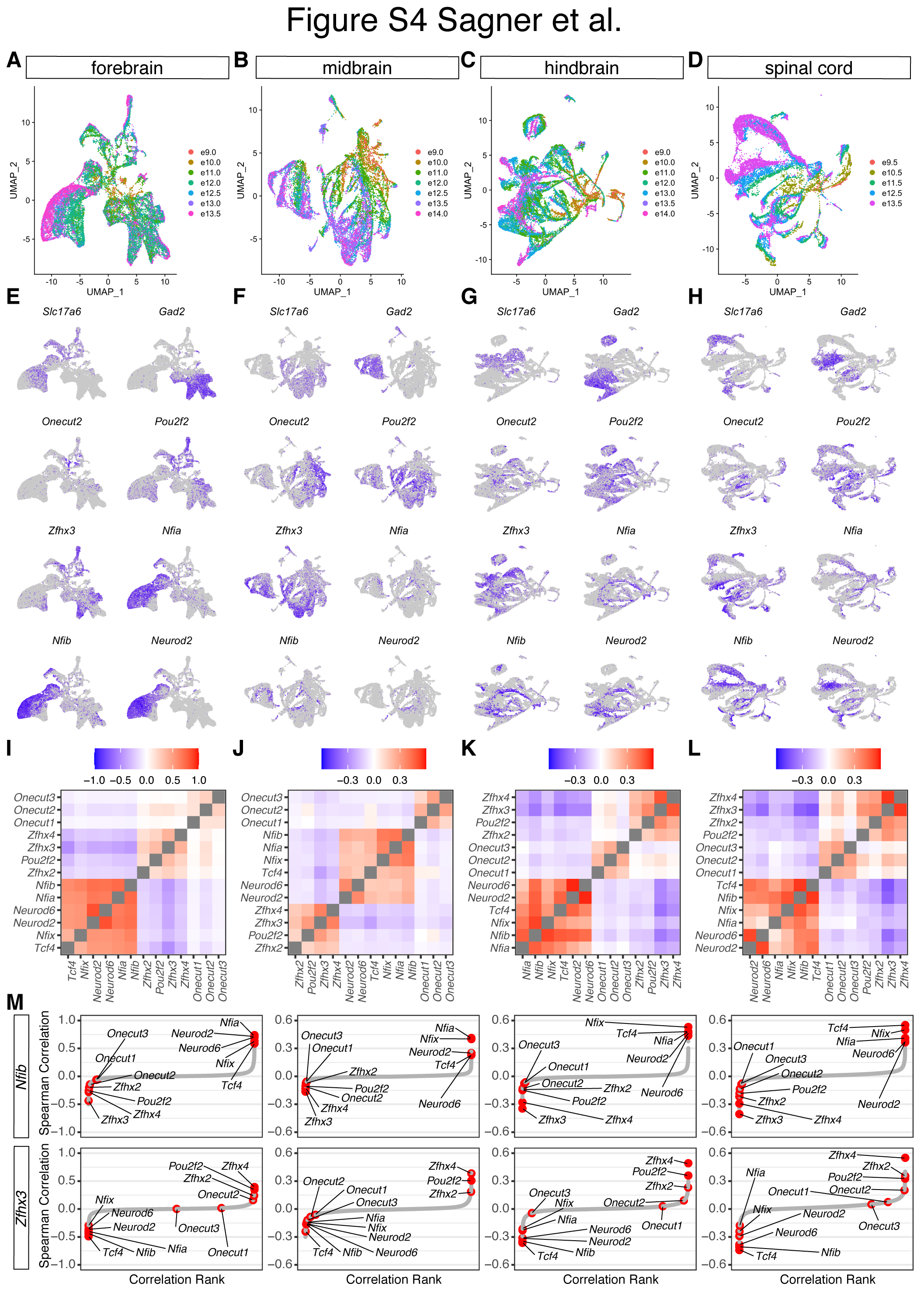

Supplement: S4 Fig — (A-D). UMAP representation of scRNAseq data from (A) forebrain, (B) midbrain, (C) hindbrain [9], and (D) spinal cord [33] color coded by developmental stage. (E-H) Expression levels of Slc17a6, Gad2, Onecut2, Pou2f2, Zfhx3, Nfia, Nfib, and Neurod2 in individual cells. (I-L) Heatmaps indicating Spearman correlation between temporal TF expression in the different regions of the nervous system. (M) Spearman correlation rank plots for Nfib (top row) and Zfhx3 (bottom row) in the scRNAseq data from forebrain, midbrain, hindbrain, and spinal cord (left to right). Data points corresponding to temporal TFs are highlighted in red. scRNAseq, single-cell RNA sequencing; TF, transcription factor; UMAP, Uniform Manifold Approximation and Projection. (PNG) [file pbio.3001450.s004.png]

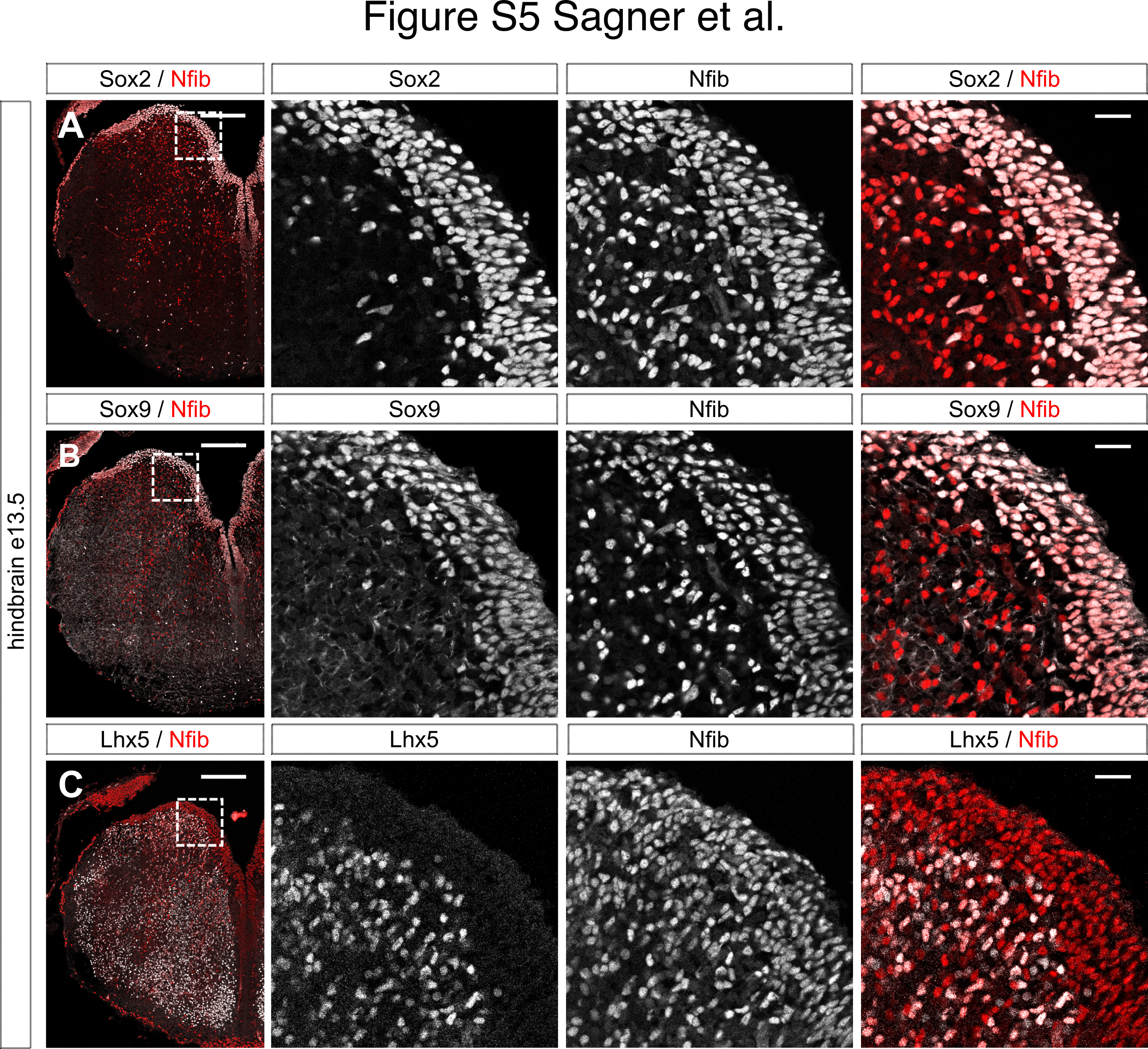

Supplement: S5 Fig — (A-C) e13.5 hindbrain sections stained for Nfib and the progenitor marker Sox2 (A), the glial progenitor marker Sox9 (B), and the neuronal marker Lhx5 (C). Scale bars in overview pictures = 200 μm, insets = 25 μm. (PNG) [file pbio.3001450.s005.png]

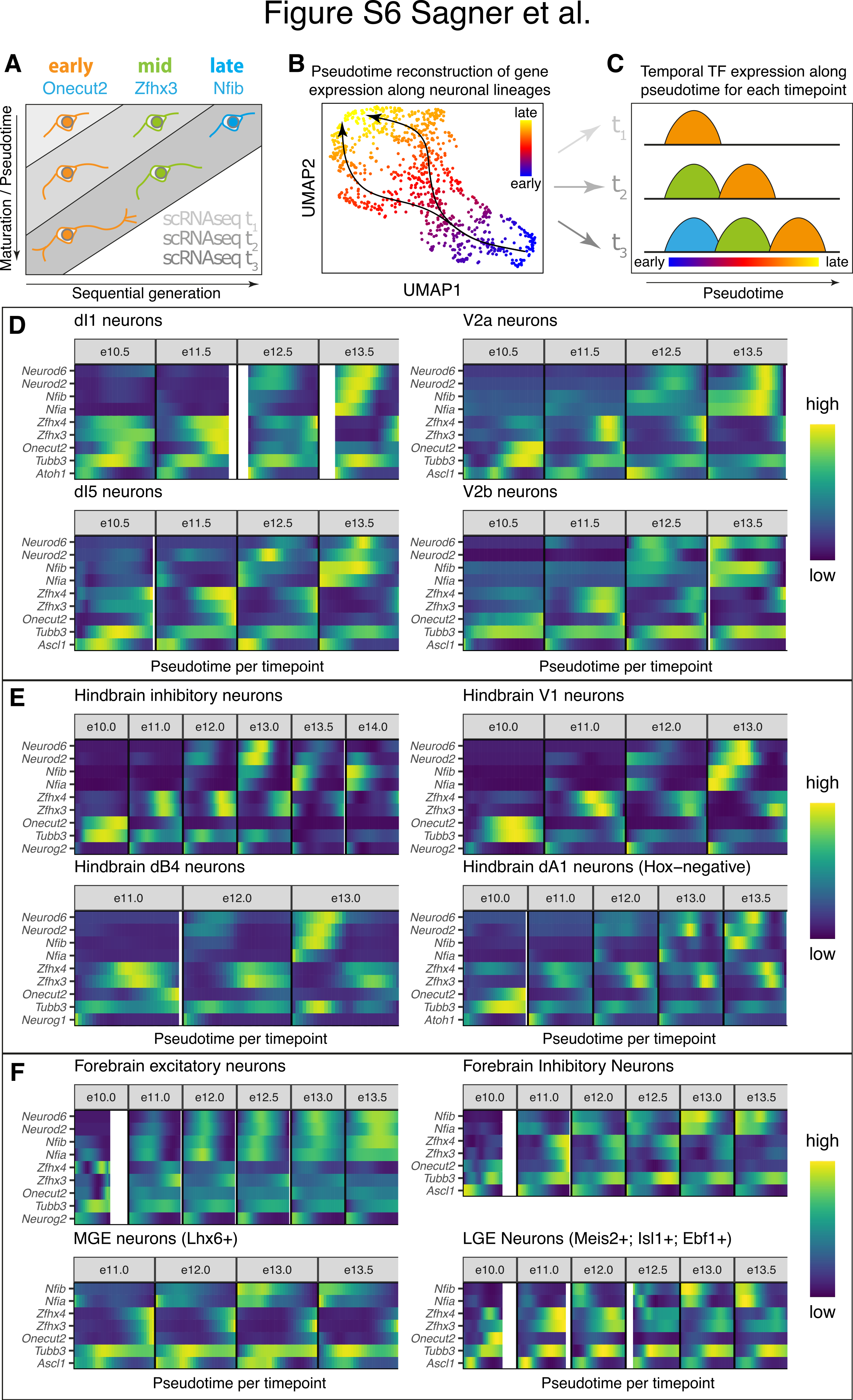

Supplement: S6 Fig — (A) Sequential generation of neurons expressing temporal TFs should result in the capture of neurons at different stages of their differentiation trajectory in scRNAseq time course data. (B) Pseudotime reconstruction of gene expression dynamics along a neuronal differentiation trajectory. Dark blue corresponds to early cells, yellow to late cells along the differentiation trajectory. Arrows indicate predicted pseudotime trajectories. (C) Temporal TFs should be sequentially expressed in pseudotime. (D) Pseudotime reconstruction of gene expression for different neuronal lineages along the dorsal–ventral axis of the spinal cord reveals sequential expression of temporal TFs. (E, F) Similar gene expression dynamics are observed when pseudotemporal gene expression is reconstructed for neuronal lineages in the hindbrain (E) and forebrain (F). LGE, lateral ganglionic eminence; MGE, medial ganglionic eminence; scRNAseq, single-cell RNA sequencing; TF, transcription factor. (PNG) [file pbio.3001450.s006.png]

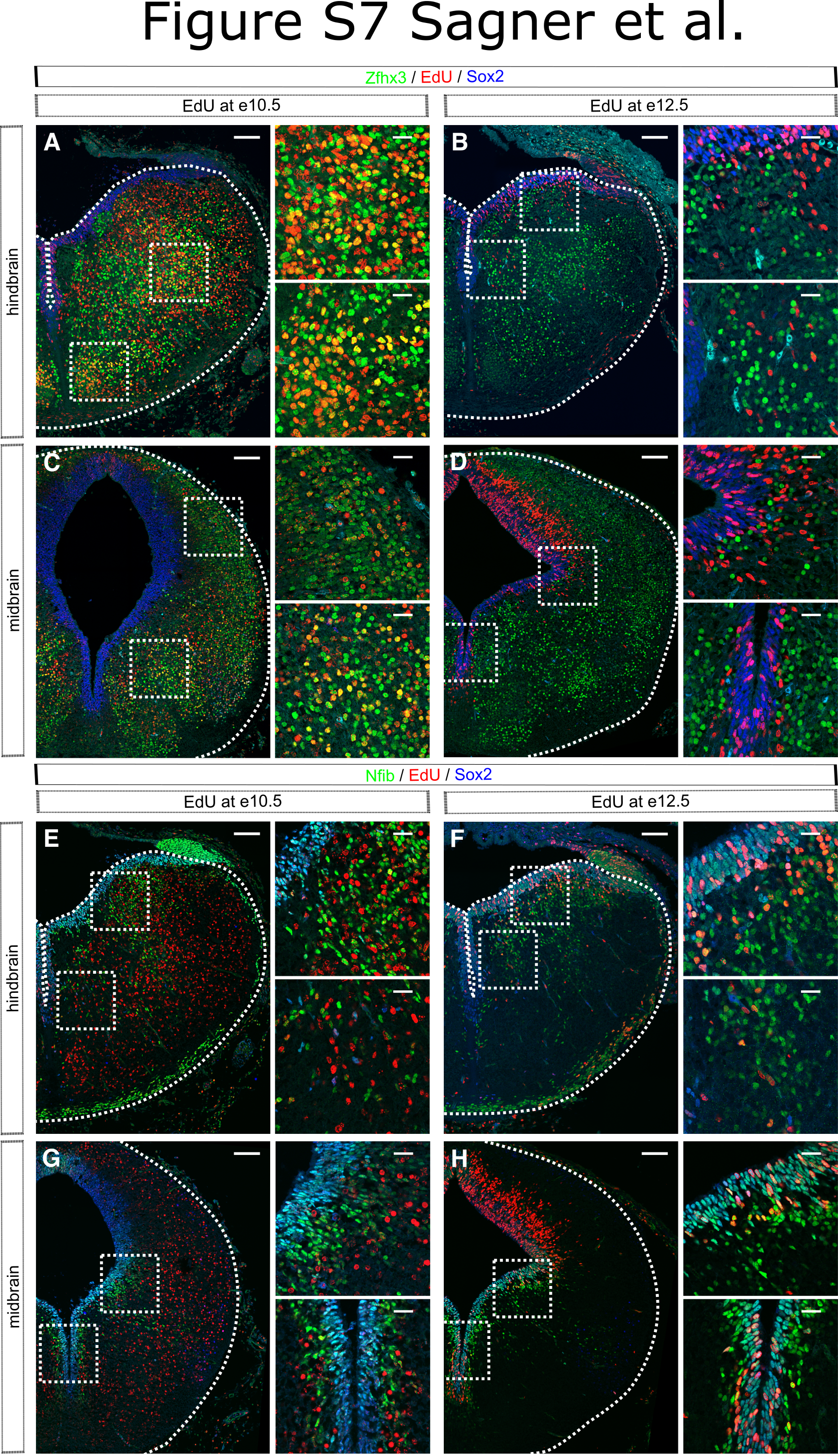

Supplement: S7 Fig — (A-D) e13.5 hindbrain (A, B) and midbrain (C, D) sections stained for Zfhx3 (green), EdU (red), and Sox2 (blue). EdU was administered at e10.5 (A, C) or e12.5 (B, D). (E-H) e13.5 hindbrain (E, F) and midbrain (G, H) sections stained for Nfib (green), EdU (red), and Sox2 (blue). EdU was administered at e10.5 (E, G) or e12.5 (F, H). Scale bars in overview pictures = 100 μm, insets = 25 μm. (PNG) [file pbio.3001450.s007.png]

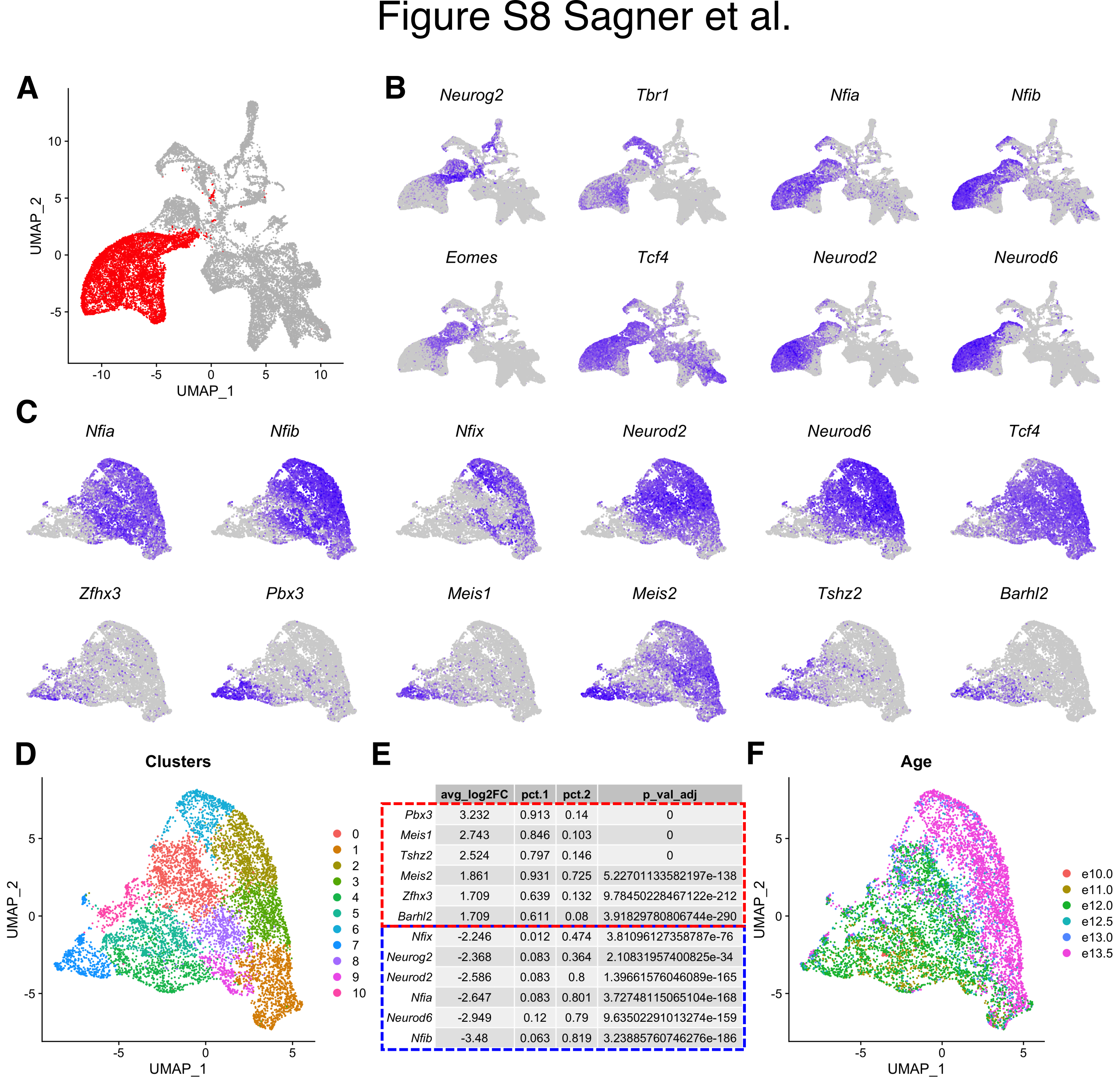

Supplement: S8 Fig — (A) UMAP plots of all e10–e13.5 forebrain neurons in the dataset from La Manno and colleagues. Cortical excitatory neurons are colored in red. (B) UMAP plots indicating the expression of marker genes characteristic for cortical excitatory neurons. (C) UMAP plots showing widespread expression of late temporal TFs in forebrain excitatory neurons (top row) and expression of marker genes for cluster 7 neurons (see D) (bottom row). (D) Identification of different clusters of cortical excitatory neurons. Cluster 7 corresponds to the Zfhx3-positive population of neurons (see also C). (E) Differential gene expression analysis comparing cluster 7 cells to the rest of the identified cortical excitatory neurons. The top 6 TFs up-regulated in this cluster are indicated by the red box, the top 6 down-regulated TFs by the blue box. (F) UMAP plot of cortical excitatory neurons (red cells in A) color coded for the developmental stage from which these cells were obtained. TF, transcription factor; UMAP, Uniform Manifold Approximation and Projection. (PNG) [file pbio.3001450.s008.png]

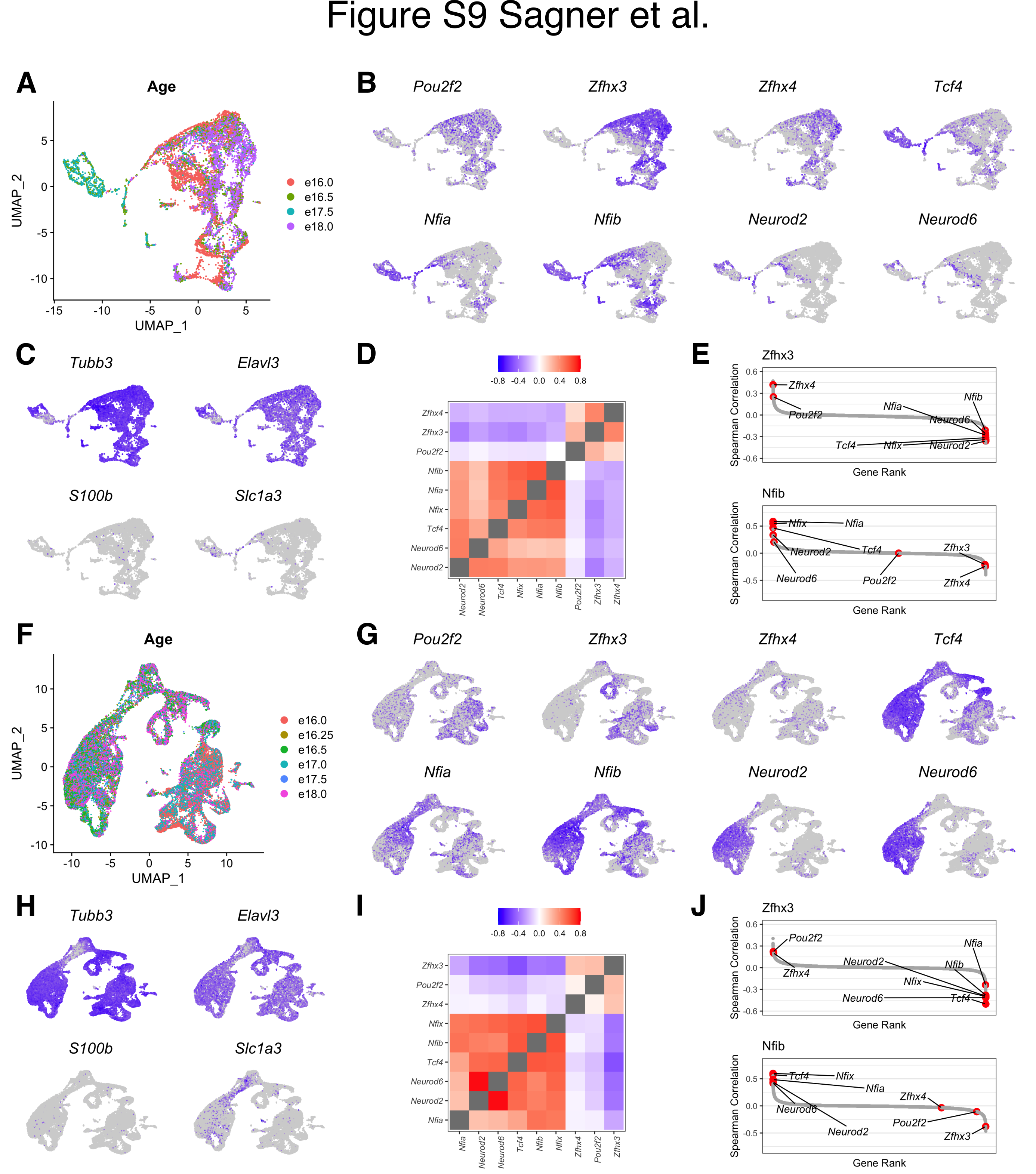

Supplement: S9 Fig — (A, F) UMAP plots from late midbrain (A) and forebrain (F) neurons (e16–e18) color coded for the developmental stage from which these cells were obtained. (B, G) UMAP plots from late midbrain (B) and forebrain (G) neurons showing expression of the indicated markers. Expression of intermediate and late temporal markers (especially Zfhx3/4 and Nfia/b) stay highly anticorrelated in neurons. (C, H) Nfia and Nfib-positive cells express the neuronal markers Tubb3 and Elavl3 but not the glial markers S100b and Slc1a3. (D, I) Heatmaps indicating Spearman correlation between intermediate and late temporal TFs in the late embryonic midbrain (D) and forebrain (I). (E, J) Correlation rank plots for Zfhx3 and Nfib indicate that these markers stay highly anticorrelated in the late embryonic midbrain (E) and forebrain (J). scRNAseq, single-cell RNA sequencing; TF, transcription factor; UMAP, Uniform Manifold Approximation and Projection. (PNG) [file pbio.3001450.s009.png]

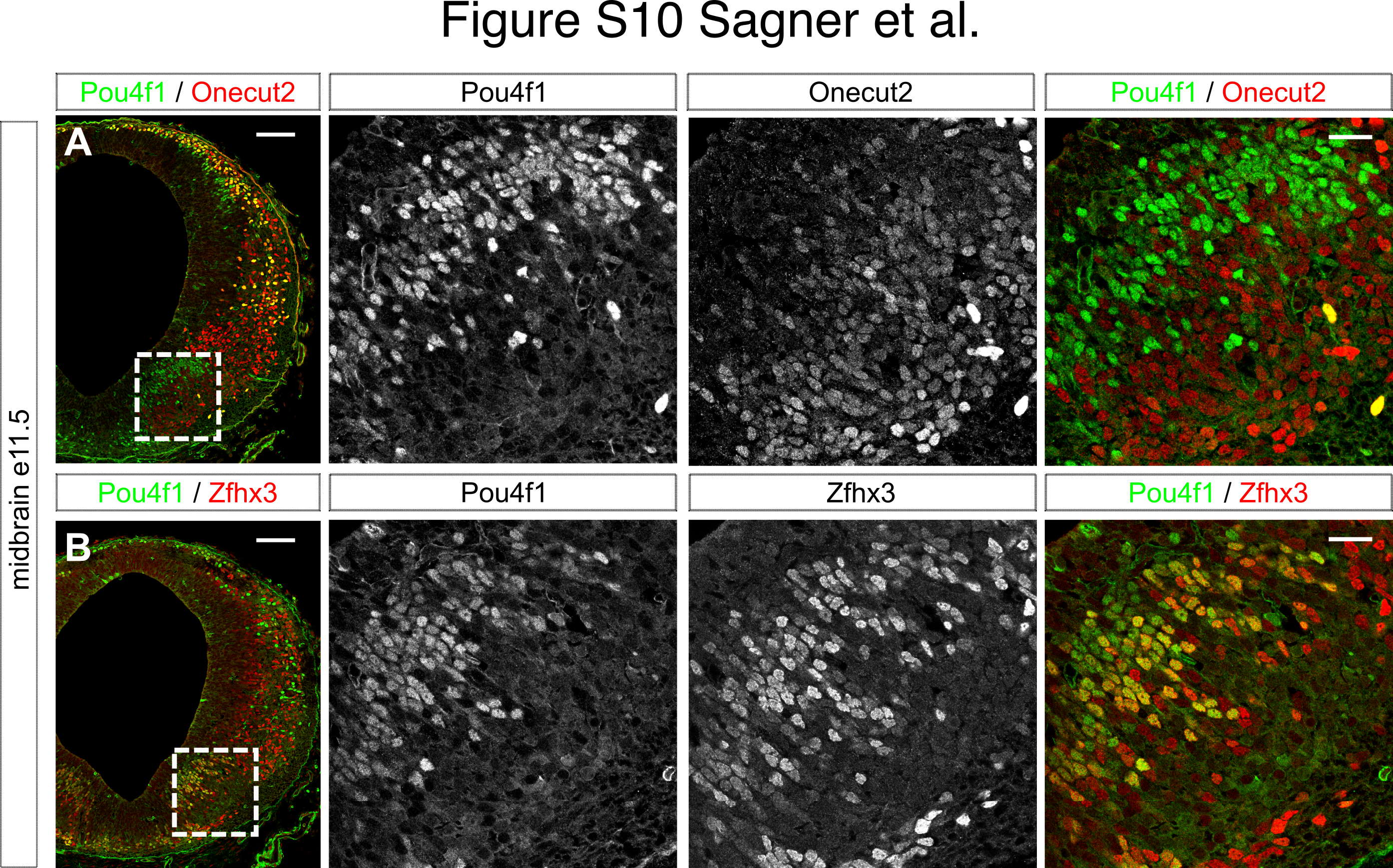

Supplement: S10 Fig — (A, B) e11.5 midbrain cryosection stained for (A) Onecut2 (red) and Pou4f1 (green) or (B) Zfhx3 (red) and Pou4f1 (green). Note the Onecut2 expression is mutually exclusive with Pou4f1 expression (A) while most Pou4f1 neurons express Zfhx3 (B). Scale bars in overview pictures = 200 μm, insets = 25 μm. (PNG) [file pbio.3001450.s010.png]

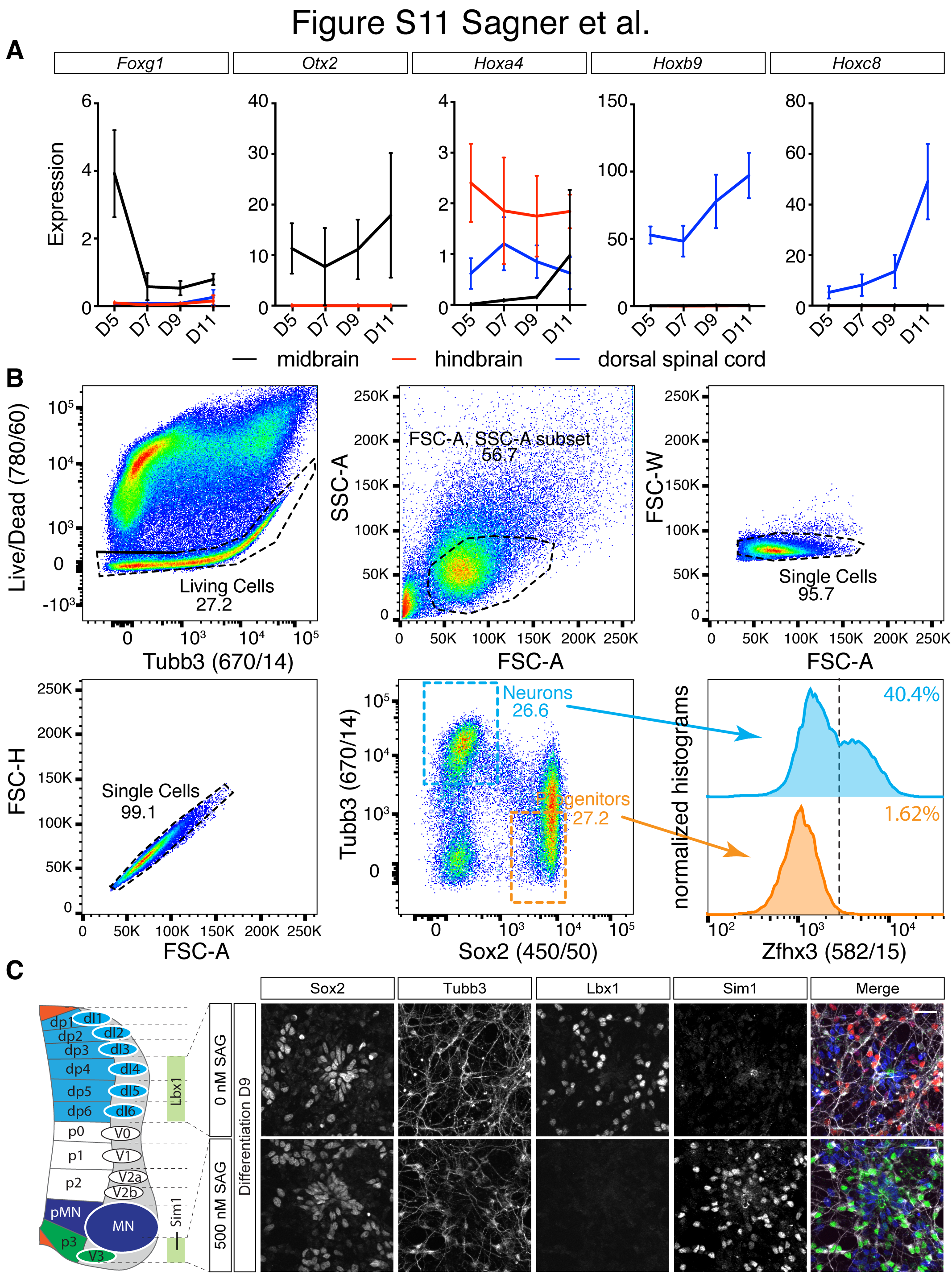

Supplement: S11 Fig — (A) RT-qPCR analysis of Foxg1, Otx2, Hoxa4, Hoxb9, and Hoxc8 reveals the generation of neurons and progenitors with different axial identities in the in vitro differentiations. For underlying data, see S4 Data. (B) Gating strategy for the quantification of the expression of different markers in neurons and progenitors by flow cytometry. Living cells were identified based on Infrared Life/Dead stain. Gating on single cells was achieved using forward and side scatter as indicated. Progenitors and neurons were discriminated based on the progenitor marker Sox2 and neuronal beta-tubulin (Tubb3). To quantify the proportion of neurons expressing Onecut2, Zfhx3, and Neurod2, an intensity threshold was applied to each sample that was exceeded by 1%–2% of progenitors. The same threshold was then applied to neurons in the same sample, and the percentage of neurons exceeding this threshold was counted as positive. As Nfia is expressed in neurons and progenitors, a global threshold was applied to quantify the proportion of neurons and progenitors expressing Nfia. (C) Characterization of dorsal and ventral spinal cord differentiations by immunostaining. Under dorsal conditions, most neurons express the TF Lbx1, which is expressed in dI4-dI6 neurons generated in the intermediate dorsal part of the spinal cord. Under ventral conditions, neurons express the V3 interneuron marker Sim1. Scale bars in C = 25 μm. MN, motor neuron; RT-qPCR, real-time quantitative polymerase chain reaction; SAG, Shh pathway agonist; TF, transcription factor. (PNG) [file pbio.3001450.s011.png]

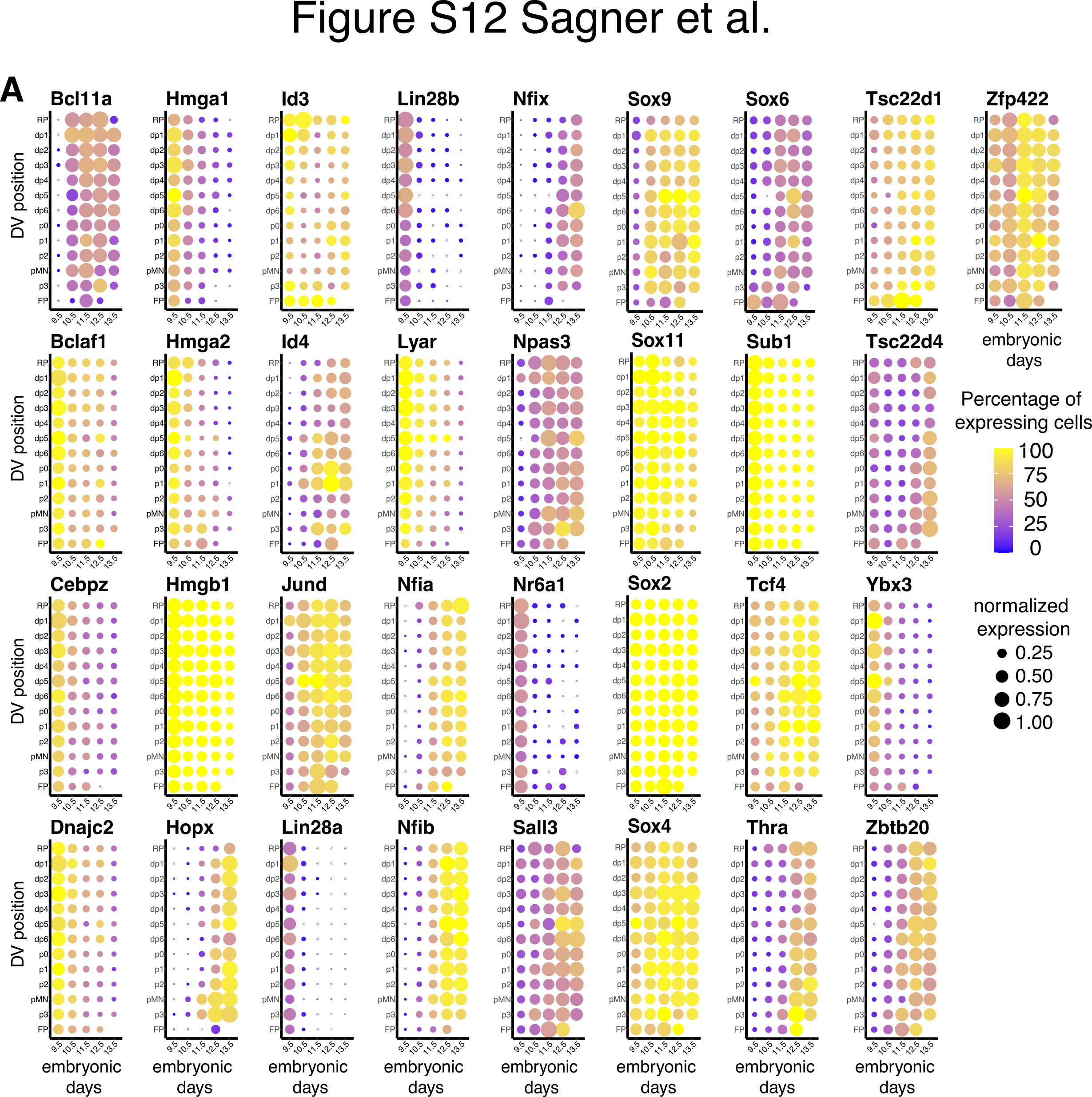

Supplement: S12 Fig — (A) Spatial and temporal expression of the 33 differentially expressed TFs during the neurogenic period in spinal cord neural progenitors. DV, dorsal–ventral; TF, transcription factor. (PNG) [file pbio.3001450.s012.png]

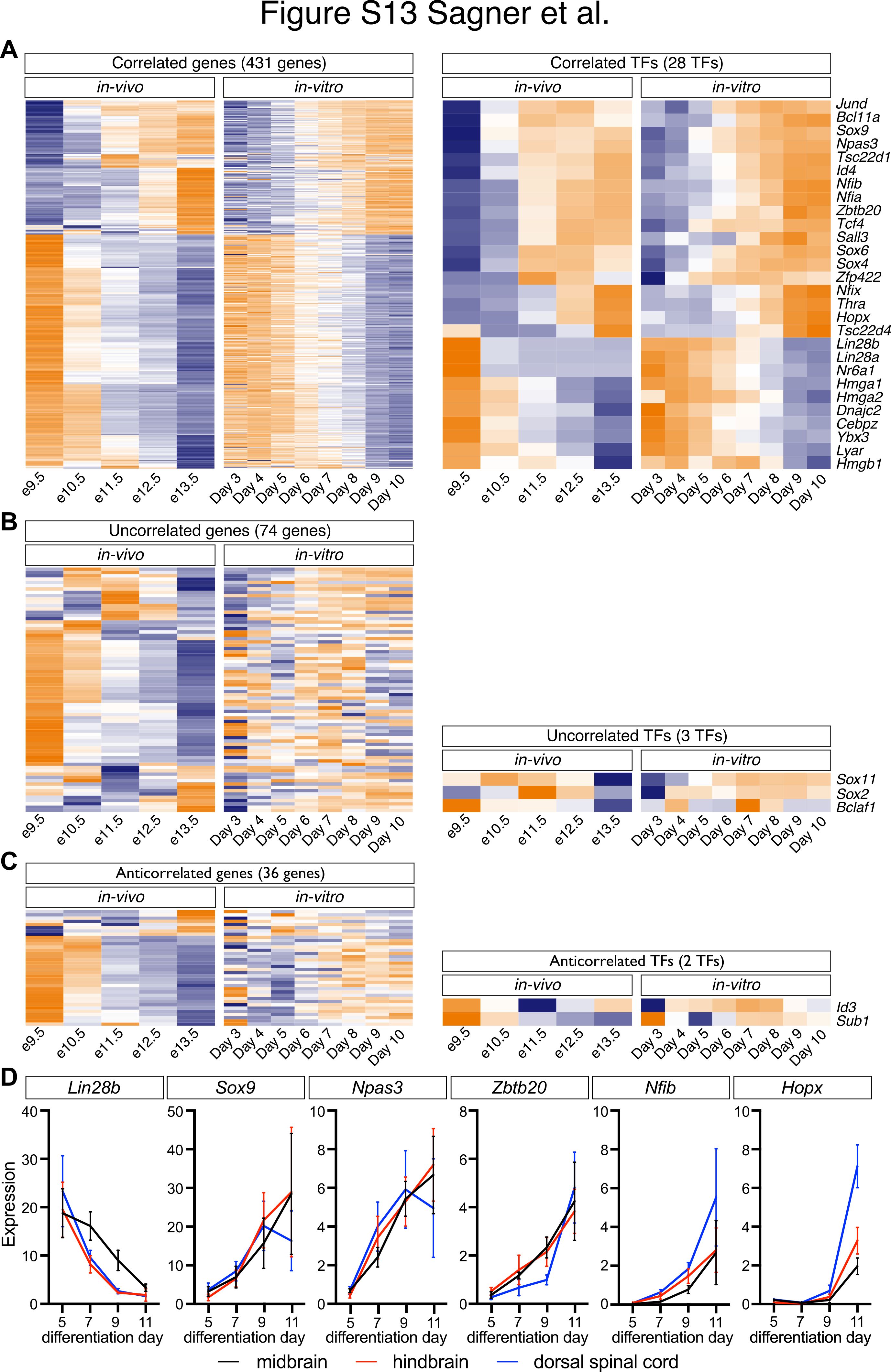

Supplement: S13 Fig — (A-C) Expression dynamics of correlated (A), uncorrelated (B), and anticorrelated (C) genes (left) and TFs (right) in embryonic progenitors (left plots) and RNAseq data from the in vitro differentiations (right plots). Heatmap shows log-scaled and z-scored gene expression values. Pearson correlation values are provided in S5 Data. (D) RT-qPCR analysis for Lin28b, Sox9, Npas3, Zbtb20, Nfib, and Hopx from days 5–11 in in vitro generated differentiations with different axial identities reveals that temporal patterning is conserved in vitro. Underlying data are included in S4 Data. RT-qPCR, real-time quantitative polymerase chain reaction; TF, transcription factor. (PNG) [file pbio.3001450.s013.png]

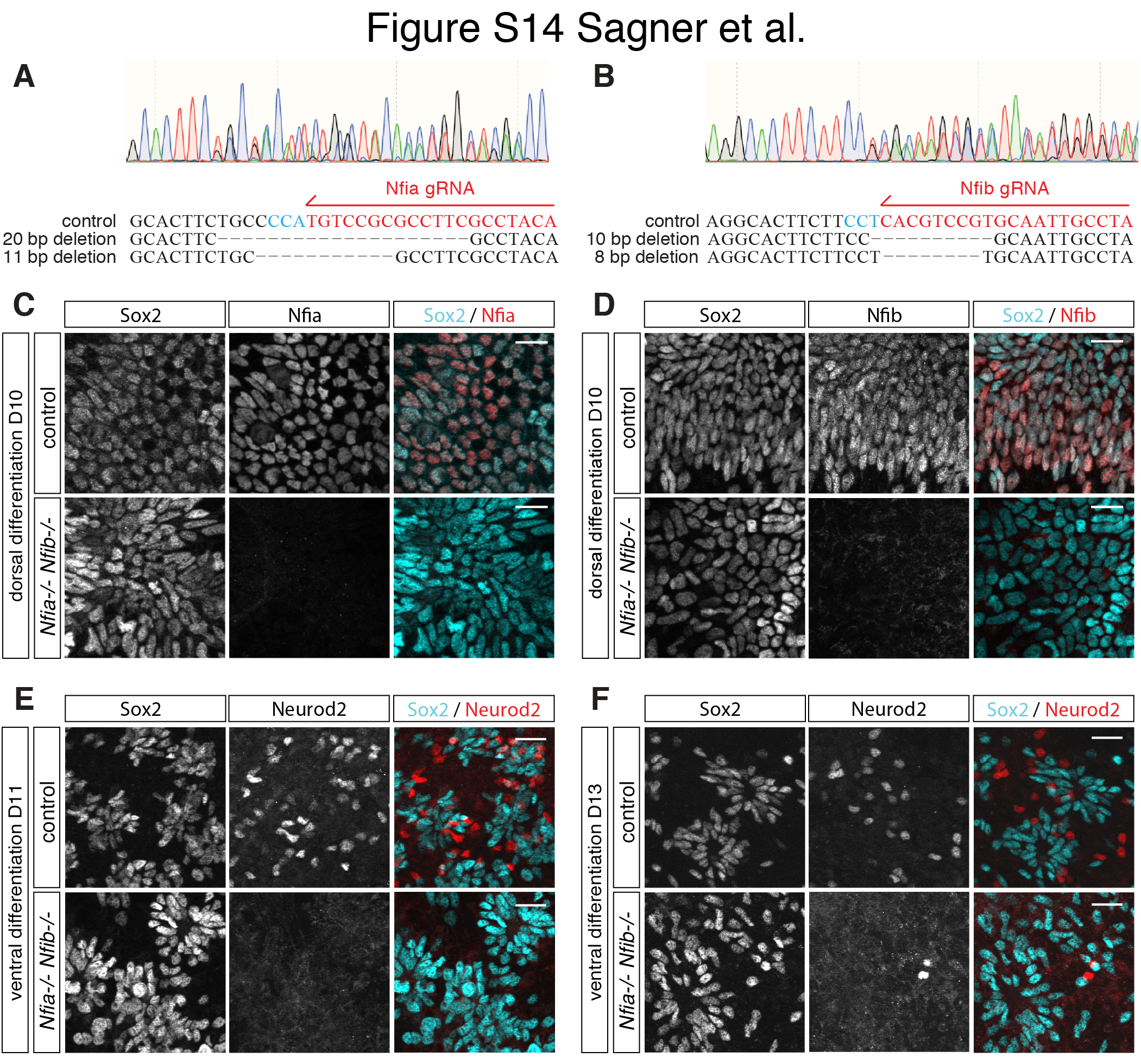

Supplement: S14 Fig — (A, B) Engineering of a Nfia; Nfib double-mutant ES cell line by CRISPR/Cas9-mediated mutagenesis. Introduction of double heterozygous frameshift mutations in both genes was validated by Sanger sequencing. (C, D) Loss of Nfia (C) and Nfib (D) immunostaining in neural progenitors generated from Nfia; Nfib double-mutant ES cells in dorsal differentiations at D10. (E, F) Reduced number of Neurod2-positive neurons in ventral differentiations of Nfia; Nfib double mutants compared to controls at D11 (E) and D13 (F) revealed by immunostaining. Scale bars in C-F = 20 μm. ES, embryonic stem. (PNG) [file pbio.3001450.s014.png]
