# Supplementary material for: A shared transcriptional code orchestrates temporal patterning of the central nervous system
Source: PLoS Biol. 2021 Nov 12;19(11):e3001450. doi: 10.1371/journal.pbio.3001450 (PMC8612522; doi:10.1371/journal.pbio.3001450)
Supplement: S1 Text — (PDF) [file pbio.3001450.s023.pdf]

Supplemental Experimental Procedures

Pseudotemporal ordering of neuronal populations in the spinal cord, hindbrain and forebrain

This documents details the steps for the generation of pseudotemporal expression profiles described in "Temporal patterning of the central nervous system by a shared transcription factor code" by Sagner et al. 2021.

Connect loom file from La Manno et al. 2020.

```
## connect sc.loom file downloaded from mousebrain.org
sc.loom <- loomR::connect(
  filename = paste0(dir, "/input/dev_all.loom"),
  mode = "r+", skip.validate = TRUE
)

## Generate sc.meta file by extracting parameters from connected sc.loom file
sc.meta <- data.frame(
  sc.loom$col.attrs$Age[],
  sc.loom$col.attrs$PseudoAge[],
  sc.loom$col.attrs$Tissue[],
  sc.loom$col.attrs$PseudoTissue[],
  sc.loom$col.attrs$Class[],
  sc.loom$col.attrs$Clusters[],
  10000 / sc.loom$col.attrs$TotalUMI[],
  sc.loom$col.attrs$CellID[],
  sc.loom$col.attrs$SampleID[]
)

colnames(sc.meta) <- c(
  "age", "pseudoage", "tissue", "pseudotissue", "class", "clusters",
  "normalization_factor", "cellID", "sampleID"
)
```

Load the spinal cord scRNAseq data from Delile et al. 2019.

```
eset <- readRDS(paste0(dir, "/input/m_neural.rds"))
eset <- eset$expressionSet

rownames(Biobase::pData(eset)) <- gsub("-", ".", rownames(Biobase::pData(eset)))
colnames(Biobase::exprs(eset)) <- gsub("-", ".", colnames(Biobase::exprs(eset)))

mat <- Biobase::exprs(eset)
rownames(mat) <- Biobase::fData(eset)[, "external_gene_name"]
```

```r
pbmc <- CreateSeuratObject(
  counts = mat,
  meta.data = Biobase::pData(eset),
  project = "MouseSpinalCordAtlas"
)

pbmc[["percent.mt"]] <- PercentageFeatureSet(pbmc, pattern = "^mt-")

pbmc <- pbmc %>%
  subset(subset = nFeature_RNA > 600 & nFeature_RNA < 6000 & percent.mt < 6) %>%
  SCTransform(., vars.to.regress = "cells_samples") %>%
  NormalizeData(verbose = FALSE, assay = "SCT") %>%
  ScaleData(verbose = FALSE, assay = "SCT")
```

```r
sc <- plot.expression.dynamics.from.Seurat(
  input = pbmc,
  genes = c("Onecut2", "Pou2f2", "Zfhx3", "Zfhx4", "Nfia", "Nfib", "Neurod2", "Neurod6"),
  time = "timepoint",
  title = "Spinal cord",
  from.loom = FALSE,
  colors = c("black", "orange", "darkred", "darkred", "steelblue", "steelblue", "limegreen", "limegreen
  linetype = c("solid", "solid", "solid", "dashed", "solid", "dashed", "solid", "dashed")
)

sc
```

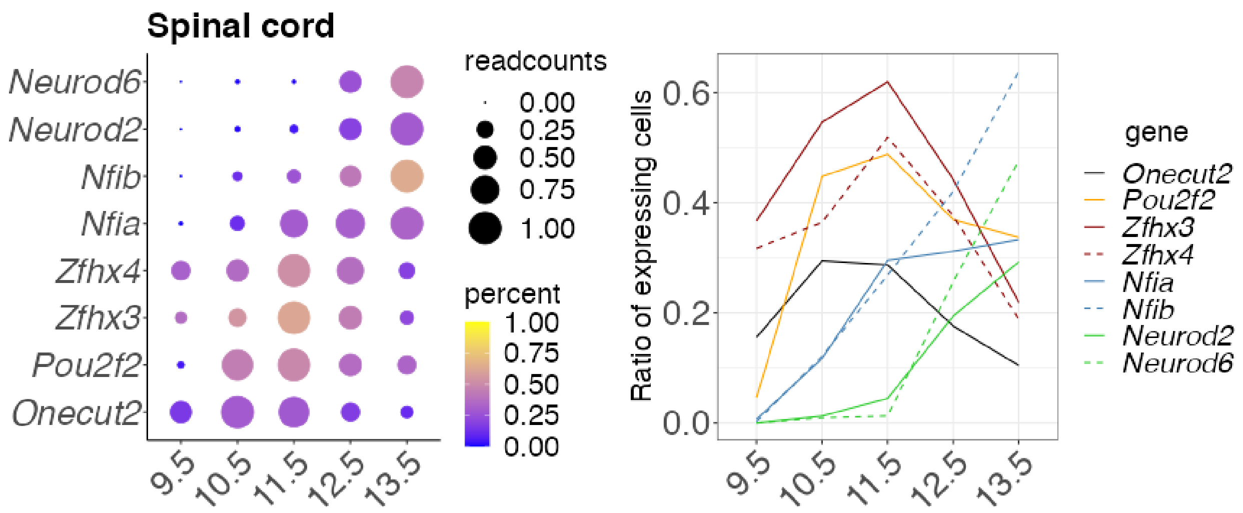

Pseudotemporal gene expression analysis of V2b neurons

Subset the data to V2b neurons and generate overview plots.

```r
### Pseudotemporal ordering of V2b neurons ----

V2.neurons <- pbmc %>%
  subset(subset = Type_step2 %in% c("V2b")) %>%
```

```
  NormalizeData(assay = "RNA") %>%
  ScaleData(assay = "RNA") %>%
  FindVariableFeatures(selection.method = "vst", verbose = FALSE) %>%
  RunPCA(npcs = 30, verbose = FALSE) %>%
  RunUMAP(reduction = "pca", dims = 1:30) %>%
  FindNeighbors(dims = 1:30) %>%
  FindClusters(resolution = 0.5)

cowplot::plot_grid(DimPlot(V2.neurons, reduction = "umap", group.by = "timepoint"),
  DimPlot(V2.neurons, reduction = "umap", group.by = "seurat_clusters"),
  FeaturePlot(V2.neurons, features = c("Ascl1", "Gata2", "Gata3", "Onecut2")),
  FeaturePlot(V2.neurons, features = c("Zfhx3", "Zfhx4", "Nfia", "Nfib")),
  nrow = 2
)
```

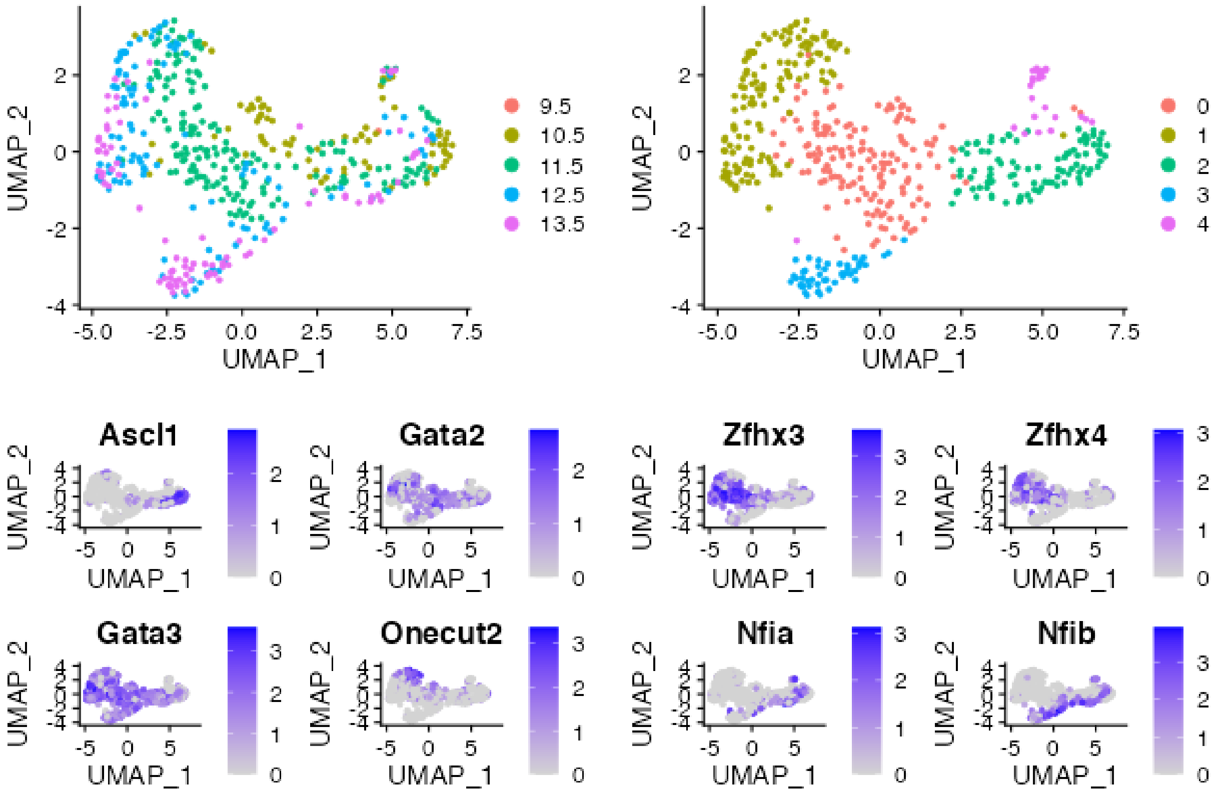

Pseudotemporal ordering using Slingshot

```
sds <- slingshot(Embeddings(V2.neurons, "umap"),
  clusterLabels = V2.neurons$seurat_clusters,
  start.clus = 2, end.clus = c(1, 3), stretch = 0
)

cell_colors_clust <- cell_pal(V2.neurons$seurat_clusters, hue_pal())

plot.pseudotime.curves(pal = colorRampPalette(c("blue", "red", "yellow"))(100))
```

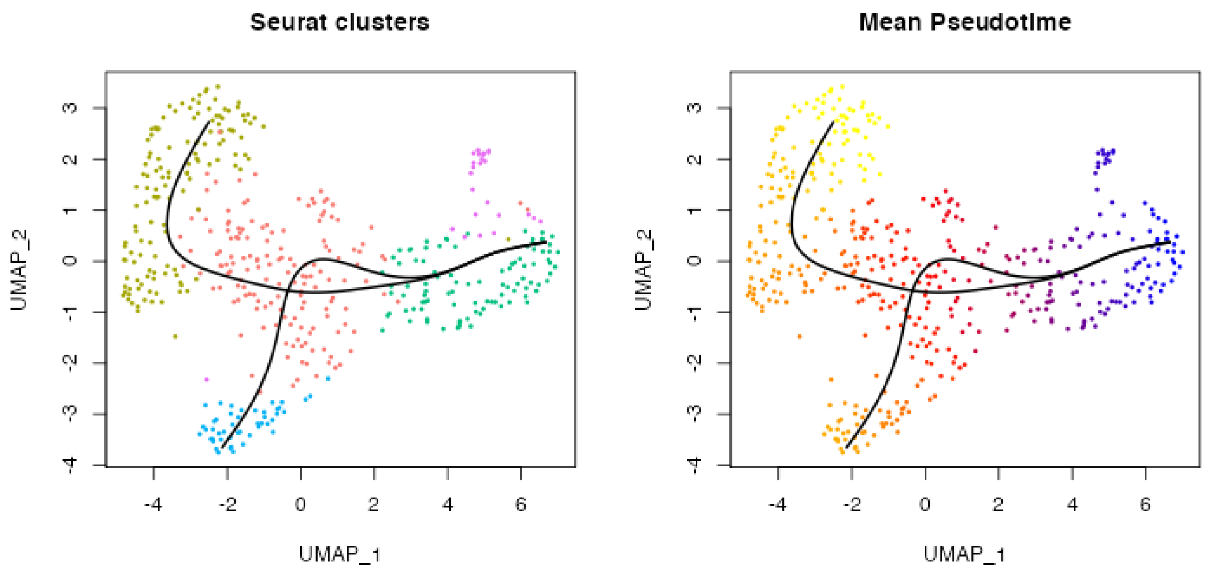

Plot pseudotime profiles of V2b neurons

```
plot.gene.in.pseudotime(
  gene = c(
    "Ascl1", "Tubb3", "Onecut2", "Zfhx3", "Zfhx4", "Nfia", "Nfib", "Neurod2", "Neurod6"
  ),
  pt.mtx = slingPseudotime(sds),
  seurat.object = V2.neurons,
  exclude.timepoint = c(9.5),
  exclude.curve = NULL,
  plot.title = "V2b neurons"
)
```

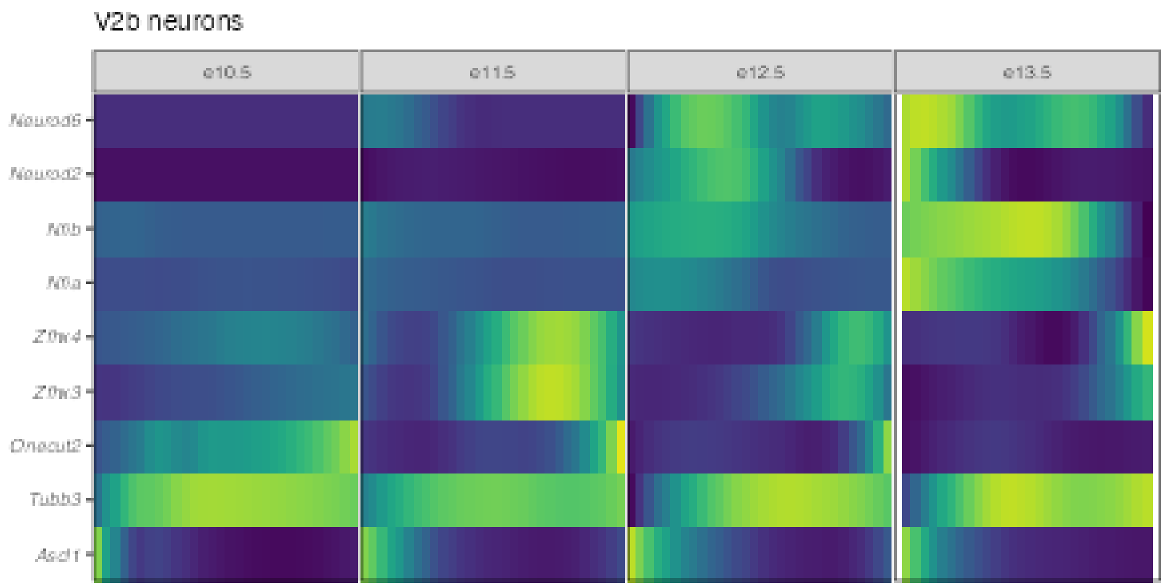

```r
plot.expression.dynamics.from.Seurat(
  input = V2.neurons,
  genes = c("Onecut2", "Pou2f2", "Zfhx3", "Zfhx4", "Nfia", "Nfib", "Neurod2", "Neurod6"),
  time = "timepoint",
  title = "V2b neurons"
)
```

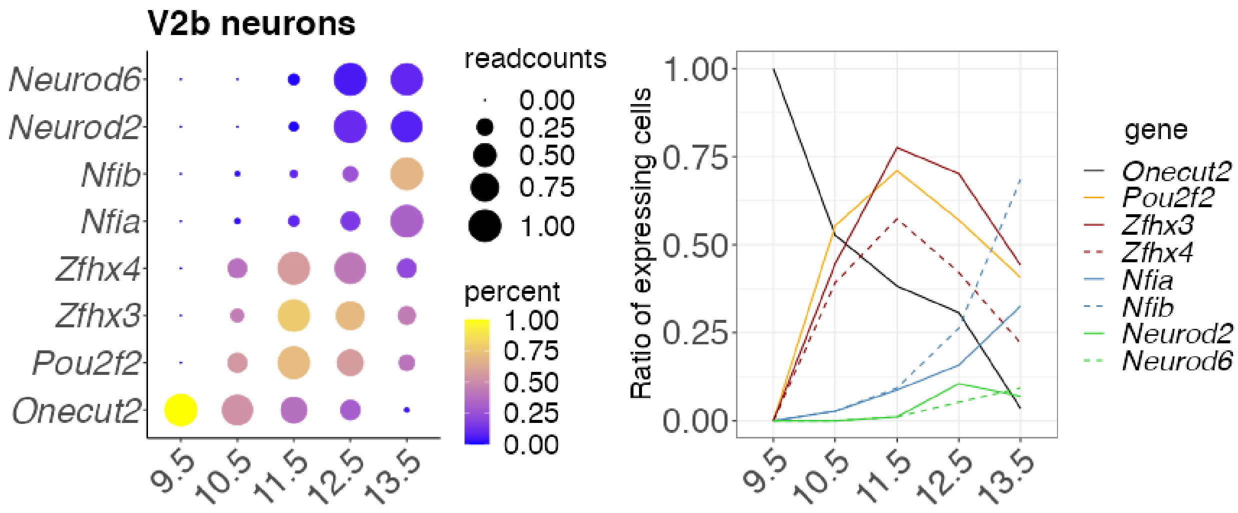

Pseudotemporal gene expression analysis of V2a neurons

Subset data to V2a neurons and generate overview plots.

```r
### Pseudotemporal ordering of V2b neurons ----

V2.neurons <- pbmc %>%
  subset(subset = Type_step2 %in% c("V2a")) %>%
  NormalizeData(assay = "RNA") %>%
  ScaleData(assay = "RNA") %>%
  FindVariableFeatures(selection.method = "vst", verbose = FALSE) %>%
  RunPCA(npcs = 30, verbose = FALSE) %>%
  RunUMAP(reduction = "pca", dims = 1:30) %>%
  FindNeighbors(dims = 1:30) %>%
  FindClusters(resolution = 0.5)

cowplot::plot_grid(DimPlot(V2.neurons, reduction = "umap", group.by = "timepoint"),
  DimPlot(V2.neurons, reduction = "umap", group.by = "seurat_clusters"),
  FeaturePlot(V2.neurons, features = c("Ascl1", "Vsx1", "Vsx2", "Onecut2")),
  FeaturePlot(V2.neurons, features = c("Zfhx3", "Zfhx4", "Nfia", "Nfib")),
  nrow = 2
)
```

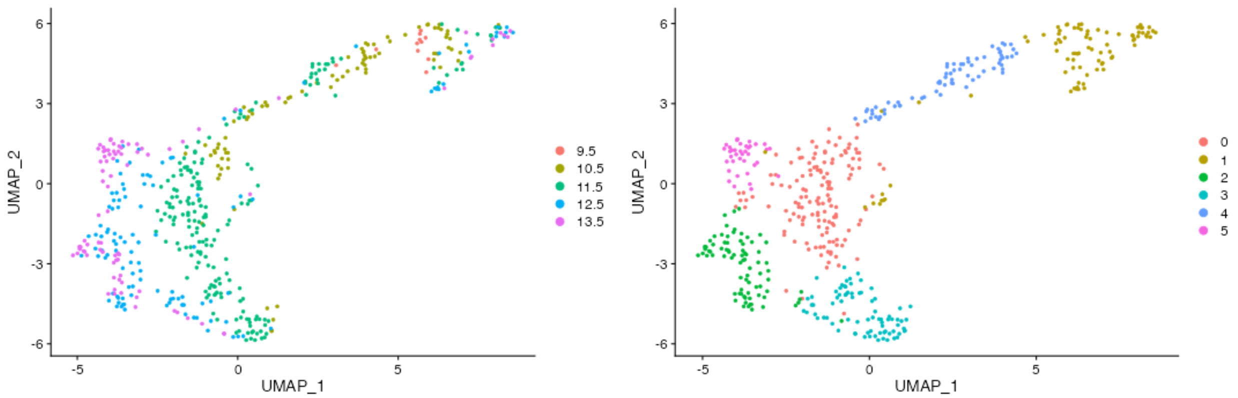

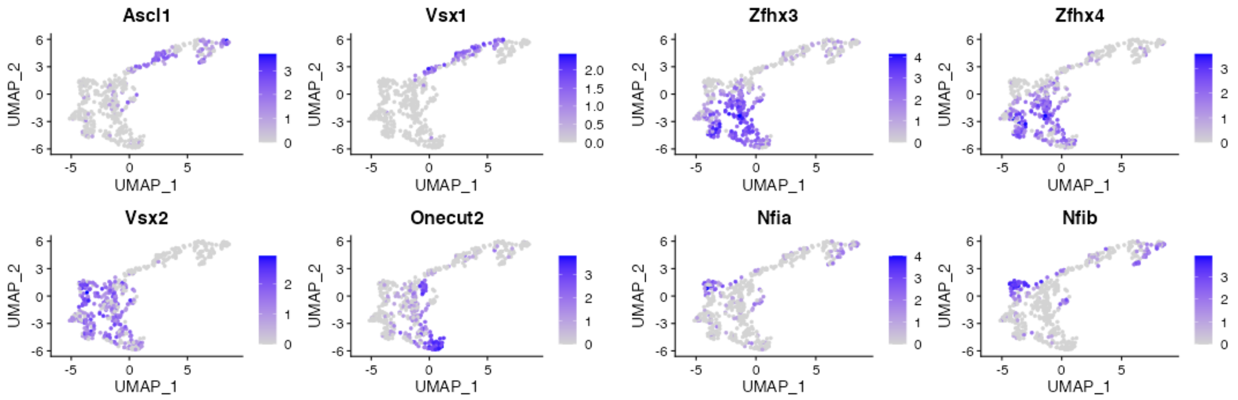

Pseudotemporal ordering using Slingshot

```
sds <- slingshot(Embeddings(V2.neurons, "umap"),
  clusterLabels = V2.neurons$seurat_clusters,
  start.clus = 1, end.clus = c(2, 3, 5), stretch = 0
)

cell_colors_clust <- cell_pal(V2.neurons$seurat_clusters, hue_pal())

plot.pseudotime.curves(pal = colorRampPalette(c("blue", "red", "yellow"))(100))
```

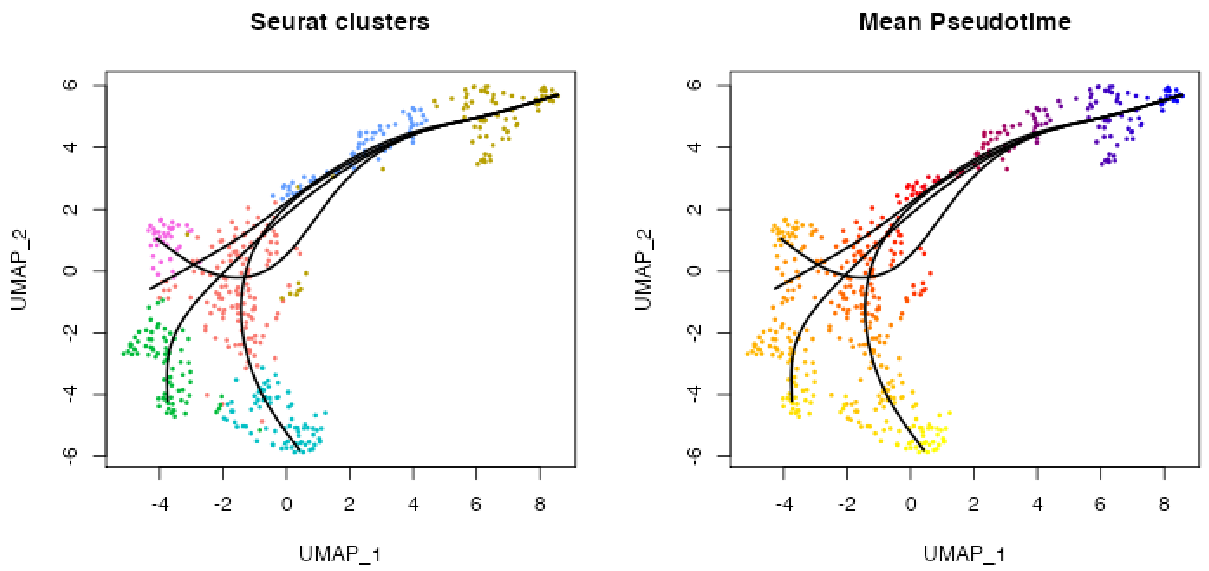


```
plot.gene.in.pseudotime(
  gene = c(
    "Ascl1", "Tubb3", "Onecut2", "Zfhx3", "Zfhx4", "Nfia", "Nfib", "Neurod2", "Neurod6"
  ),
  pt.mtx = slingPseudotime(sds),
  seurat.object = V2.neurons,
  exclude.timepoint = c(9.5),
  exclude.curve = NULL,
  plot.title = "V2a neurons"
)
```

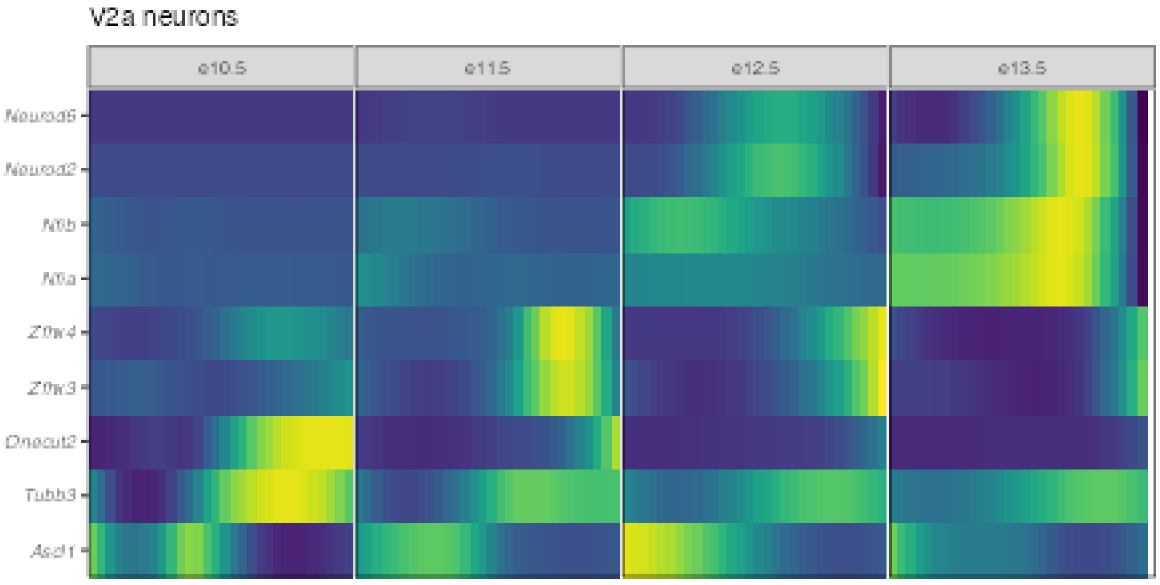


```
plot.expression.dynamics.from.Seurat(
  input = V2.neurons,
  genes = c("Onecut2", "Pou2f2", "Zfhx3", "Zfhx4", "Nfia", "Nfib", "Neurod2", "Neurod6"),
```

```
  time = "timepoint",
  title = "V2a neurons"
)
```

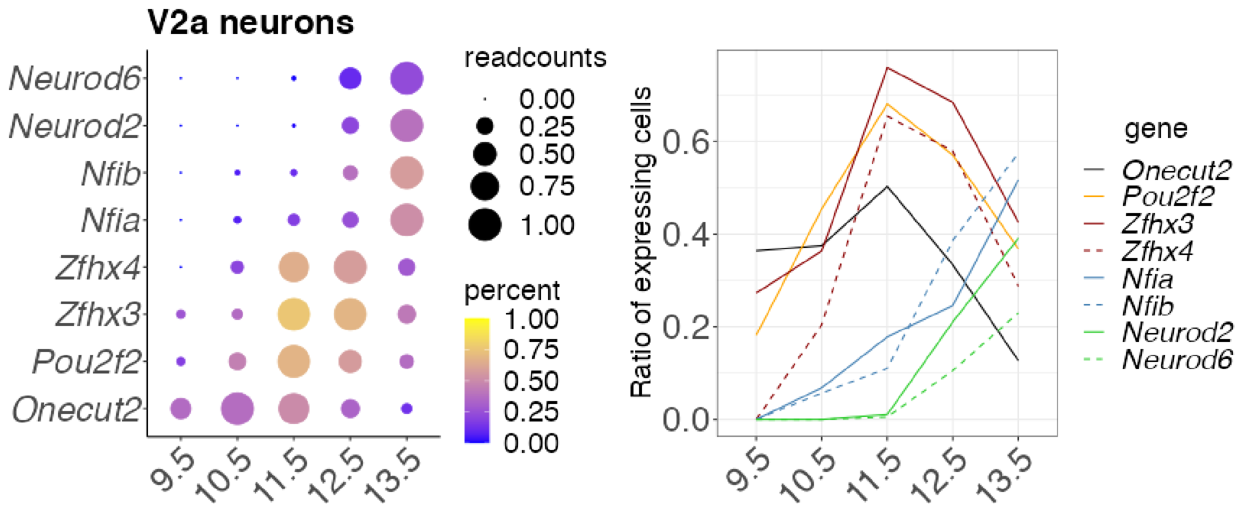

Pseudotemporal gene expression analysis of dI1 neurons

Subset data to dI1 neurons.

```
### Pseudotemporal ordering of V2b neurons ----

V2.neurons <- pbmc %>%
  subset(subset = Type_step2 %in% c("dI1")) %>%
  NormalizeData(assay = "RNA") %>%
  ScaleData(assay = "RNA") %>%
  FindVariableFeatures(selection.method = "vst", verbose = FALSE) %>%
  RunPCA(npcs = 30, verbose = FALSE) %>%
  RunUMAP(reduction = "pca", dims = 1:30) %>%
  FindNeighbors(dims = 1:30) %>%
  FindClusters(resolution = 0.5)

cowplot::plot_grid(DimPlot(V2.neurons, reduction = "umap", group.by = "timepoint"),
  DimPlot(V2.neurons, reduction = "umap", group.by = "seurat_clusters"),
  FeaturePlot(V2.neurons, features = c("Atoh1", "Lhx2", "Lhx9", "Onecut2")),
  FeaturePlot(V2.neurons, features = c("Zfhx3", "Zfhx4", "Nfia", "Nfib")),
  nrow = 2
)
```

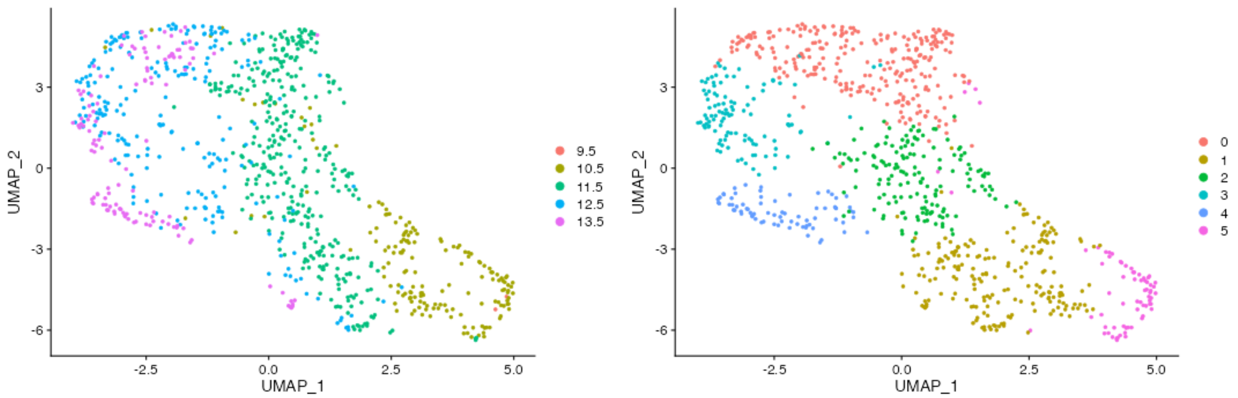

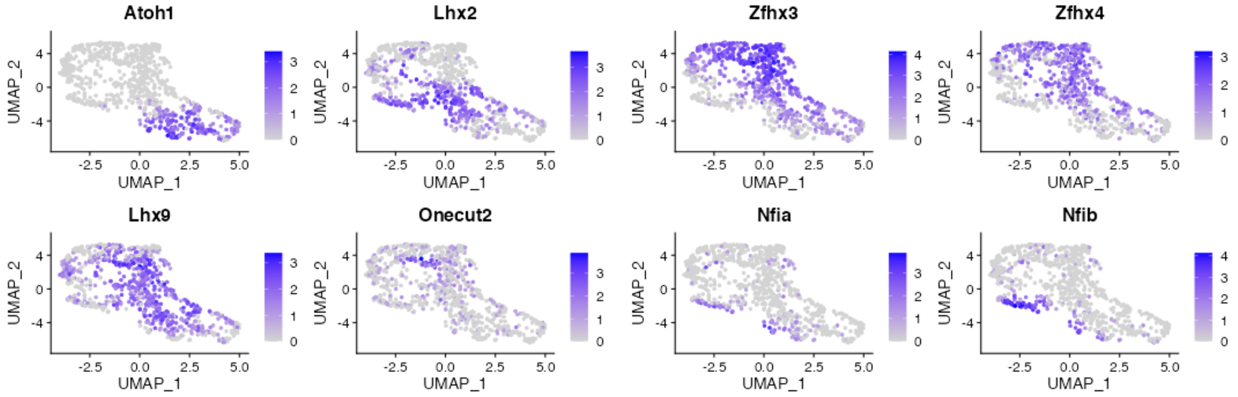

Pseudotemporal ordering using Slingshot

```
sds <- slingshot(Embeddings(V2.neurons, "umap"),
  clusterLabels = V2.neurons$seurat_clusters,
  start.clus = 5, end.clus = c(0, 3), stretch = 0
)

cell_colors_clust <- cell_pal(V2.neurons$seurat_clusters, hue_pal())

plot.pseudotime.curves(pal = colorRampPalette(c("blue", "red", "yellow"))(100))
```

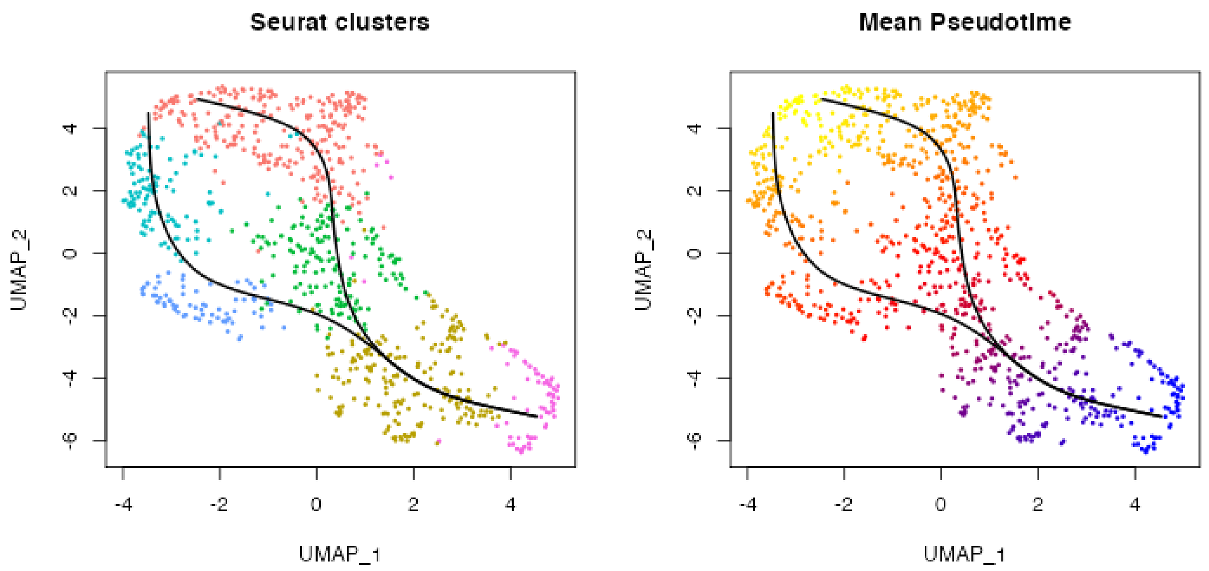

Plot pseudotime profiles of dI1 neurons

```
plot.gene.in.pseudotime(
  gene = c(
    "Atoh1", "Tubb3", "Onecut2", "Zfhx3", "Zfhx4", "Nfia", "Nfib", "Neurod2", "Neurod6"
  ),
  pt.mtx = slingPseudotime(sds),
  seurat.object = V2.neurons,
  exclude.timepoint = c(9.5),
  exclude.curve = NULL,
  plot.title = "dI1 neurons"
)
```

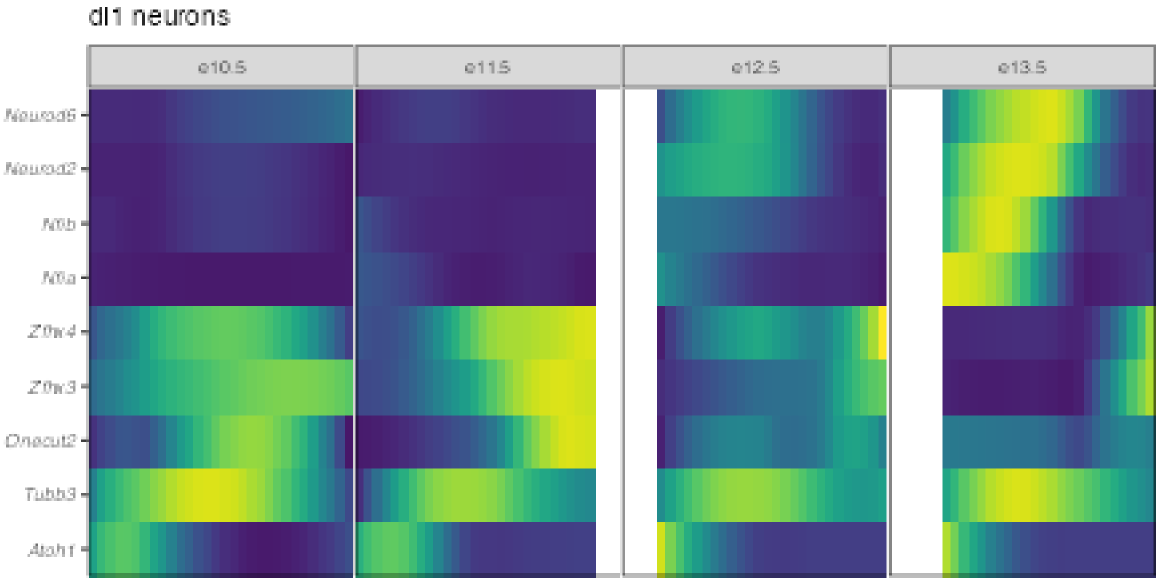

```
plot.expression.dynamics.from.Seurat(
  input = V2.neurons,
  genes = c("Onecut2", "Pou2f2", "Zfhx3", "Zfhx4", "Nfia", "Nfib", "Neurod2", "Neurod6"),
  time = "timepoint",
  title = "dI1 neurons"
)
```

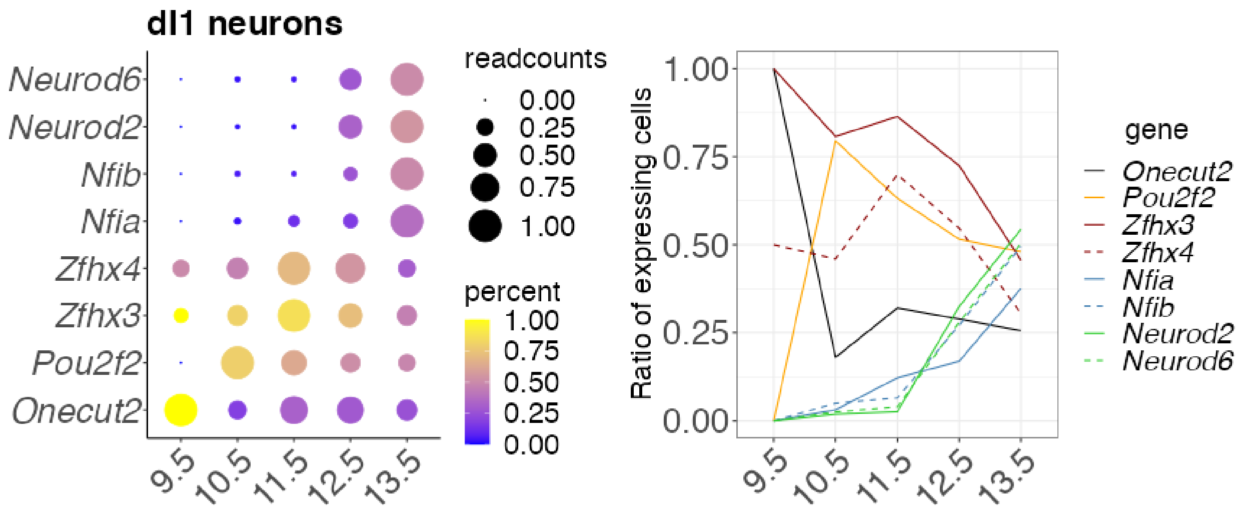

Pseudotemporal gene expression analysis of dI5 neurons

Subset data to dI5 neurons.

```
### Pseudotemporal ordering of V2b neurons ----

V2.neurons <- pbmc %>%
  subset(subset = Type_step2 %in% c("dI5")) %>%
  NormalizeData(assay = "RNA") %>%
  ScaleData(assay = "RNA") %>%
  FindVariableFeatures(selection.method = "vst", verbose = FALSE) %>%
  RunPCA(npcs = 30, verbose = FALSE) %>%
  RunUMAP(reduction = "pca", dims = 1:30) %>%
  FindNeighbors(dims = 1:30) %>%
  FindClusters(resolution = 0.5)

cowplot::plot_grid(DimPlot(V2.neurons, reduction = "umap", group.by = "timepoint"),
  DimPlot(V2.neurons, reduction = "umap", group.by = "seurat_clusters"),
  FeaturePlot(V2.neurons, features = c("Ascl1", "Lmx1b", "Tlx3", "Onecut2")),
  FeaturePlot(V2.neurons, features = c("Zfhx3", "Zfhx4", "Nfia", "Nfib")),
  nrow = 2
)
```

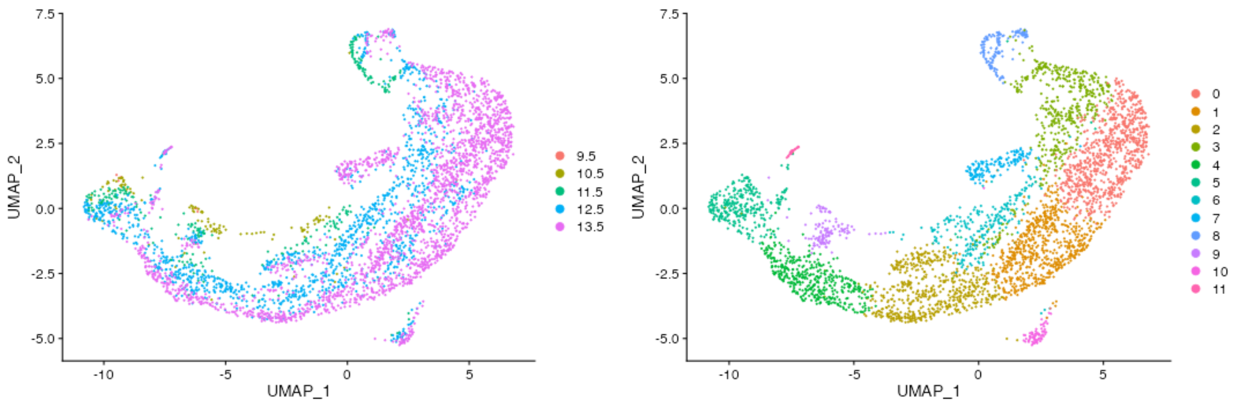

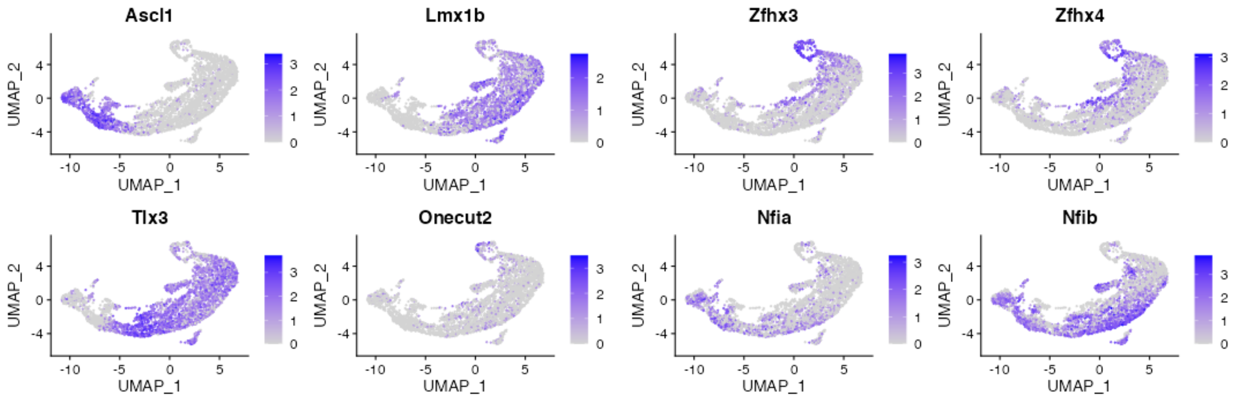

Pseudotemporal ordering using Slingshot. We exclude curve4 because it bends back on itself. Cells without pseudotime coordinates are shown in red.

```
sds <- slingshot(Embeddings(V2.neurons, "umap"),
  clusterLabels = V2.neurons$seurat_clusters,
  start.clus = 5, end.clus = 8, stretch = 0
)

cell_colors_clust <- cell_pal(V2.neurons$seurat_clusters, hue_pal())

plot.pseudotime.curves(
  pal = colorRampPalette(c("blue", "red", "yellow"))(100),
  exclude.curve = "curve4"
)
```

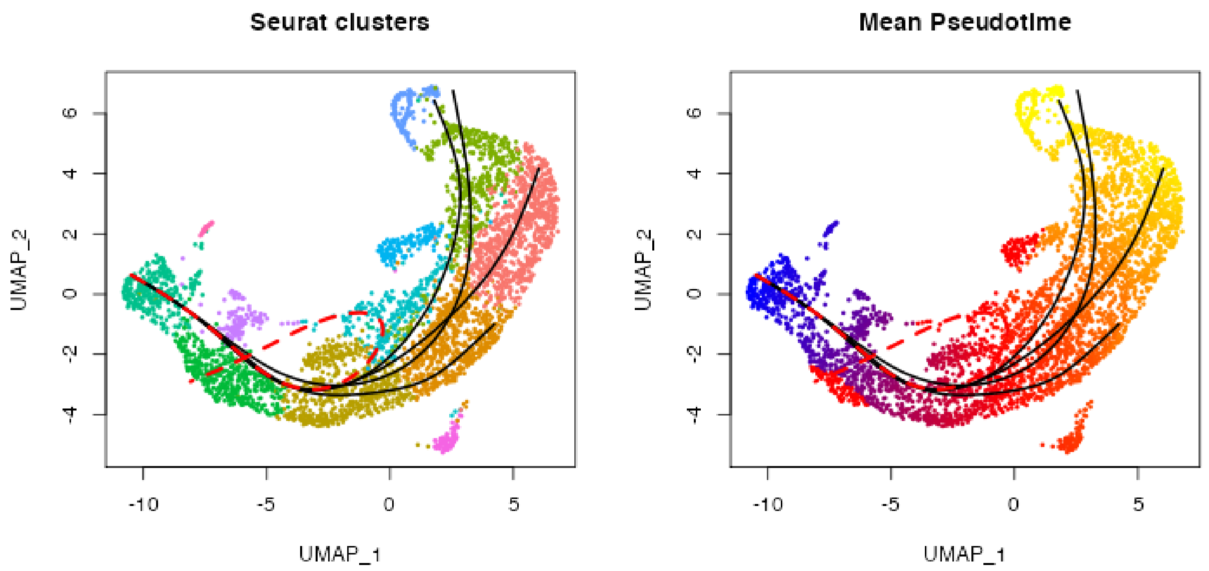

Plot pseudotime profiles of dI5 neurons

```
plot.gene.in.pseudotime(
  gene = c(
    "Ascl1", "Tubb3", "Onecut2", "Zfhx3", "Zfhx4", "Nfia", "Nfib", "Neurod2", "Neurod6"
  ),
  pt.mtx = slingPseudotime(sds),
  seurat.object = V2.neurons,
  exclude.timepoint = c(9.5),
  exclude.curve = "curve4",
  plot.title = "dI5 neurons"
)
```

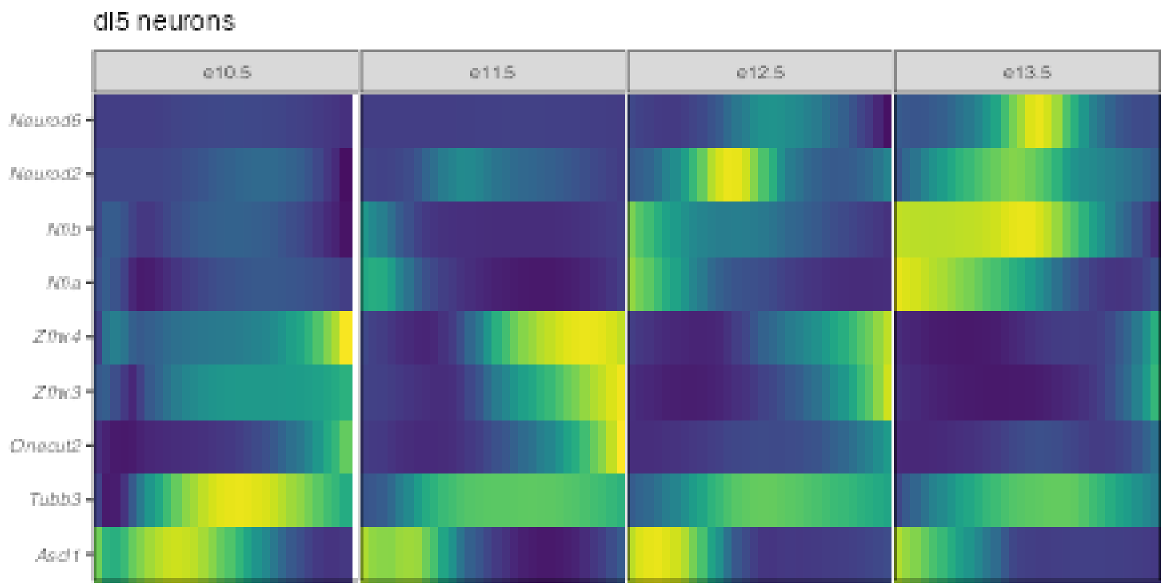

```r
plot.expression.dynamics.from.Seurat(
  input = V2.neurons,
  genes = c("Onecut2", "Pou2f2", "Zfhx3", "Zfhx4", "Nfia", "Nfib", "Neurod2", "Neurod6"),
  time = "timepoint",
  title = "dI5 neurons"
)
```

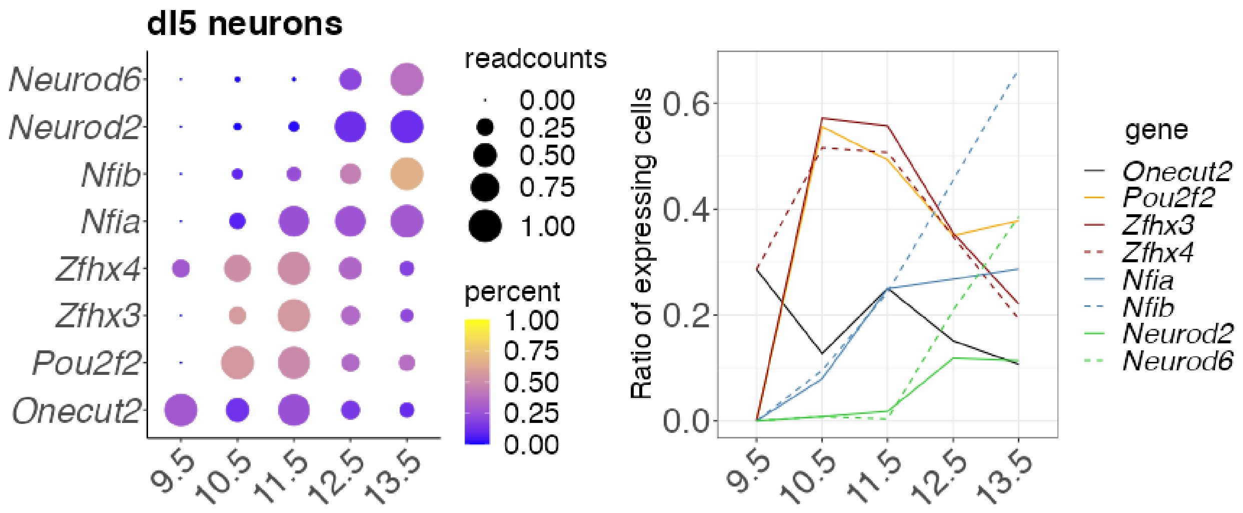

Load hindbrain scRNAseq data from La Manno et al. 2020

We subset the data to annotated hindbrain neurons and convert the data in a Seurat object. To identify which clusters define which neuronal populations, we plot the data on a UMAP.

```r
tissue <- "Hindbrain"
celltype <- "Neuron"
timepoints <- c("e9.0", "e10.0", "e11.0", "e12.0", "e12.5", "e13.0", "e13.5", "e14.0")

tissue.id <- which(grepl(tissue, unique(sc.loom$col.attrs$Tissue[])) == TRUE)
cell.id <- intersect(
  which(sc.meta$tissue %in% unique(sc.loom$col.attrs$Tissue[])[tissue.id] &
    sc.meta$class == celltype),
  which(sc.meta$age %in% timepoints)
)

exp.mat <- sc.loom[["matrix"]][cell.id, ]

colnames(exp.mat) <- sc.loom$row.attrs$Gene[]
rownames(exp.mat) <- sc.meta$cellID[cell.id]

hb.seurat <- CreateSeuratObject(
  counts = t(exp.mat),
  meta.data = sc.meta[cell.id, ] %>%
    as.tibble() %>%
    tibble::column_to_rownames("cellID")
)
```

```r
hb.seurat[["percent.mt"]] <- PercentageFeatureSet(hb.seurat, pattern = "^mt-")

hb.seurat <- hb.seurat %>%
  subset(subset = nFeature_RNA > 600 & nFeature_RNA < 6000 & percent.mt < 6) %>%
  SCTransform(vars.to.regress = "sampleID") %>%
  NormalizeData(verbose = FALSE) %>%
  ScaleData(verbose = FALSE) %>%
  FindVariableFeatures(selection.method = "vst", verbose = FALSE) %>%
  RunPCA(npcs = 30, verbose = FALSE) %>%
  RunUMAP(reduction = "pca", dims = 1:30) %>%
  FindNeighbors(dims = 1:30) %>%
  FindClusters(resolution = 0.5)

cowplot::plot_grid(DimPlot(hb.seurat, reduction = "umap", group.by = "age"),
  DimPlot(hb.seurat, reduction = "umap", group.by = "seurat_clusters"),
  FeaturePlot(hb.seurat, features = c("Slc17a6", "Gad2", "Tubb3", "Onecut2")),
  FeaturePlot(hb.seurat, features = c("Zfhx3", "Zfhx4", "Nfia", "Nfib")),
  FeaturePlot(hb.seurat, features = c("Pax2", "Lhx5", "Lhx1", "Isl1")),
  FeaturePlot(hb.seurat, features = c("Lhx2", "Lhx9", "Barhl1", "Lmx1b")),
  nrow = 2
)
```

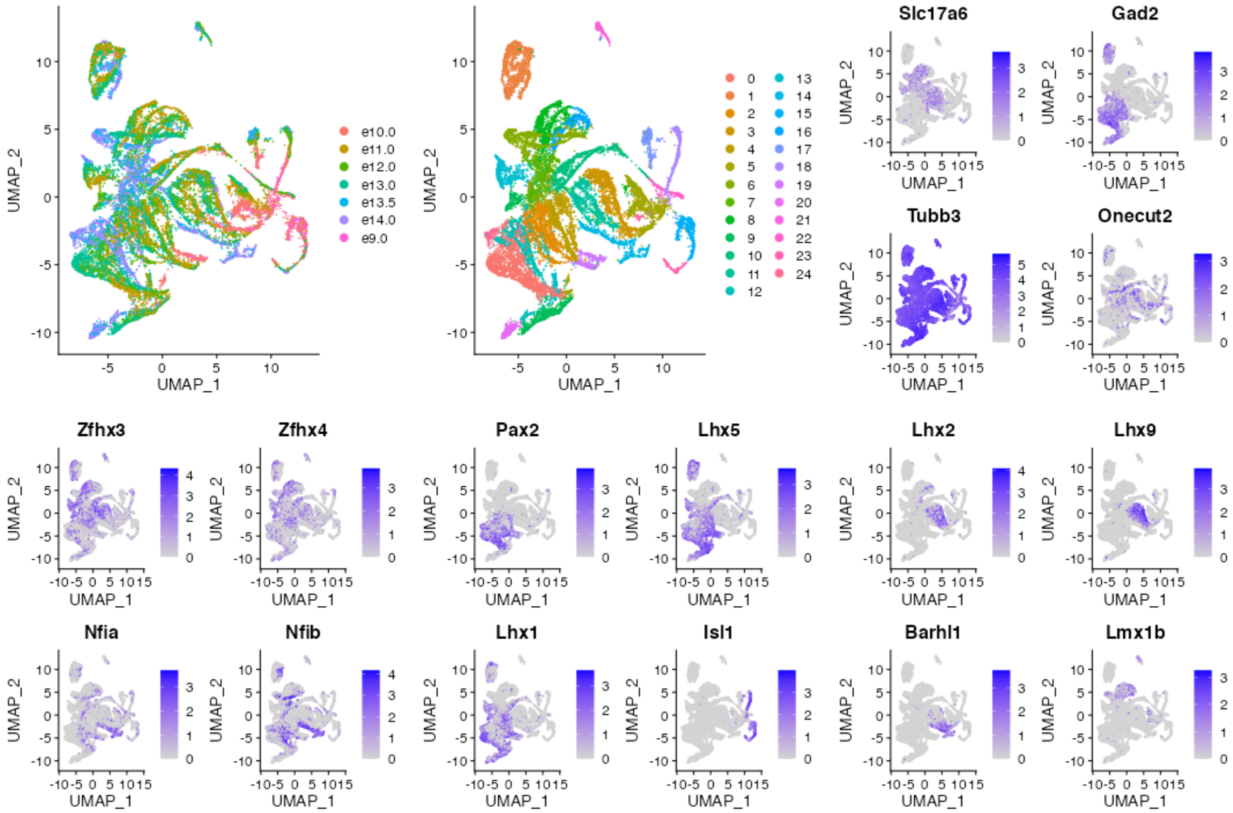


```r
hb <- plot.expression.dynamics.from.Seurat(
  input = hb.seurat,
  genes = c("Onecut2", "Pou2f2", "Zfhx3", "Zfhx4", "Nfia", "Nfib", "Neurod2", "Neurod6"),
  time = "age",
```

```
  title = "Hindbrain",
  from.loom = TRUE,
  colors = c("black", "orange", "darkred", "darkred", "steelblue", "steelblue", "limegreen", "limegreen
  linetype = c("solid", "solid", "solid", "dashed", "solid", "dashed", "solid", "dashed")
)

hb
```

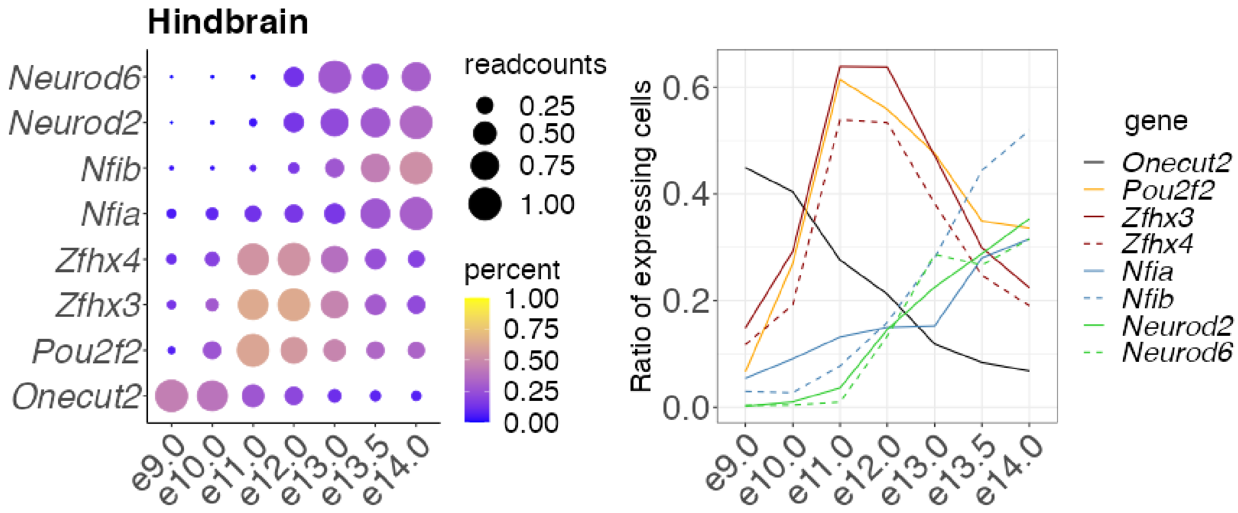

Pseudotemporal gene expression analysis of Pax2 neurons

```
cr.neurons <- hb.seurat %>%
  subset(subset = seurat_clusters %in% c(2, 4, 19)) %>%
  ScaleData(assay = "RNA") %>%
  FindVariableFeatures(selection.method = "vst", verbose = FALSE) %>%
  RunPCA(npcs = 30, verbose = FALSE) %>%
  RunUMAP(reduction = "pca", dims = 1:30) %>%
  FindNeighbors(dims = 1:30) %>%
  FindClusters(resolution = 0.5)

DimPlot(cr.neurons, reduction = "umap", group.by = "seurat_clusters")
```

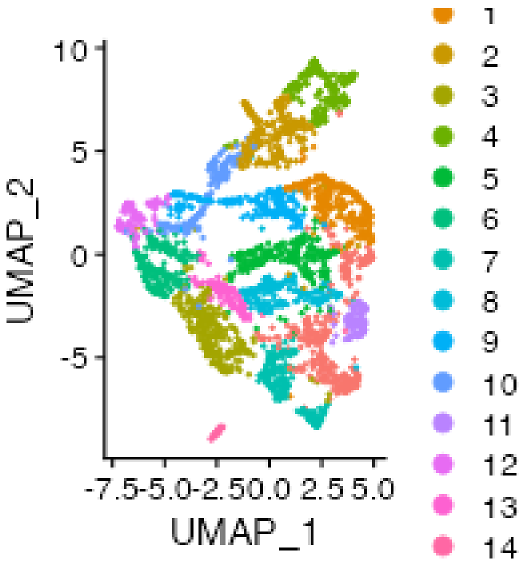

We remove cluster 14 because it is not connected to the remaining clusters

```
cr.neurons <- subset(cr.neurons, subset = seurat_clusters != 14)

cowplot::plot_grid(DimPlot(cr.neurons, reduction = "umap", group.by = "age"),
  DimPlot(cr.neurons, reduction = "umap", group.by = "seurat_clusters"),
  FeaturePlot(cr.neurons, features = c("Neurog1", "Neurog2", "Tubb3", "Gad2")),
  FeaturePlot(cr.neurons, features = c("Lbx1", "En1", "Pax2", "Pax7")),
  FeaturePlot(cr.neurons, features = c("Onecut2", "Zfhx3", "Zfhx4", "Nfib")),
  FeaturePlot(cr.neurons, features = c("Neurod2", "Neurod6", "Evx1", "Gata3")),
  nrow = 2
)
```

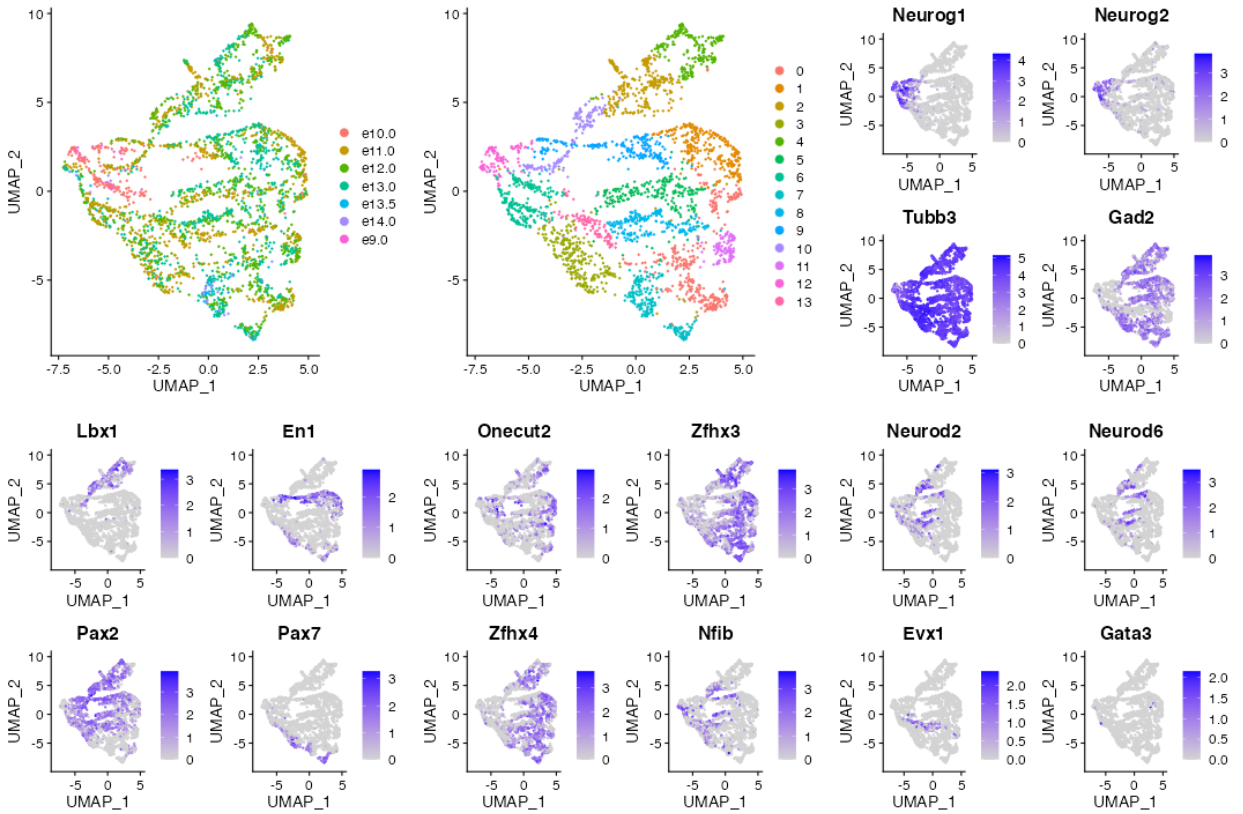

This cluster encompasses multiple neuronal subtypes. We nevertheless perform pseudotemporal ordering using Slingshot. We exclude curve3 because it crosses other curves.

```
sds <- slingshot(Embeddings(cr.neurons, "umap"),
  clusterLabels = cr.neurons$seurat_clusters,
  start.clus = 12, end.clus = c(1, 4, 7, 11), stretch = 0
)

cell_colors_clust <- cell_pal(cr.neurons$seurat_clusters, hue_pal())

plot.pseudotime.curves(exclude.curve = "curve3")
```

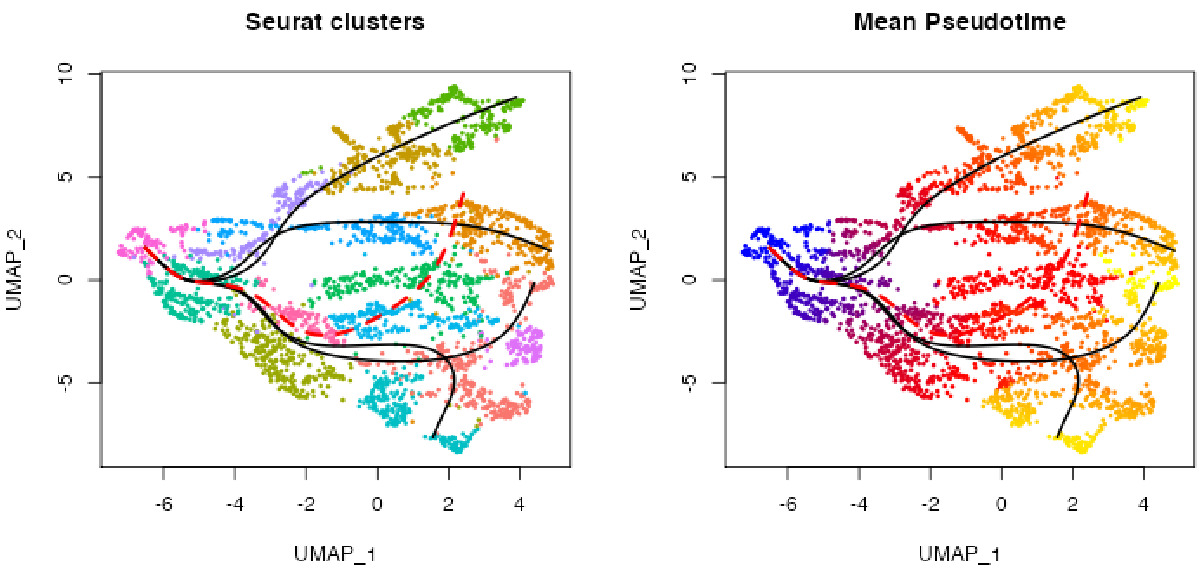

We exclude e9.0 because there are very few neurons for this timepoint.

Table 1: Number of cells / timepoint

e10.0	e11.0	e12.0	e13.0	e13.5	e14.0	e9.0
168	838	1097	799	48	41	32

```
plot.gene.in.pseudotime.loom(
  gene = c(
    "Neurog2", "Tubb3", "Onecut2", "Zfhx3", "Zfhx4", "Nfia", "Nfib", "Neurod2", "Neurod6"
  ),
  pt.mtx = slingPseudotime(sds),
  seurat.object = cr.neurons,
  exclude.timepoint = c("e9.0"),
  exclude.curve = c("curve3"),
  plot.title = "Hindbrain inhibitory neurons"
)
```

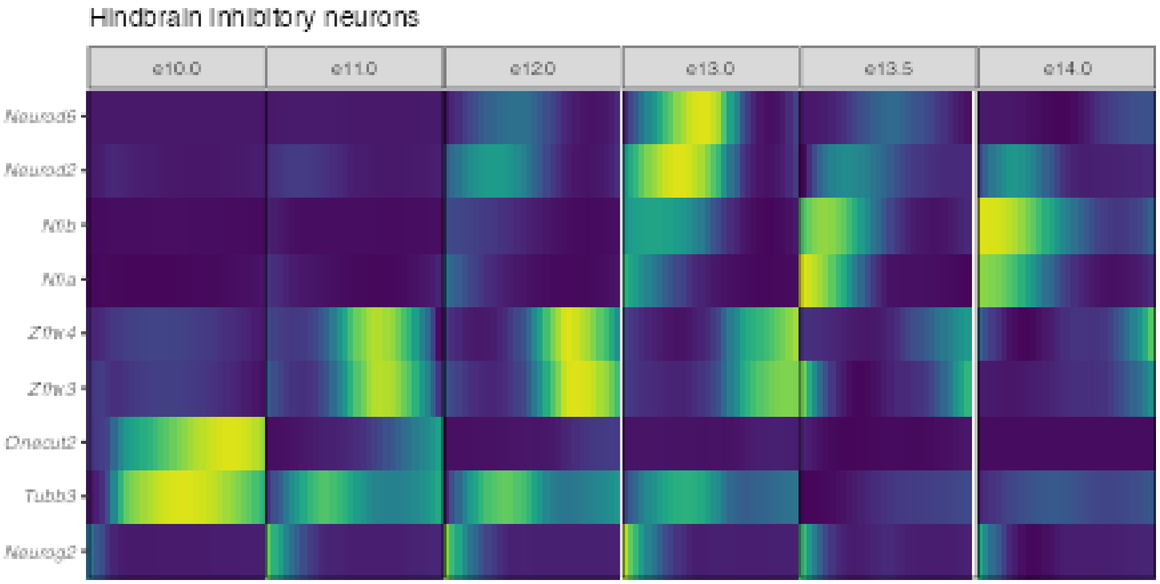


```
plot.expression.dynamics.from.Seurat(
  input = cr.neurons,
  genes = c("Onecut2", "Pou2f2", "Zfhx3", "Zfhx4", "Nfia", "Nfib", "Neurod2", "Neurod6"),
  time = "age",
  title = "Hindbrain inhibitory neurons",
  from.loom = TRUE
)
```

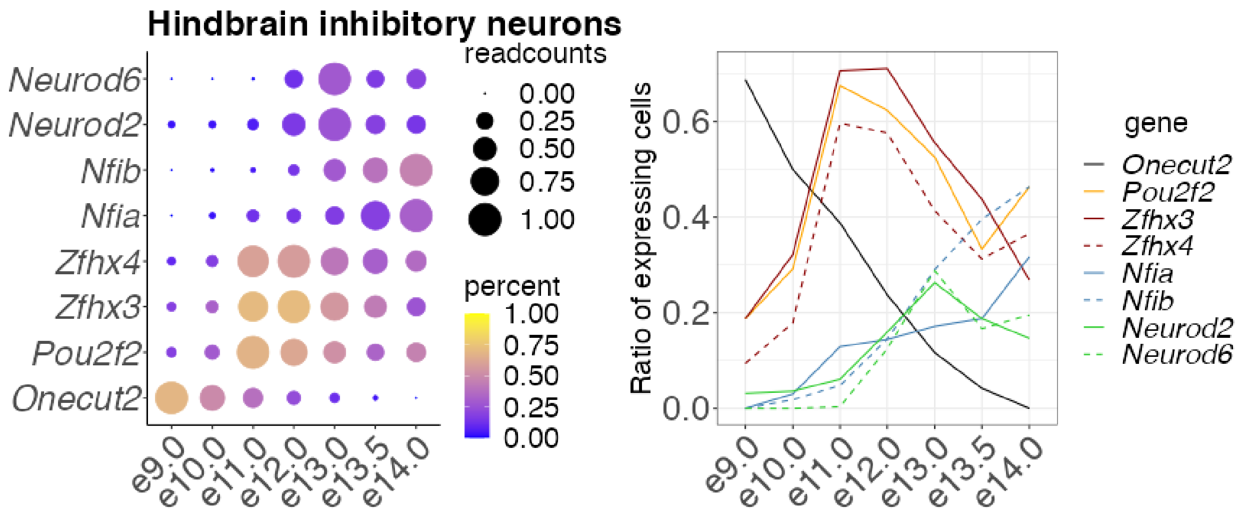

Pseudotemporal gene expression analysis of hindbrain V1 neurons

We further subset hindbrain inhibitory neurons to only include the lineage leading to En1-positive neurons.

```
V1.neurons <- subset(cr.neurons, subset = seurat_clusters %in% c(1, 9, 12)) %>%
  ScaleData(assay = "RNA") %>%
  FindVariableFeatures(selection.method = "vst", verbose = FALSE) %>%
  RunPCA(npcs = 30, verbose = FALSE) %>%
```

```
  RunUMAP(reduction = "pca", dims = 1:30) %>%
  FindNeighbors(dims = 1:30) %>%
  FindClusters(resolution = 0.5)

cowplot::plot_grid(DimPlot(V1.neurons, reduction = "umap", group.by = "age"),
  DimPlot(V1.neurons, reduction = "umap", group.by = "seurat_clusters"),
  FeaturePlot(V1.neurons, features = c("Neurog1", "Neurog2", "Tubb3", "Gad2")),
  FeaturePlot(V1.neurons, features = c("Lbx1", "En1", "Pax2", "Pax7")),
  FeaturePlot(V1.neurons, features = c("Onecut2", "Zfhx3", "Zfhx4", "Nfib")),
  FeaturePlot(V1.neurons, features = c("Neurod2", "Neurod6", "Evx1", "Gata3")),
  nrow = 2
)
```

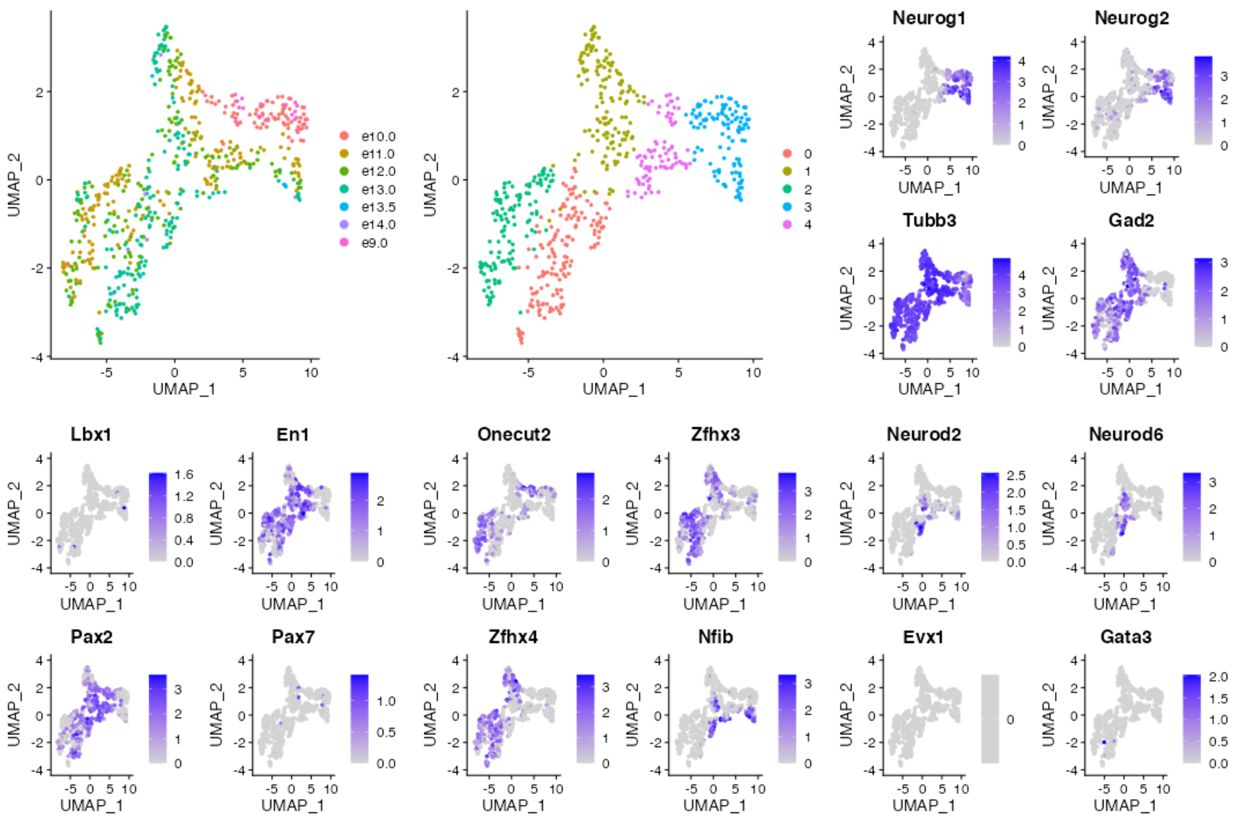

Pseudotemporal ordering using Slingshot.

```
sds <- slingshot(Embeddings(V1.neurons, "umap"),
  clusterLabels = V1.neurons$seurat_clusters,
  start.clus = 3, end.clus = c(0, 2), stretch = 0
)

cell_colors_clust <- cell_pal(V1.neurons$seurat_clusters, hue_pal())

plot.pseudotime.curves()
```

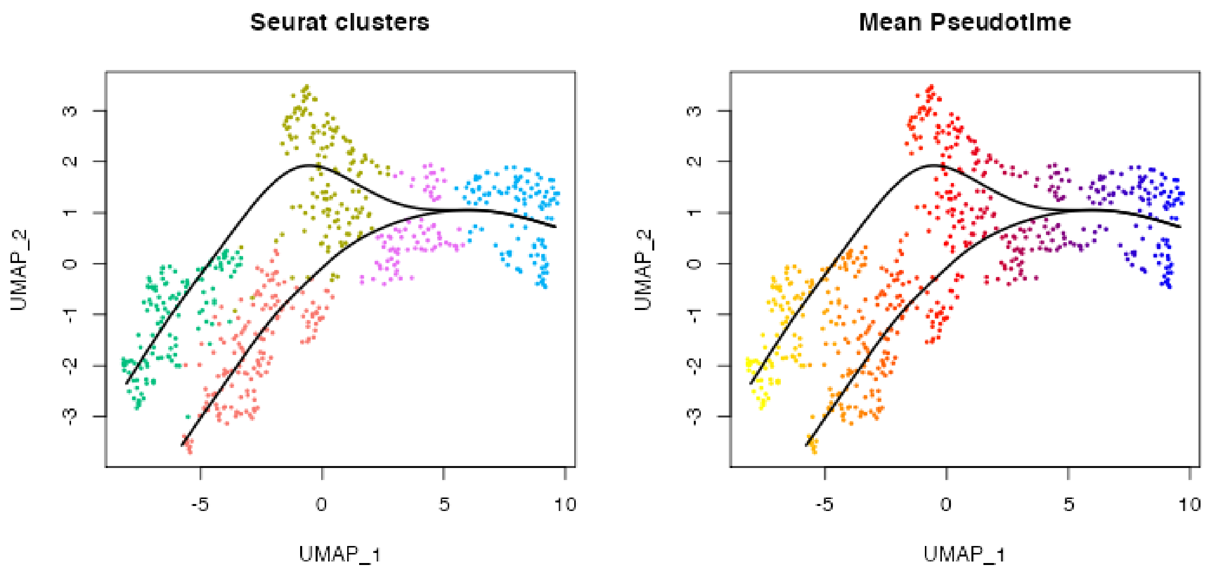

We exclude e9.0, e13.5 and e14.0 because there are very few neurons for this timepoint. we exclude curve3 because it crosses other curves.

Table 2: Number of cells / timepoint

e10.0	e11.0	e12.0	e13.0	e13.5	e14.0	e9.0
77	171	161	172	5	7	21

Plot pseudotime profiles of hindbrain V1 neurons

```
plot.gene.in.pseudotime.loom(
  gene = c(
    "Neurog2", "Tubb3", "Onecut2", "Zfhx3", "Zfhx4", "Nfia", "Nfib", "Neurod2", "Neurod6"
  ),
  pt.mtx = slingPseudotime(sds),
  seurat.object = V1.neurons,
  exclude.timepoint = c("e9.0", "e13.5", "e14.0"),
  exclude.curve = NULL,
  plot.title = "Hindbrain inhibitory neurons (V1)"
)
```

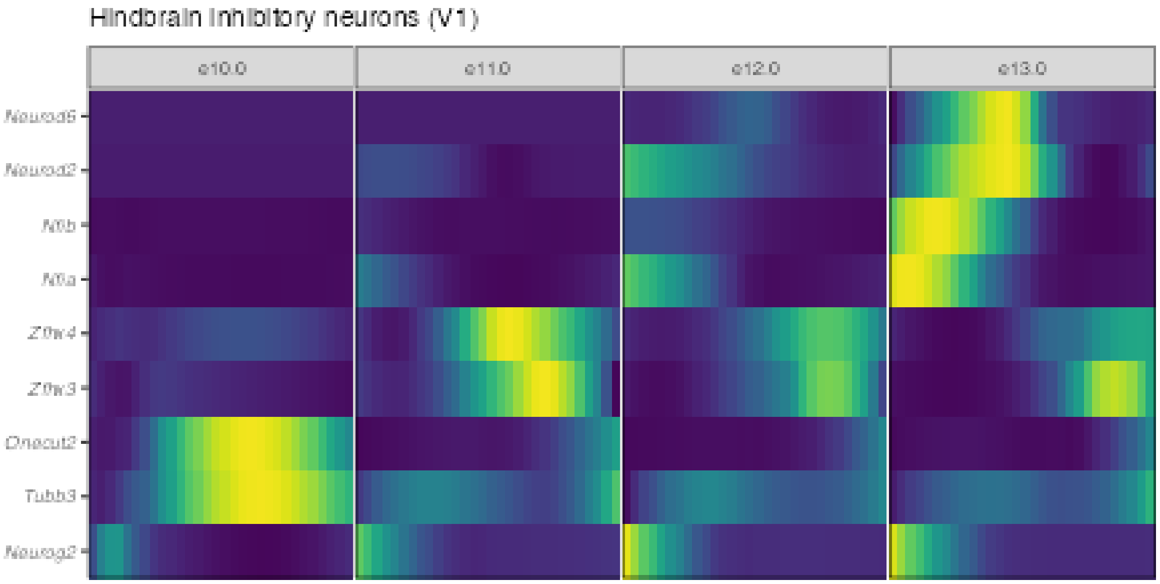


```r
plot.expression.dynamics.from.Seurat(
  input = V1.neurons,
  genes = c("Onecut2", "Pou2f2", "Zfhx3", "Zfhx4", "Nfia", "Nfib", "Neurod2", "Neurod6"),
  time = "age",
  title = "Hindbrain V1 neurons",
  from.loom = TRUE
)
```

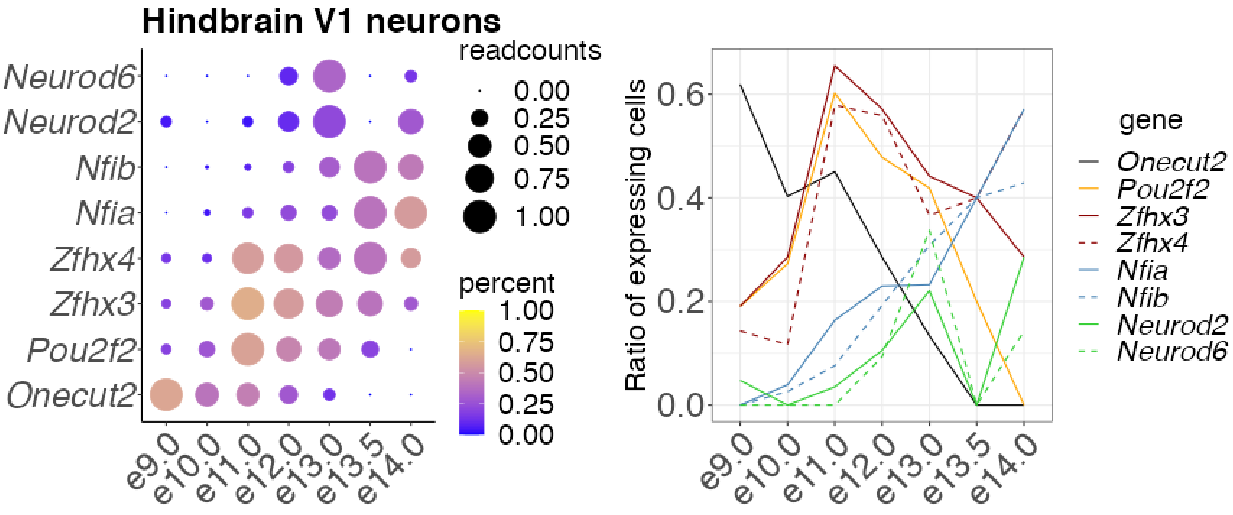

Pseudotemporal ordering of hindbrain dB4 neurons

We next focus on the Lbx1/Pax2-positive branch and perform subclustering. These neurons express Neurog1 during neurogenesis and thus might correspond to dB4/dI6 neurons although only very few neurons express Dmrt3.

```r
Lbx1.neurons <- subset(cr.neurons, subset = seurat_clusters %in% c(2, 4, 10)) %>%
  ScaleData(assay = "RNA") %>%
```

```
  FindVariableFeatures(selection.method = "vst", verbose = FALSE) %>%
  RunPCA(npcs = 30, verbose = FALSE) %>%
  RunUMAP(reduction = "pca", dims = 1:30) %>%
  FindNeighbors(dims = 1:30) %>%
  FindClusters(resolution = 1)

cowplot::plot_grid(DimPlot(Lbx1.neurons, reduction = "umap", group.by = "age"),
  DimPlot(Lbx1.neurons, reduction = "umap", group.by = "seurat_clusters"),
  FeaturePlot(Lbx1.neurons, features = c("Neurog1", "Neurog2", "Tubb3", "Gad2")),
  FeaturePlot(Lbx1.neurons, features = c("Lbx1", "En1", "Pax2", "Pax3")),
  FeaturePlot(Lbx1.neurons, features = c("Onecut2", "Zfhx3", "Zfhx4", "Nfib")),
  FeaturePlot(Lbx1.neurons, features = c("Neurod2", "Neurod6", "Dmrt3", "Bhlhe22")),
  nrow = 2
)
```

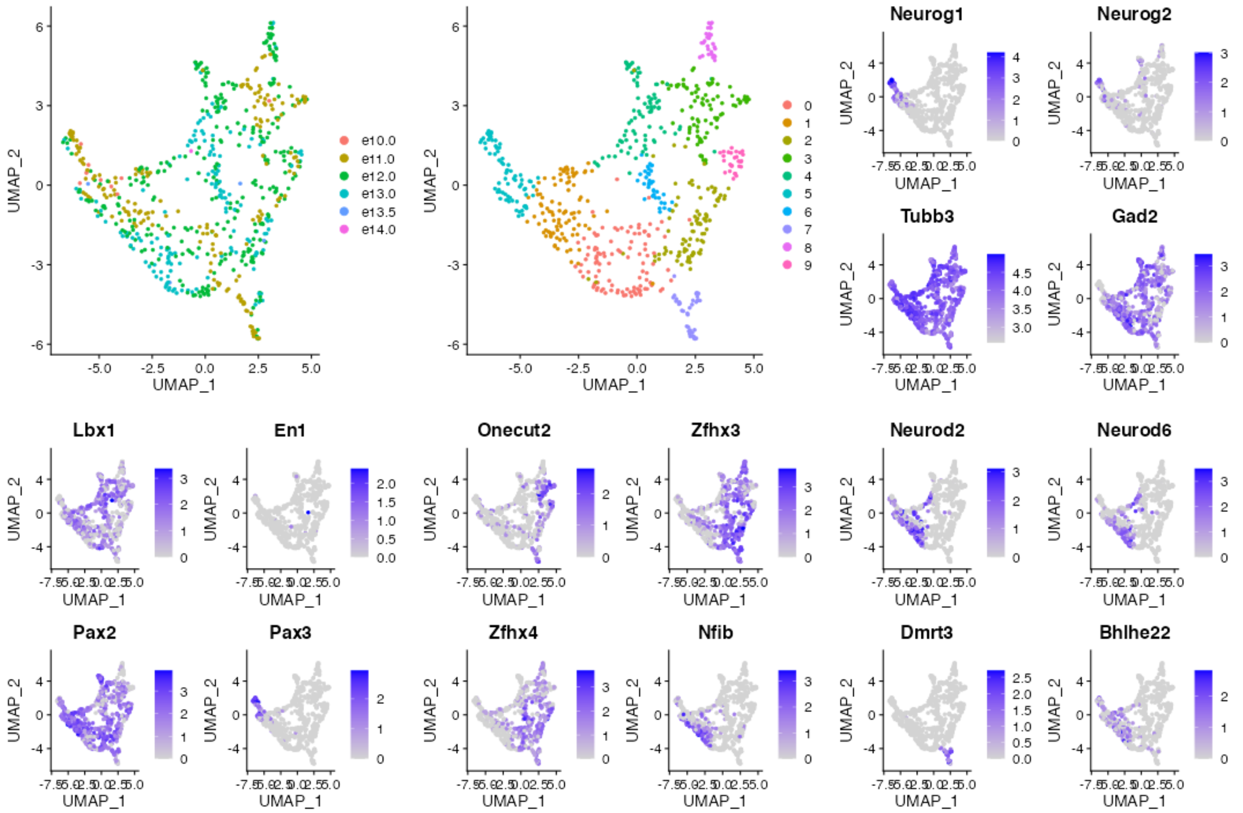

Pseudotemporal ordering using Slingshot.

```
sds <- slingshot(Embeddings(Lbx1.neurons, "umap"),
  clusterLabels = Lbx1.neurons$seurat_clusters,
  start.clus = 5, end.clus = c(8, 9), stretch = 0
)

cell_colors_clust <- cell_pal(Lbx1.neurons$seurat_clusters, hue_pal())

plot.pseudotime.curves()
```

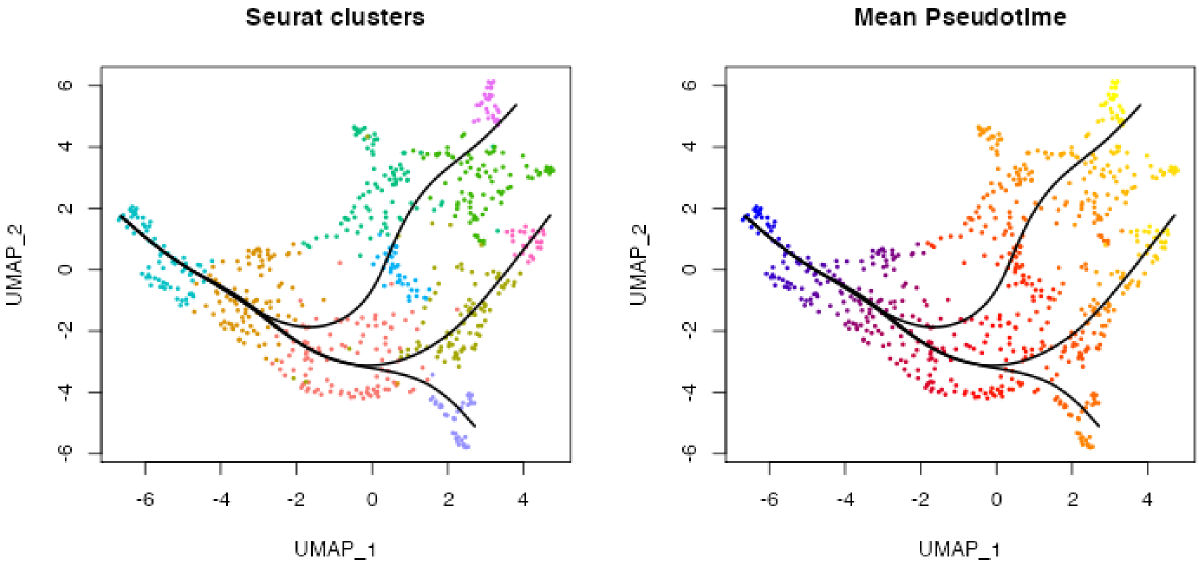

We exclude e10.0, e13.5 and e14.0 because there are very few neurons for this timepoint.

Table 3: Number of cells / timepoint

e10.0	e11.0	e12.0	e13.0	e13.5	e14.0
9	185	308	159	4	1

Plot pseudotime profiles of hindbrain dB4 neurons.

```
plot.gene.in.pseudotime.loom(
  gene = c(
    "Neurog1", "Tubb3", "Onecut2", "Zfhx3", "Zfhx4", "Nfia", "Nfib", "Neurod2", "Neurod6"
  ),
  pt.mtx = slingPseudotime(sds),
  seurat.object = Lbx1.neurons,
  exclude.timepoint = c("e10.0", "e13.5", "e14.0"),
  exclude.curve = NULL,
  plot.title = "Hindbrain dB4 neurons"
)
```

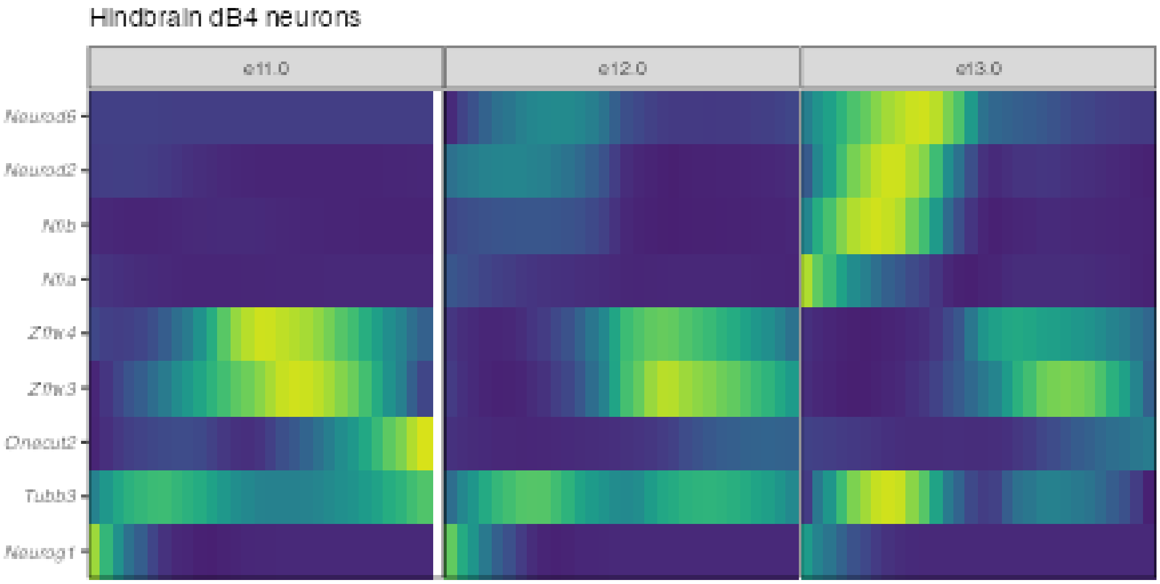


```
plot.expression.dynamics.from.Seurat(
  input = Lbx1.neurons,
  genes = c("Onecut2", "Pou2f2", "Zfhx3", "Zfhx4", "Nfia", "Nfib", "Neurod2", "Neurod6"),
  time = "age",
  title = "Hindbrain dB4 neurons",
  from.loom = TRUE
)
```

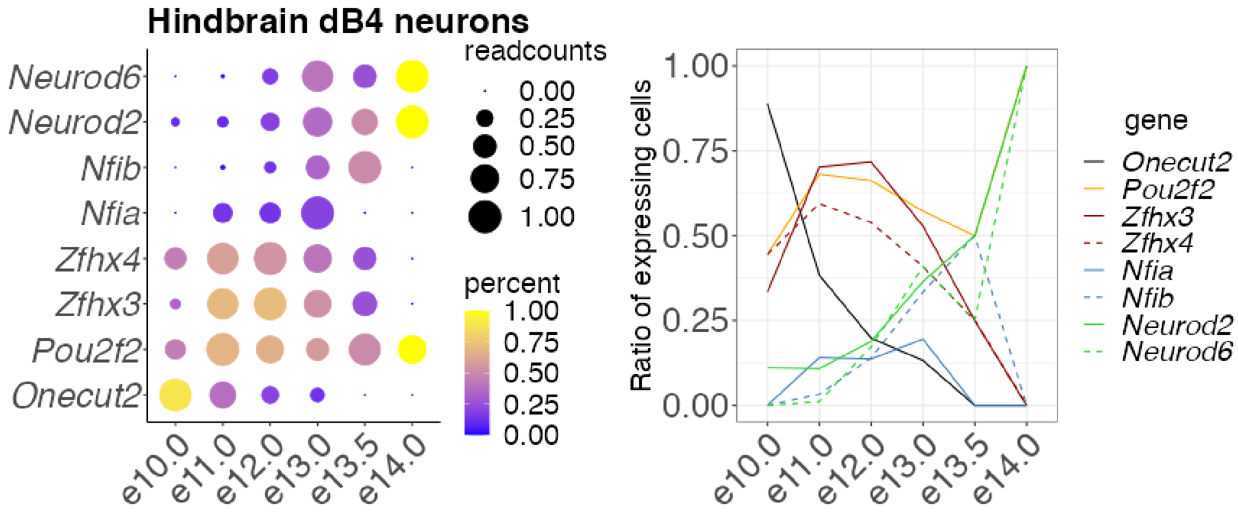

Pseudotemporal gene expression analysis of dA1 neurons (Lhx2+/Lhx9+)

```
cr.neurons <- hb.seurat %>%
  subset(subset = seurat_clusters %in% c(3, 11, 14)) %>%
  ScaleData(assay = "RNA") %>%
  FindVariableFeatures(selection.method = "vst", verbose = FALSE) %>%
  RunPCA(npcs = 30, verbose = FALSE) %>%
```

```
  RunUMAP(reduction = "pca", dims = 1:30) %>%
  FindNeighbors(dims = 1:30) %>%
  FindClusters(resolution = 0.5)

cowplot::plot_grid(DimPlot(cr.neurons, reduction = "umap", group.by = "age"),
  DimPlot(cr.neurons, reduction = "umap", group.by = "seurat_clusters"),
  FeaturePlot(cr.neurons, features = c("Atoh1", "Lhx2", "Lhx9", "Onecut2")),
  FeaturePlot(cr.neurons, features = c("Zfhx3", "Zfhx4", "Nfia", "Nfib")),
  FeaturePlot(cr.neurons, features = c("Neurod2", "Neurod6", "Hoxb4", "Hoxb5")),
  FeaturePlot(cr.neurons, features = c("Barhl1", "Barhl2", "Pax6", "Tubb3")),
  nrow = 2
)
```

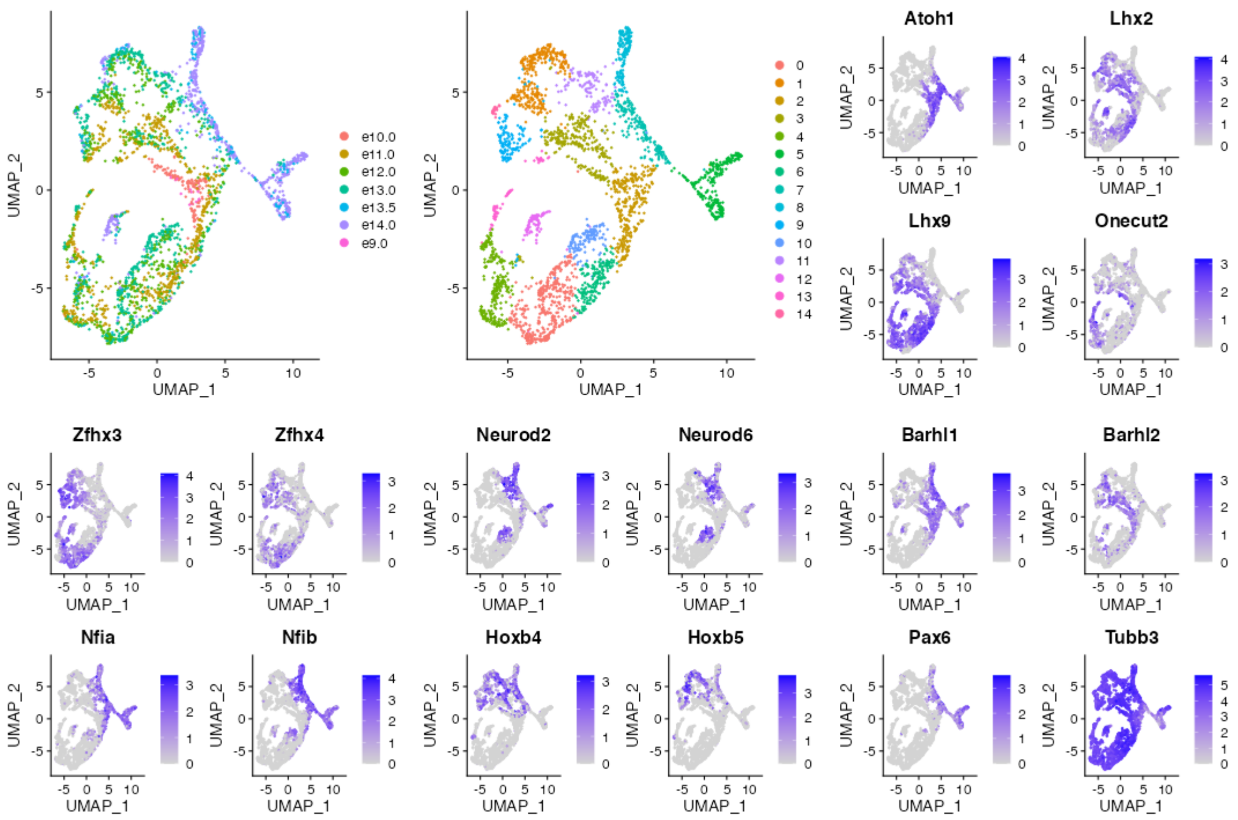

We exclude clusters 5, 7 and 8 as these are Lhx2/9 negative. These neurons instead express Pax6 and Barhl1 and thus may be precerebellar neurons (Engelkamp et al., Development, 1999, Li et al., J Neurosci, 2004). Of note, they are also strongly positive for Nfia/b and Neurod2/6.

```
cr.neurons <- cr.neurons %>%
  subset(subset = seurat_clusters %!in% c(5, 7, 8)) %>%
  ScaleData(assay = "RNA") %>%
  FindVariableFeatures(selection.method = "vst", verbose = FALSE) %>%
  RunPCA(npcs = 30, verbose = FALSE) %>%
  RunUMAP(reduction = "pca", dims = 1:30) %>%
  FindNeighbors(dims = 1:30) %>%
  FindClusters(resolution = 0.5)

cowplot::plot_grid(DimPlot(cr.neurons, reduction = "umap", group.by = "age"),
```

```
  DimPlot(cr.neurons, reduction = "umap", group.by = "seurat_clusters"),
  FeaturePlot(cr.neurons, features = c("Atoh1", "Lhx2", "Lhx9", "Onecut2")),
  FeaturePlot(cr.neurons, features = c("Zfhx3", "Zfhx4", "Nfia", "Nfib")),
  FeaturePlot(cr.neurons, features = c("Neurod2", "Neurod6", "Hoxb4", "Hoxb5")),
  FeaturePlot(cr.neurons, features = c("Shox2", "Tfap2d", "Otx2", "Sox7")),
  nrow = 2
)
```

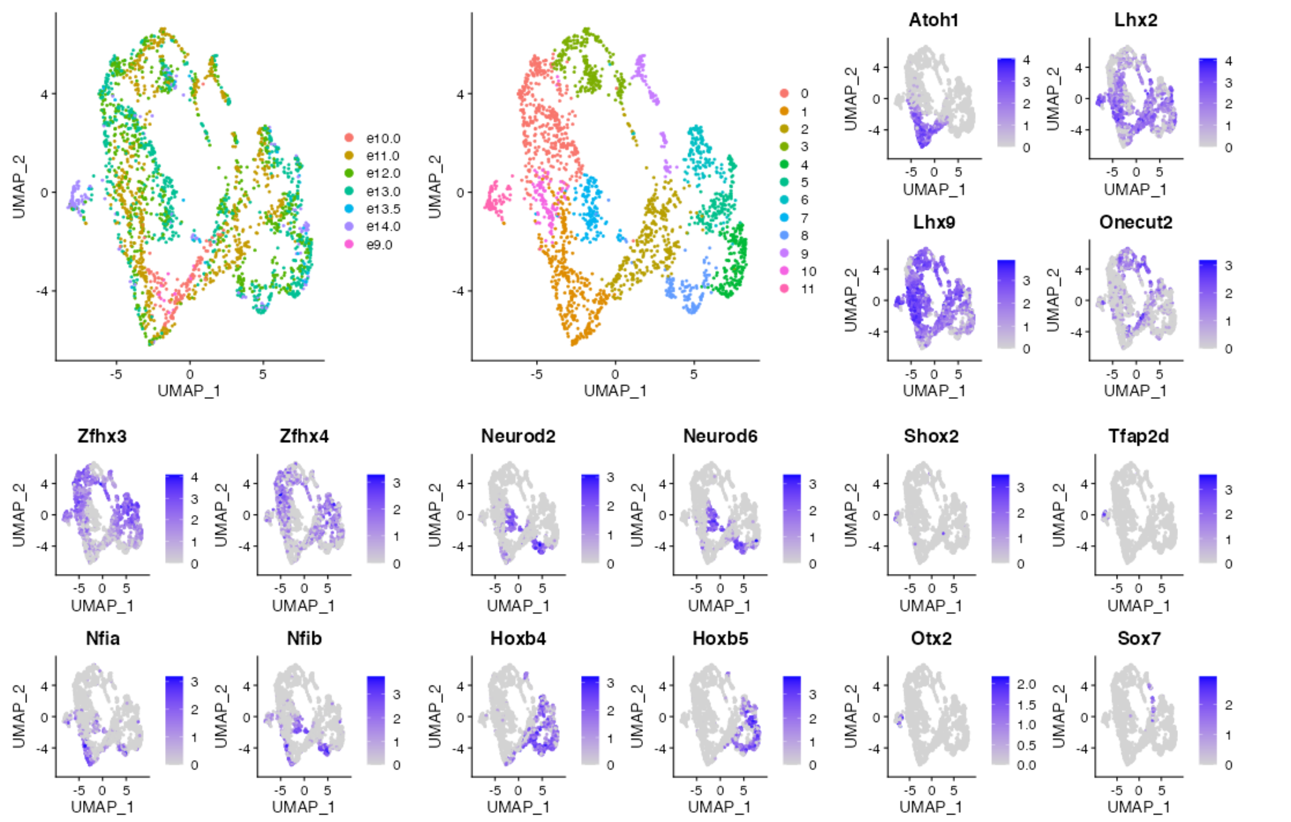

These neurons can be subdivided on Hox-positive and Hox-negative neurons. We focus on the Hox-negative population for further analysis. We also exclude cluster 11 because these cells are Otx2, Shox2 and Tfap2d-positive and thus may be midbrain (Manno et al. Cell 2016). We also exclude cluster 9 because it expresses high levels of Sox7, which has previously been shown to be pro-apoptotic.

```
dA1.neurons <- cr.neurons %>%
  subset(subset = seurat_clusters %in% c(0, 1, 3, 7, 10)) %>%
  ScaleData(assay = "RNA") %>%
  FindVariableFeatures(selection.method = "vst", verbose = FALSE) %>%
  RunPCA(npcs = 30, verbose = FALSE) %>%
  RunUMAP(reduction = "pca", dims = 1:30) %>%
  FindNeighbors(dims = 1:30) %>%
  FindClusters(resolution = 0.5)

cowplot::plot_grid(DimPlot(dA1.neurons, reduction = "umap", group.by = "age"),
  DimPlot(dA1.neurons, reduction = "umap", group.by = "seurat_clusters"),
  FeaturePlot(dA1.neurons, features = c("Atoh1", "Lhx2", "Lhx9", "Onecut2")),
  FeaturePlot(dA1.neurons, features = c("Zfhx3", "Zfhx4", "Nfia", "Nfib")),
  FeaturePlot(dA1.neurons, features = c("Neurod2", "Neurod6", "Hoxb4", "Hoxb5")),
```

```r
  FeaturePlot(dA1.neurons, features = c("Shox2", "Tfap2d", "Otx2", "Sox7")),
  nrow = 2
)
```

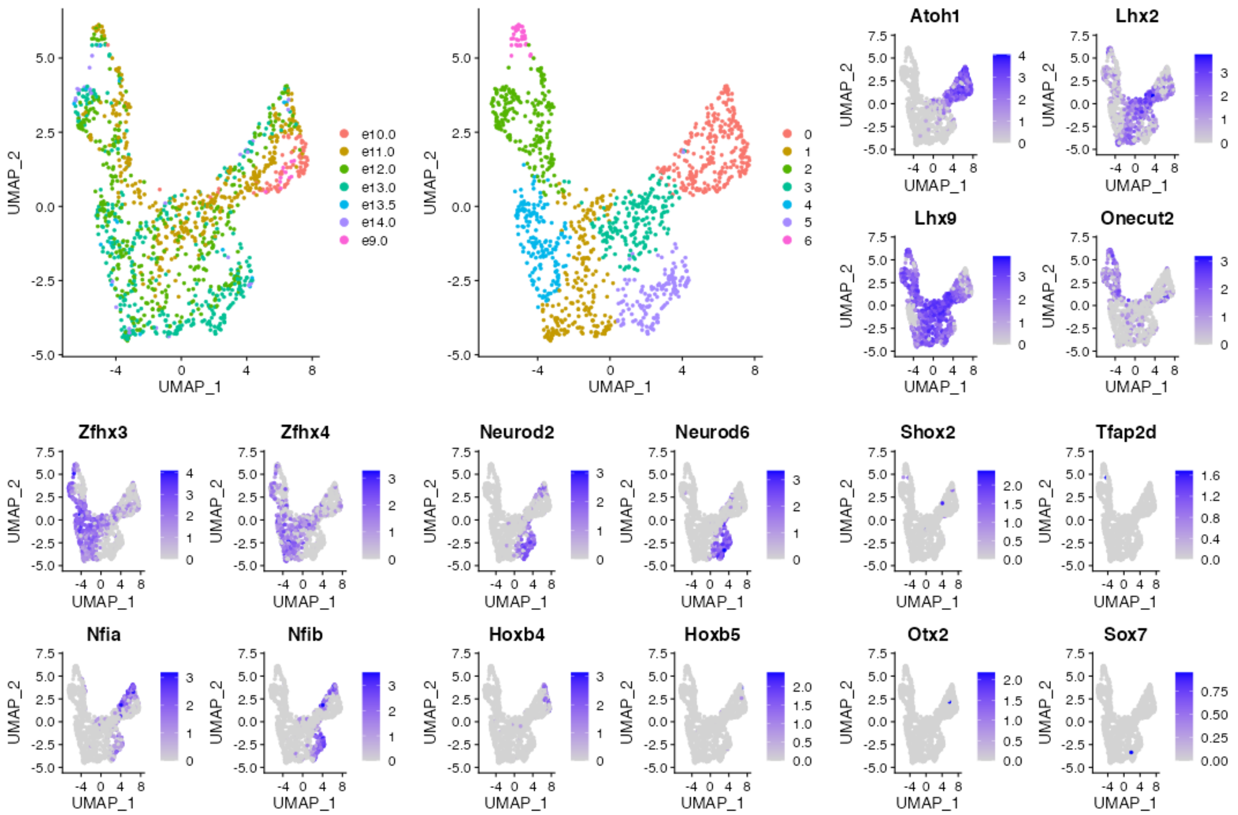

Pseudotemporal ordering using Slingshot.

```r
sds <- slingshot(Embeddings(dA1.neurons, "umap"),
  clusterLabels = dA1.neurons$seurat_clusters,
  start.clus = 0, end.clus = 6, stretch = 0
)

cell_colors_clust <- cell_pal(dA1.neurons$seurat_clusters, hue_pal())

plot.pseudotime.curves()
```

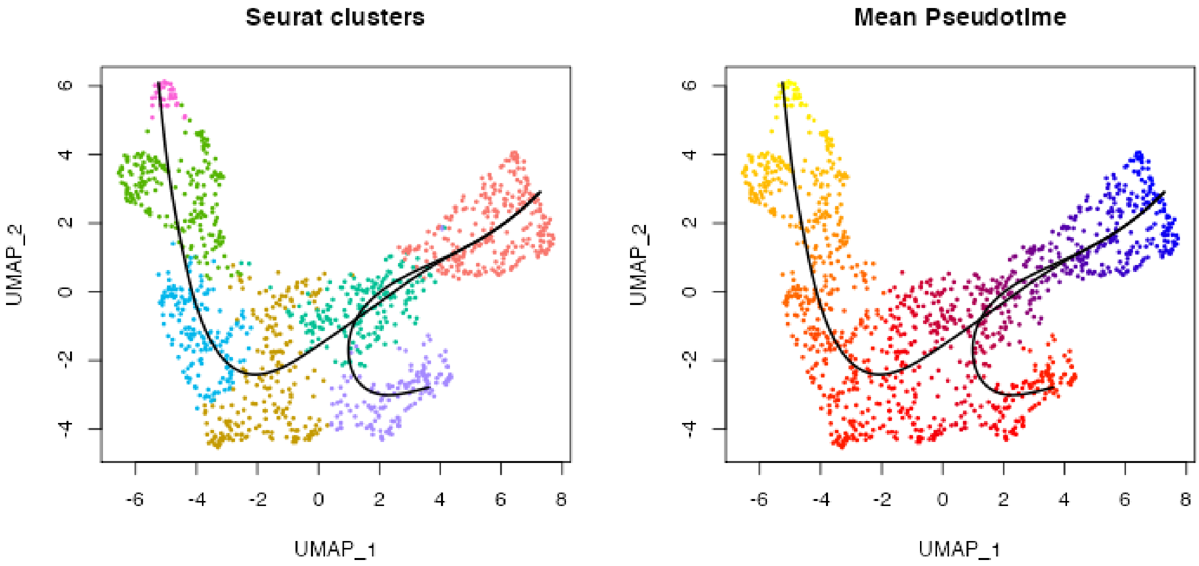

We exclude e9.0 and e14.0 because there are very few neurons for these timepoints.

Table 4: Number of cells / timepoint

e10.0	e11.0	e12.0	e13.0	e13.5	e14.0	e9.0
79	334	387	364	18	32	11

Plot pseudotime profiles of hindbrain Hox-negative dA1 neurons.

```
plot.gene.in.pseudotime.loom(
  gene = c(
    "Atoh1", "Tubb3", "Onecut2", "Zfhx3", "Zfhx4", "Nfia", "Nfib", "Neurod2", "Neurod6"
  ),
  pt.mtx = slingPseudotime(sds),
  seurat.object = dA1.neurons,
  exclude.timepoint = c("e9.0", "e14.0"),
  exclude.curve = NULL,
  plot.title = "Hindbrain dA1 neurons (Hox-negative)"
)
```

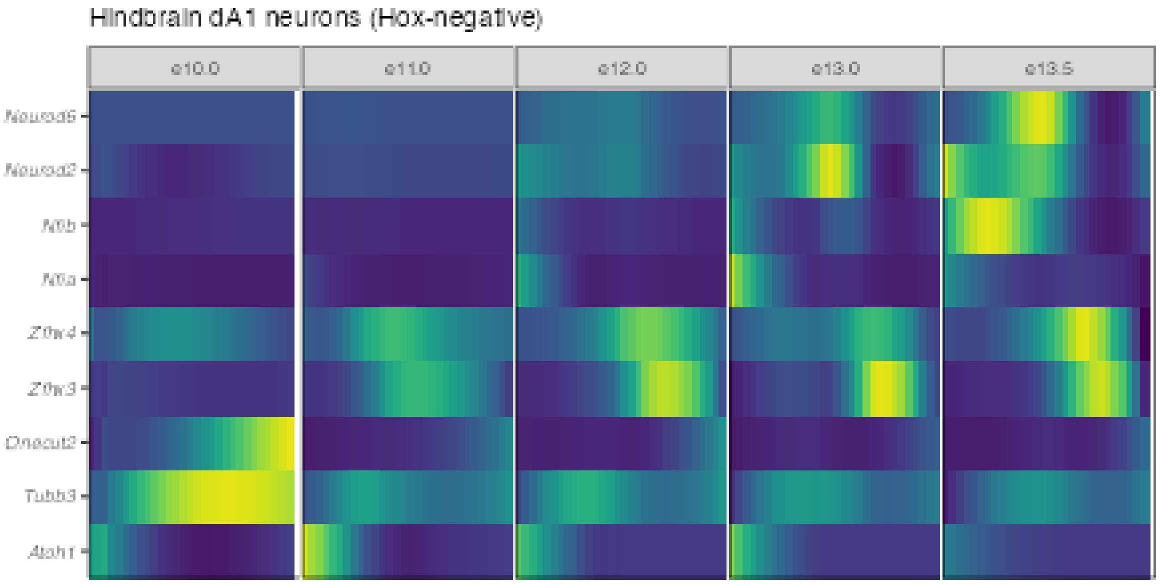


```
plot.expression.dynamics.from.Seurat(
  input = dA1.neurons,
  genes = c("Onecut2", "Pou2f2", "Zfhx3", "Zfhx4", "Nfia", "Nfib", "Neurod2", "Neurod6"),
  time = "age",
  title = "Hindbrain dA1 neurons (Hox-negative)",
  from.loom = TRUE
)
```

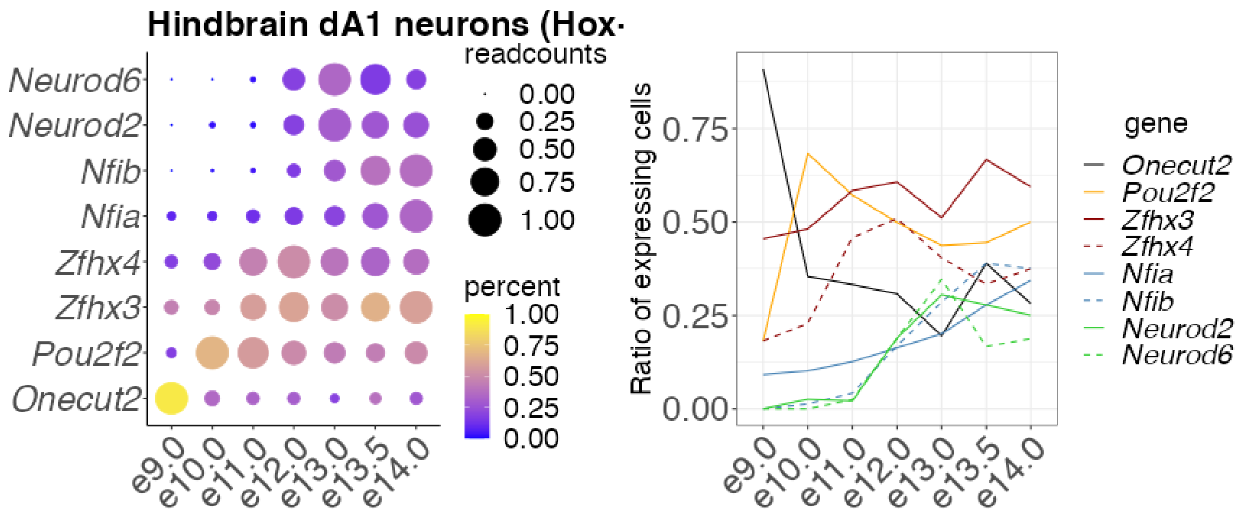

Load forebrain scRNAseq data from La Manno et al. 2020

We subset the data to annotated forebrain neurons and convert the data in a Seurat object.

```
### Generate Forebrain-specific Seurat object

tissue <- "Forebrain"
celltype <- "Neuron"
```

```r
timepoints <- c("e9.0", "e10.0", "e11.0", "e12.0", "e12.5", "e13.0", "e13.5", "e14.0")

tissue.id <- which(grepl(tissue, unique(sc.loom$col.attrs$Tissue[])) == TRUE)
cell.id <- intersect(
  which(sc.meta$tissue %in% unique(sc.loom$col.attrs$Tissue[])[tissue.id] &
    sc.meta$class == celltype),
  which(sc.meta$age %in% timepoints)
)

exp.mat <- sc.loom[["matrix"]][cell.id, ]

colnames(exp.mat) <- sc.loom$row.attrs$Gene[]
rownames(exp.mat) <- sc.meta$cellID[cell.id]

exc.seurat <- CreateSeuratObject(
  counts = t(exp.mat),
  meta.data = sc.meta[cell.id, ] %>%
    as.tibble() %>%
    tibble::column_to_rownames("cellID")
)

exc.seurat[["percent.mt"]] <- PercentageFeatureSet(exc.seurat, pattern = "^mt-")

exc.seurat <- exc.seurat %>%
  subset(subset = nFeature_RNA > 600 & nFeature_RNA < 6000 & percent.mt < 6) %>%
  SCTransform(vars.to.regress = "sampleID") %>%
  NormalizeData(verbose = FALSE, assay = "SCT") %>%
  ScaleData(verbose = FALSE, assay = "SCT") %>%
  FindVariableFeatures(selection.method = "vst", verbose = FALSE) %>%
  RunPCA(npcs = 30, verbose = FALSE) %>%
  RunUMAP(reduction = "pca", dims = 1:30) %>%
  FindNeighbors(dims = 1:30) %>%
  FindClusters(resolution = 0.5)

cowplot::plot_grid(DimPlot(exc.seurat, reduction = "umap", group.by = "age"),
  DimPlot(exc.seurat, reduction = "umap", group.by = "seurat_clusters"),
  FeaturePlot(exc.seurat, features = c("Neurog2", "Ascl1", "Gad2", "Slc17a6")),
  FeaturePlot(exc.seurat, features = c("Onecut2", "Zfhx3", "Zfhx4", "Nfia")),
  FeaturePlot(exc.seurat, features = c("Nfib", "Neurod2", "Neurod6", "Reln")),
  FeaturePlot(exc.seurat, features = c("Lhx6", "Meis2", "Foxg1", "Isl1")),
  nrow = 2
)
```

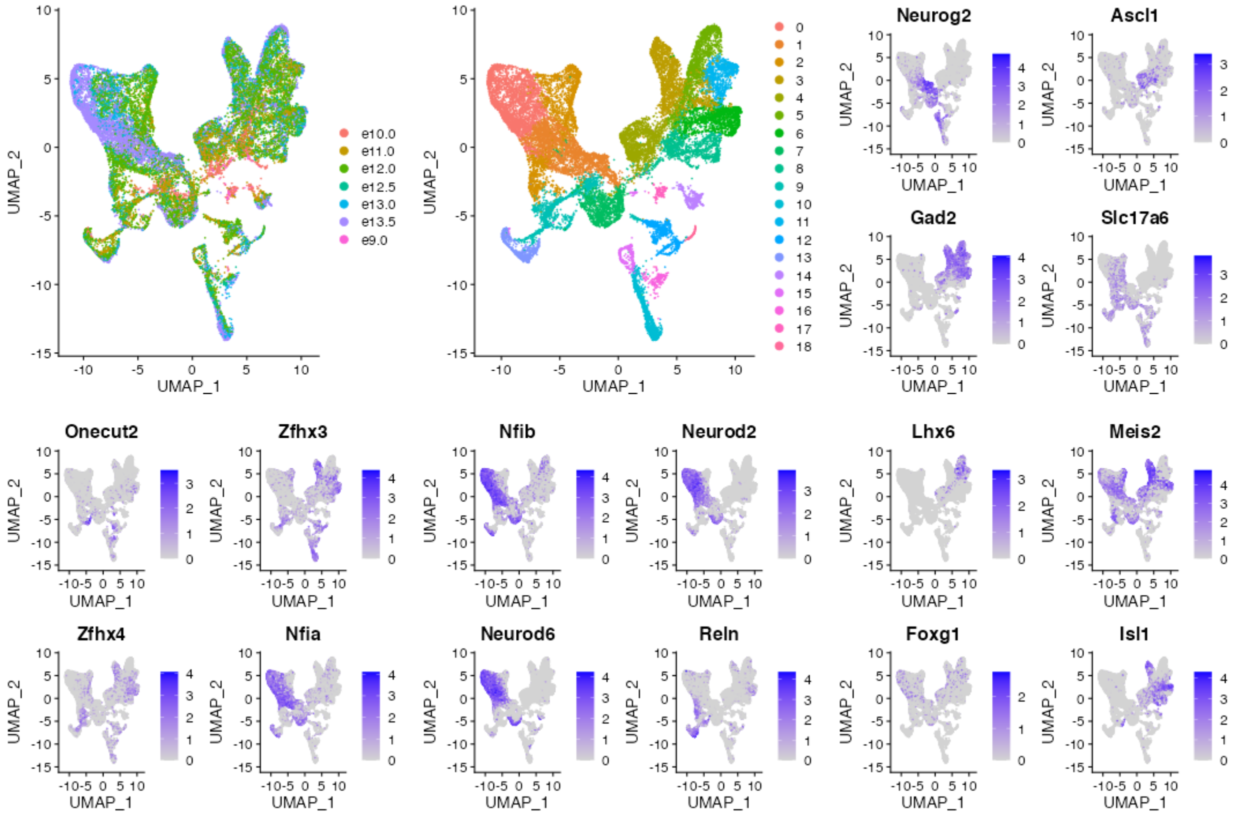


```
fb <- plot.expression.dynamics.from.Seurat(
  input = exc.seurat,
  genes = c("Onecut2", "Pou2f2", "Zfhx3", "Zfhx4", "Nfia", "Nfib", "Neurod2", "Neurod6"),
  time = "age",
  title = "Forebrain",
  from.loom = TRUE,
  colors = c("black", "orange", "darkred", "darkred", "steelblue", "steelblue", "limegreen", "limegreen
  linetype = c("solid", "solid", "solid", "dashed", "solid", "dashed", "solid", "dashed")
)

fb
```

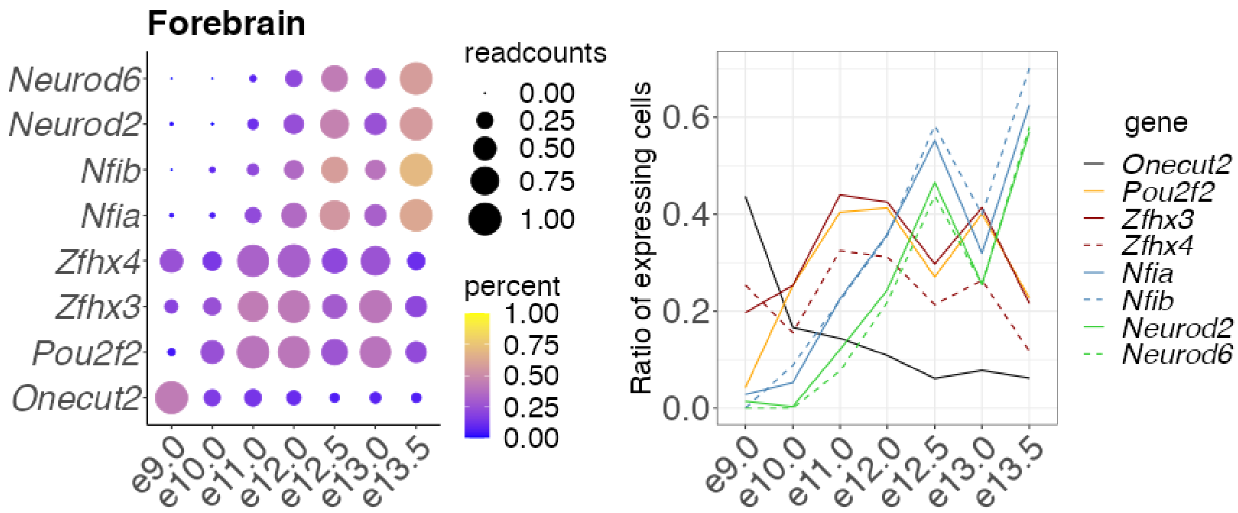

Pseudotemporal gene expression analysis of cortical excitatory neurons

Subset data to excitatory neurons.

```
cr.neurons <- exc.seurat %>%
  subset(subset = seurat_clusters %in% c(0, 1, 2)) %>%
  NormalizeData(assay = "RNA") %>%
  ScaleData(assay = "RNA") %>%
  FindVariableFeatures(selection.method = "vst", verbose = FALSE) %>%
  RunPCA(npcs = 30, verbose = FALSE) %>%
  RunUMAP(reduction = "pca", dims = 1:30) %>%
  FindNeighbors(dims = 1:30) %>%
  FindClusters(resolution = 0.5)

cowplot::plot_grid(DimPlot(cr.neurons, reduction = "umap", group.by = "age"),
  DimPlot(cr.neurons, reduction = "umap", group.by = "seurat_clusters"),
  FeaturePlot(cr.neurons, features = c("Neurog2", "Tubb3", "Onecut2", "Zfhx3")),
  FeaturePlot(cr.neurons, features = c("Zfhx4", "Nfia", "Nfib", "Neurod2")),
  ncol = 2
)
```

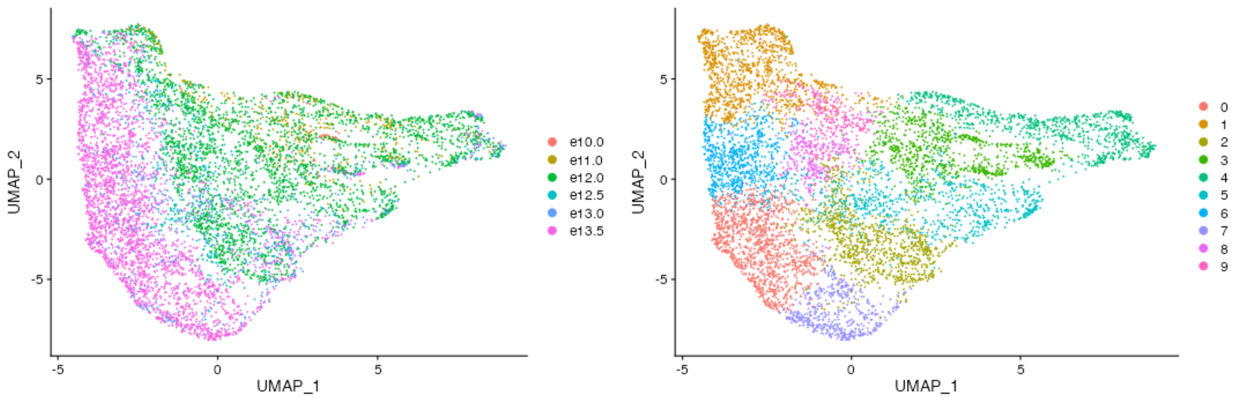

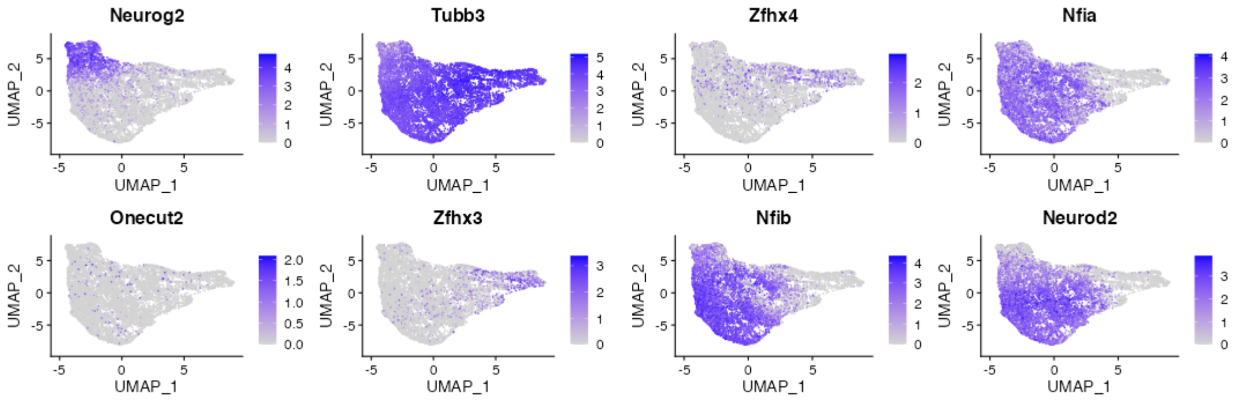

Pseudotemporal ordering using Slingshot. We exclude curve3 because it folds back and crosses curve1.

```
sds <- slingshot(Embeddings(cr.neurons, "umap"),
  clusterLabels = cr.neurons$seurat_clusters,
  start.clus = 1, end.clus = c(2, 4, 7), stretch = 0
)

cell_colors_clust <- cell_pal(cr.neurons$seurat_clusters, hue_pal())

plot.pseudotime.curves(exclude.curve = "curve3")
```

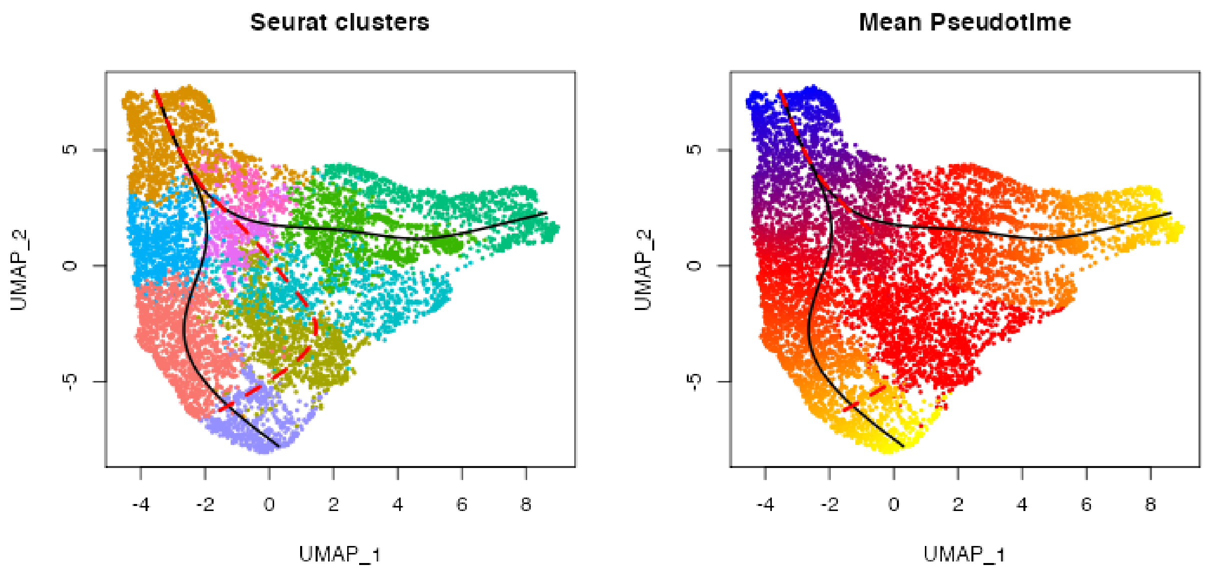

Plot pseudotime profiles of cortical excitatory neurons

```
plot.gene.in.pseudotime.loom(
  gene = c(
    "Neurog2", "Tubb3", "Onecut2", "Zfhx3", "Zfhx4", "Nfia", "Nfib", "Neurod2", "Neurod6"
  ),
  pt.mtx = slingPseudotime(sds),
  seurat.object = cr.neurons,
  exclude.timepoint = "e10.0",
  exclude.curve = NULL,
  plot.title = "Forebrain excitatory neurons"
)
```

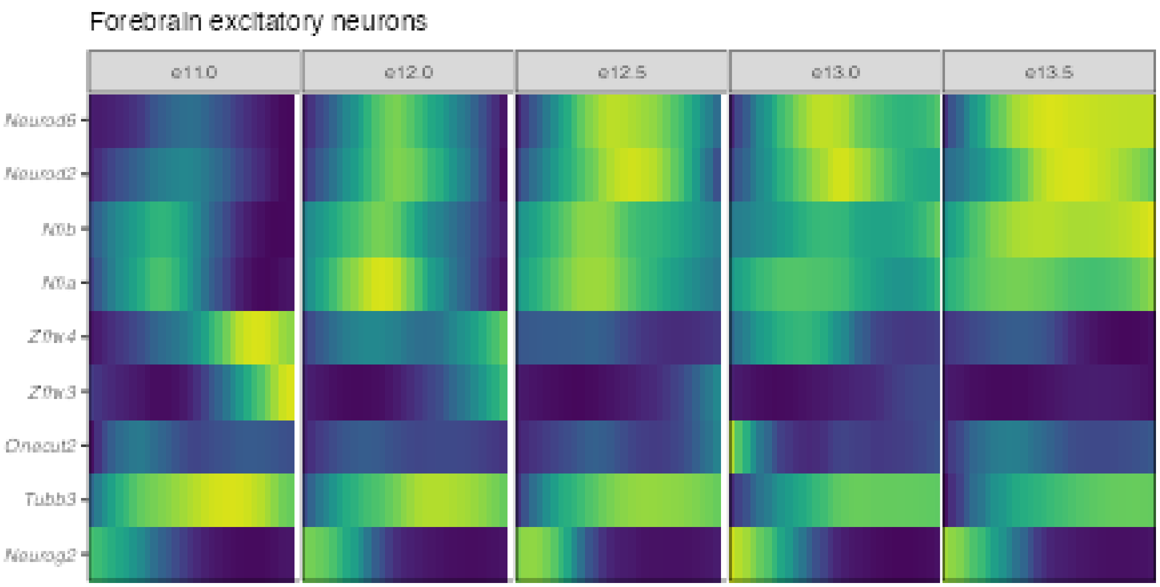

```r
plot.expression.dynamics.from.Seurat(
  input = cr.neurons,
  genes = c("Onecut2", "Pou2f2", "Zfhx3", "Zfhx4", "Nfia", "Nfib", "Neurod2", "Neurod6"),
  time = "age",
  title = "Forebrain excitatory neurons",
  from.loom = TRUE
)
```

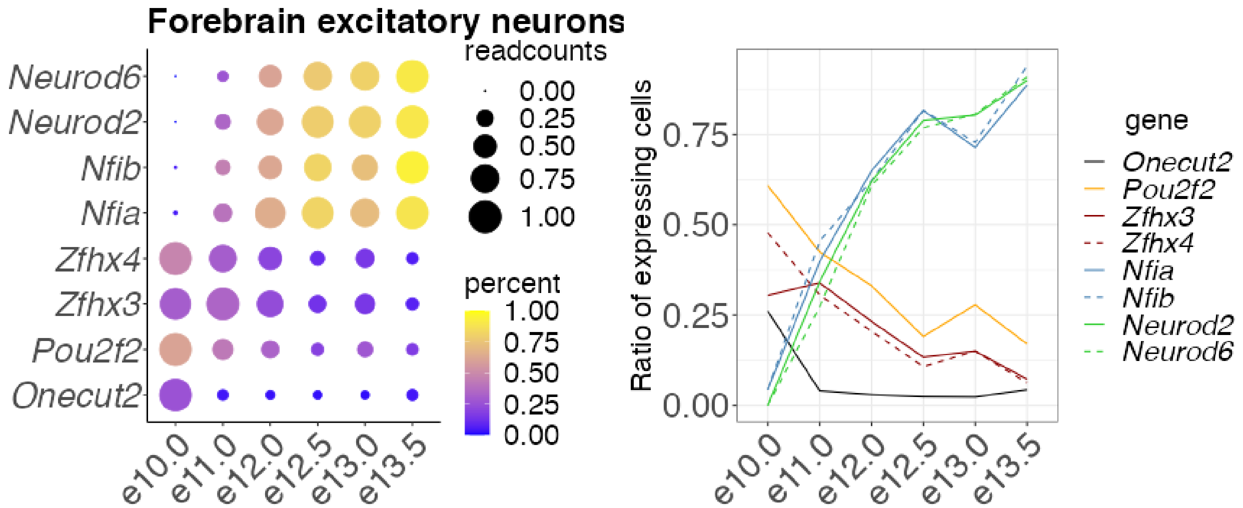

Pseudotemporal gene expression analysis of forebrain inhibitory neurons

Subset data to inhibitory neurons.

```r
cr.neurons <- exc.seurat %>%
  subset(subset = seurat_clusters %in% c(3, 4, 5, 6, 8, 11)) %>%
  NormalizeData(assay = "RNA") %>%
  ScaleData(assay = "RNA") %>%
  FindVariableFeatures(selection.method = "vst", verbose = FALSE) %>%
  RunPCA(npcs = 30, verbose = FALSE) %>%
  RunUMAP(reduction = "pca", dims = 1:30) %>%
  FindNeighbors(dims = 1:30) %>%
  FindClusters(resolution = 0.5)

cowplot::plot_grid(DimPlot(cr.neurons, reduction = "umap", group.by = "age"),
  DimPlot(cr.neurons, reduction = "umap", group.by = "seurat_clusters"),
  FeaturePlot(cr.neurons, features = c("Ascl1", "Gad2", "Tubb3", "Onecut2")),
  FeaturePlot(cr.neurons, features = c("Zfhx3", "Zfhx4", "Nfia", "Nfib")),
  FeaturePlot(cr.neurons, features = c("Ebf1", "Meis2", "Lhx6", "Isl1")),
  FeaturePlot(cr.neurons, features = c("Pax6", "Satb1", "Sox6", "Hmx3")),
  nrow = 2
)
```

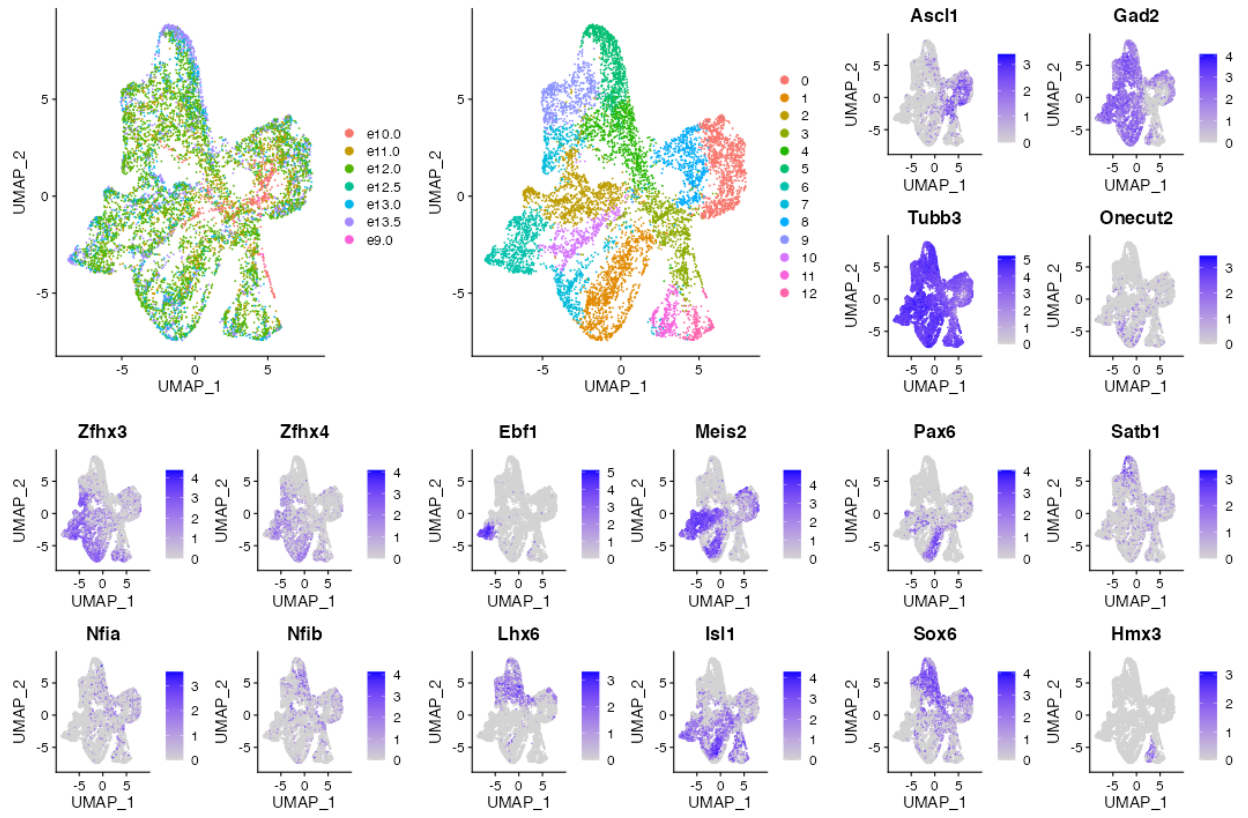

Pseudotemporal ordering using Slingshot. We exclude curve1 because its trajectory crosses multiple other trajectories.

```
sds <- slingshot(Embeddings(cr.neurons, "umap"),
  clusterLabels = cr.neurons$seurat_clusters,
  start.clus = 0, end.clus = c(11, 1, 6, 5), stretch = 0
)

cell_colors_clust <- cell_pal(cr.neurons$seurat_clusters, hue_pal())

plot.pseudotime.curves(exclude.curve = "curve1")
```

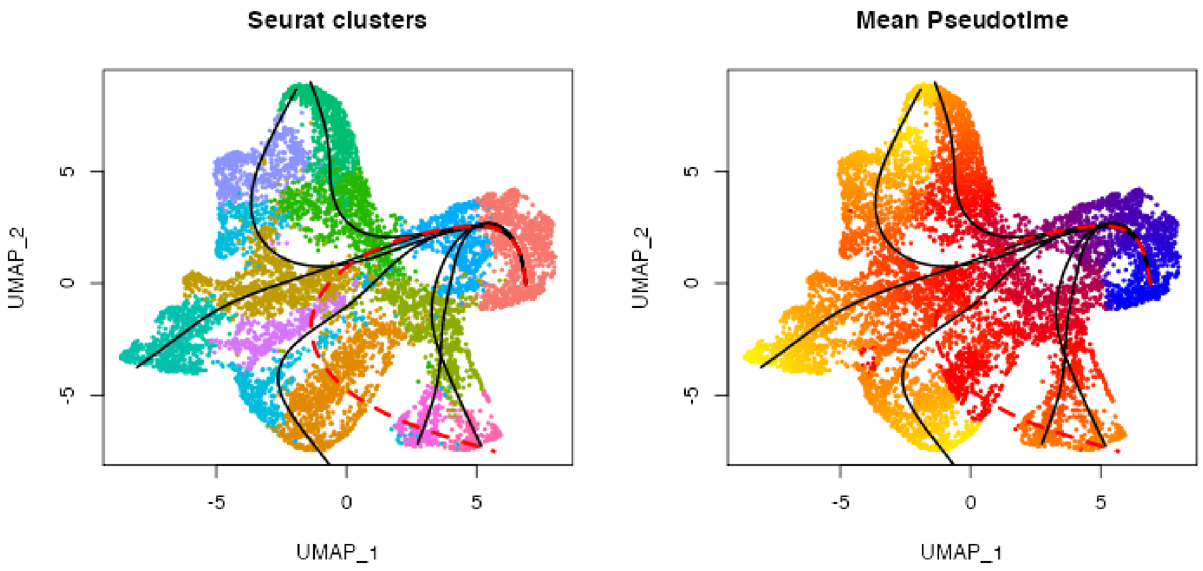

Plot pseudotime profiles of forebrain inhibitory neurons

We do not plot Neurod2 and Neurod6 because forebrain inhibitory neurons do not express these markers.

```
plot.gene.in.pseudotime.loom(
  gene = c("Ascl1", "Tubb3", "Onecut2", "Zfhx3", "Zfhx4", "Nfia", "Nfib"),
  pt.mtx = slingPseudotime(sds),
  seurat.object = cr.neurons,
  exclude.timepoint = "e9.0",
  exclude.curve = c("curve1"),
  plot.title = "Forebrain Inhibitory Neurons"
)
```

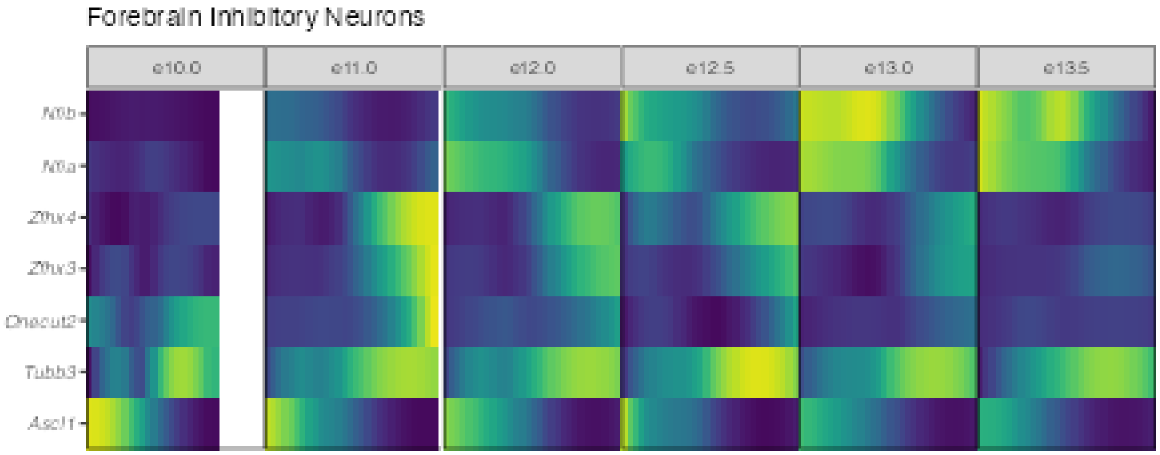


```
plot.expression.dynamics.from.Seurat(
  input = cr.neurons,
  genes = c("Onecut2", "Pou2f2", "Zfhx3", "Zfhx4", "Nfia", "Nfib"),
  time = "age",
  title = "Forebrain inhibitory neurons",
```

```
  from.loom = TRUE
)
```

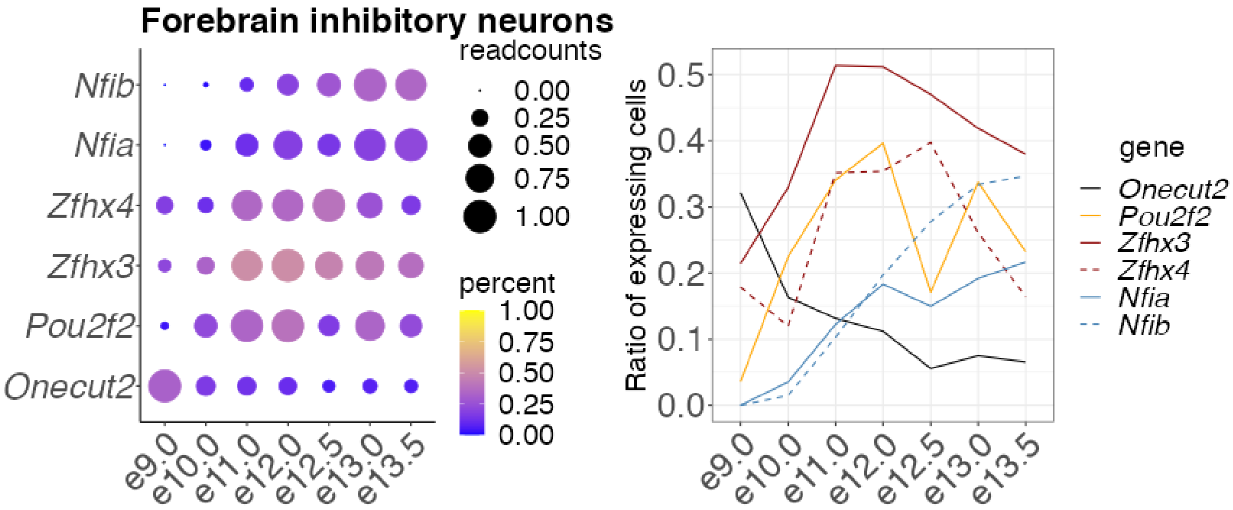

Pseudotemporal gene expression analysis of MGE neurons

We further partition the neurons into distinct neuronal subsets. We start by subsetting out the Lhx6-positive neurons, which probably correspond to MGE neurons.

```
Lhx6.neurons <- cr.neurons %>%
  subset(subset = seurat_clusters %in% c(4, 5, 7, 9)) %>%
  FindVariableFeatures(selection.method = "vst", verbose = FALSE) %>%
  RunPCA(npcs = 30, verbose = FALSE) %>%
  RunUMAP(reduction = "pca", dims = 1:30) %>%
  FindNeighbors(dims = 1:30) %>%
  FindClusters(resolution = 0.5)

cowplot::plot_grid(DimPlot(Lhx6.neurons, reduction = "umap", group.by = "age"),
  DimPlot(Lhx6.neurons, reduction = "umap", group.by = "seurat_clusters"),
  FeaturePlot(Lhx6.neurons, features = c("Ascl1", "Gad2", "Tubb3", "Onecut2")),
  FeaturePlot(Lhx6.neurons, features = c("Zfhx3", "Zfhx4", "Nfia", "Nfib")),
  FeaturePlot(Lhx6.neurons, features = c("Ebf1", "Meis2", "Lhx6", "Sox6")),
  FeaturePlot(Lhx6.neurons, features = c("Pax6", "Satb1", "Sox14", "Hmx3")),
  nrow = 2
)
```

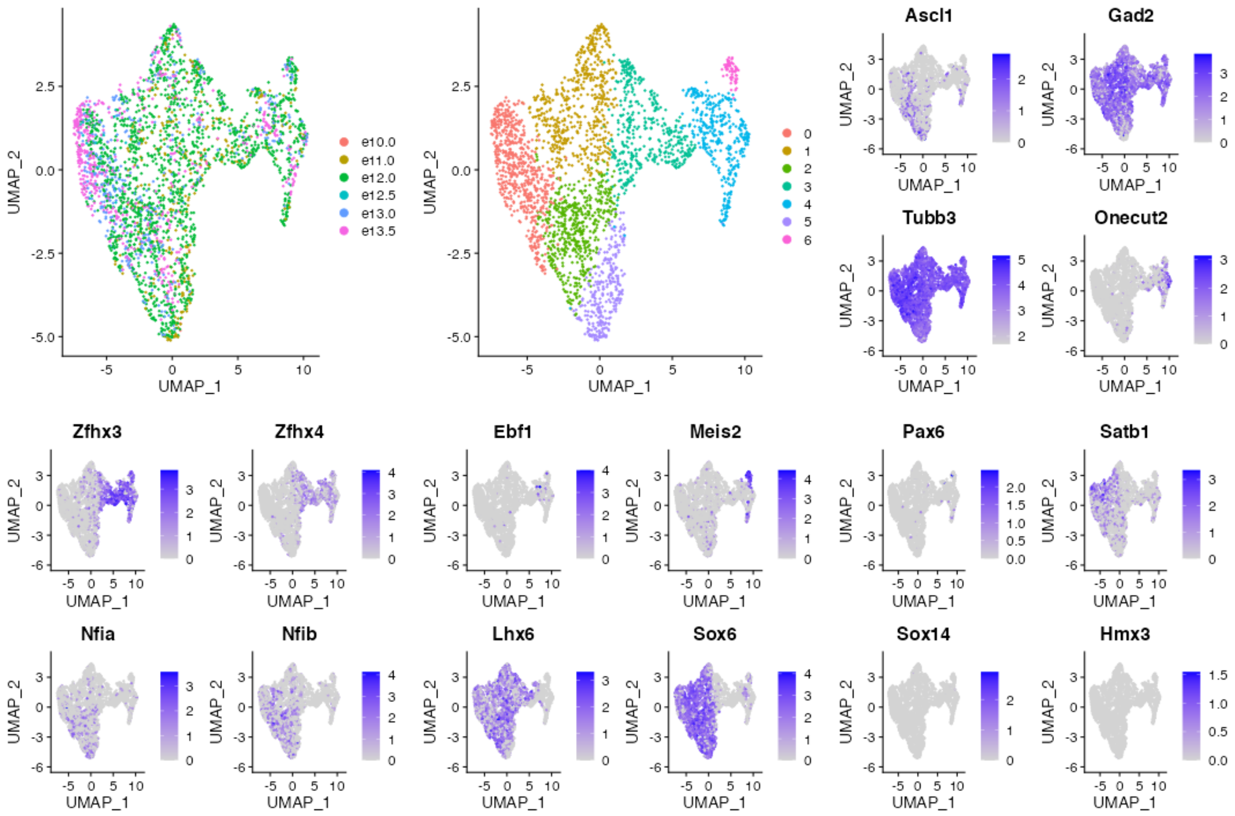

We remove clusters 4 and 6 because they are Lhx6-negative.

```
Lhx6.neurons <- subset(Lhx6.neurons, subset = seurat_clusters %in% c(0, 1, 2, 3, 5)) %>%
  FindVariableFeatures(selection.method = "vst", verbose = FALSE) %>%
  RunPCA(npcs = 30, verbose = FALSE) %>%
  RunUMAP(reduction = "pca", dims = 1:30) %>%
  FindNeighbors(dims = 1:30) %>%
  FindClusters(resolution = 0.5)

cowplot::plot_grid(DimPlot(Lhx6.neurons, reduction = "umap", group.by = "age"),
  DimPlot(Lhx6.neurons, reduction = "umap", group.by = "seurat_clusters"),
  FeaturePlot(Lhx6.neurons, features = c("Ascl1", "Gad2", "Tubb3", "Onecut2")),
  FeaturePlot(Lhx6.neurons, features = c("Zfhx3", "Zfhx4", "Nfia", "Nfib")),
  FeaturePlot(Lhx6.neurons, features = c("Ebf1", "Meis2", "Lhx6", "Sox6")),
  FeaturePlot(Lhx6.neurons, features = c("Pax6", "Satb1", "Sox14", "Hmx3")),
  nrow = 2
)
```

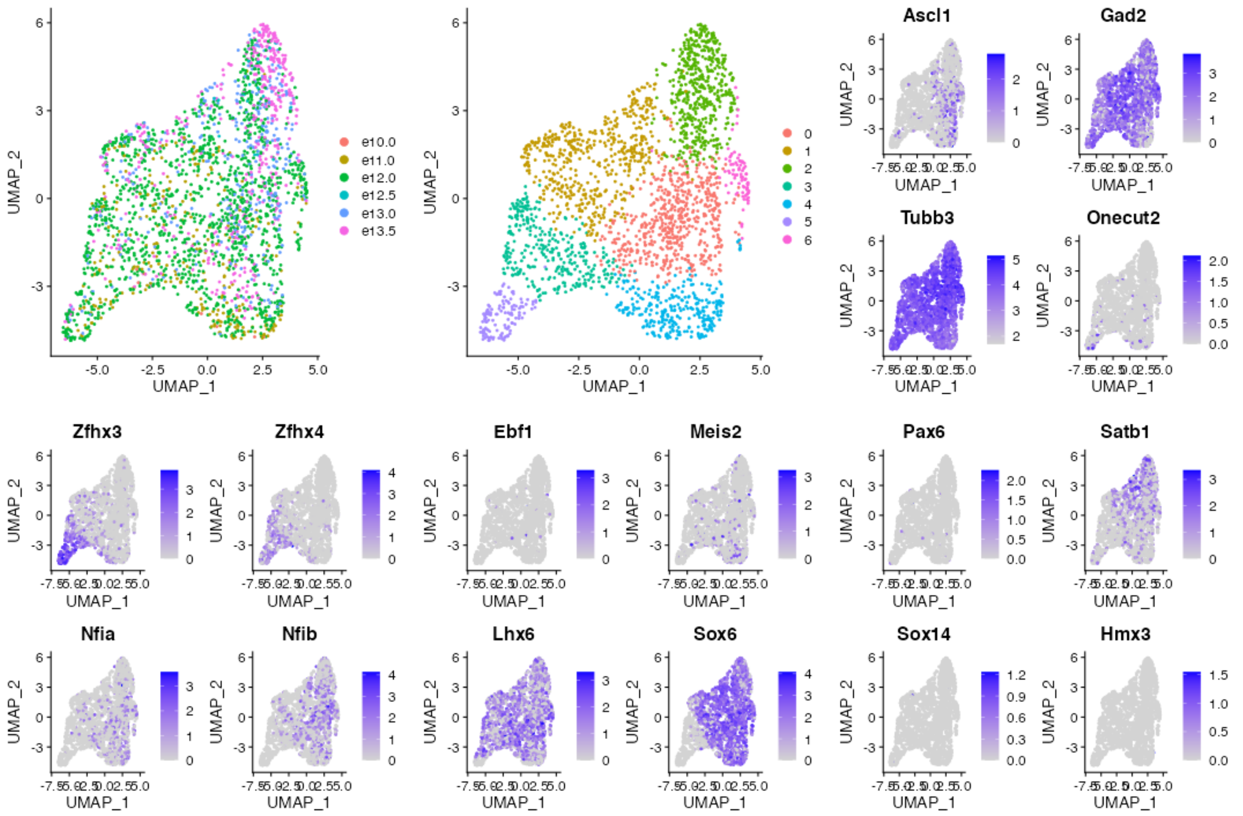

Pseudotemporal ordering using Slingshot

```
sds <- slingshot(Embeddings(Lhx6.neurons, "umap"),
  clusterLabels = Lhx6.neurons$seurat_clusters,
  start.clus = 4, end.clus = c(5, 2), stretch = 0
)

cell_colors_clust <- cell_pal(Lhx6.neurons$seurat_clusters, hue_pal())

plot.pseudotime.curves()
```

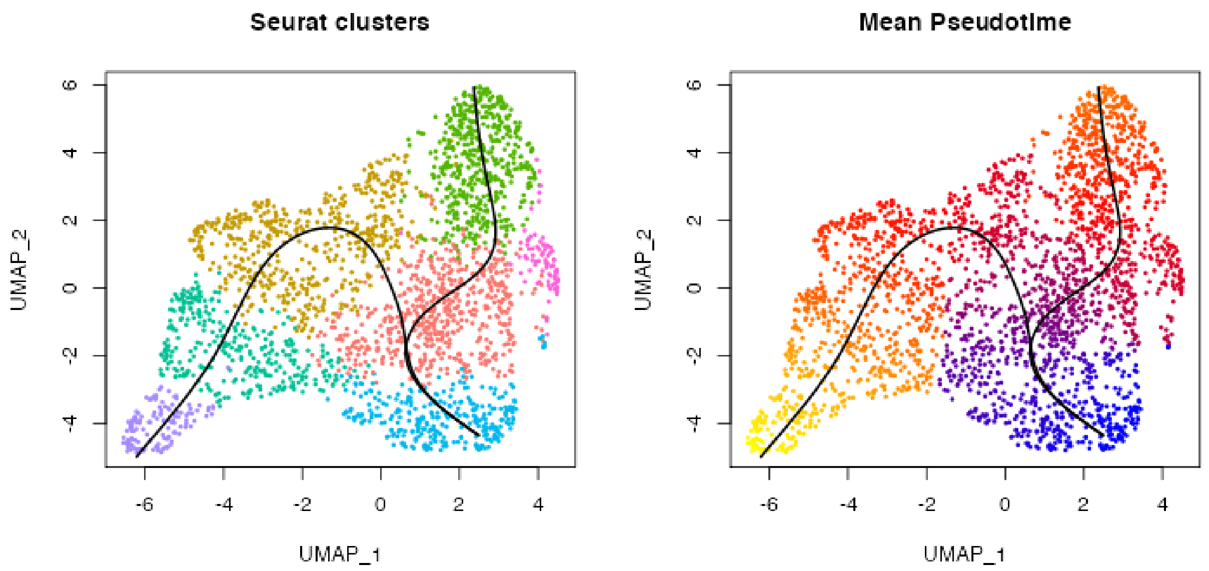

Plot pseudotime profiles of cortical excitatory neurons. We exclude e10.0 and e12.5 because there are only few neurons for these timepoints.

Table 5: Number of cells / timepoint

e10.0	e11.0	e12.0	e12.5	e13.0	e13.5
6	295	1301	18	337	466

```
plot.gene.in.pseudotime.loom(
  gene = c("Ascl1", "Tubb3", "Onecut2", "Zfhx3", "Zfhx4", "Nfia", "Nfib"),
  pt.mtx = slingPseudotime(sds),
  seurat.object = Lhx6.neurons,
  exclude.timepoint = c("e10.0", "e12.5"),
  exclude.curve = NULL,
  plot.title = "Forebrain Inhibitory Neurons (Lhx6+)"
)
```

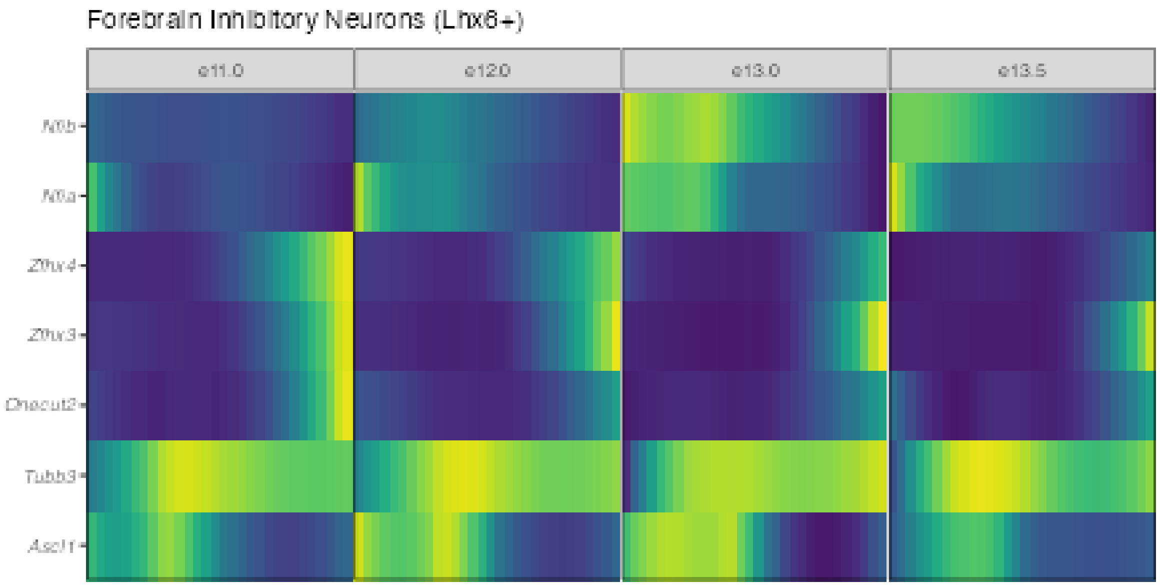


```
plot.expression.dynamics.from.Seurat(
  input = Lhx6.neurons,
  genes = c("Onecut2", "Pou2f2", "Zfhx3", "Zfhx4", "Nfia", "Nfib"),
  time = "age",
  title = "Forebrain Inhibitory Neurons (Lhx6+)",
  from.loom = TRUE
)
```

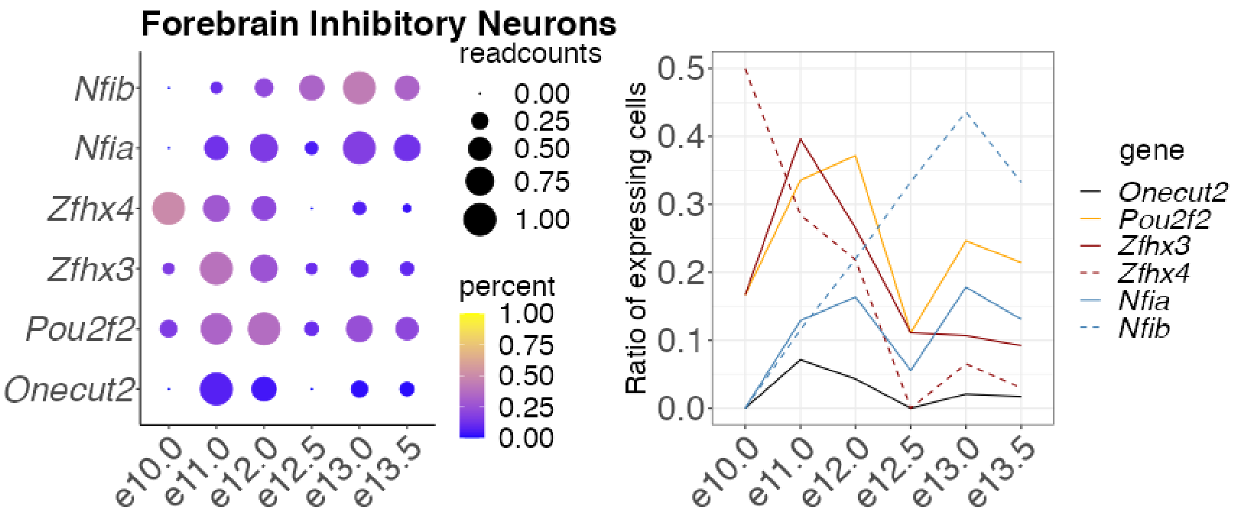

Pseudotemporal gene expression analysis of LGE neurons

Subset data to Meis2/Isl1/Ebf1-positive neurons.

```
LGE.neurons <- cr.neurons %>%
  subset(subset = seurat_clusters %in% c(6, 3, 10)) %>%
  FindVariableFeatures(selection.method = "vst", verbose = FALSE) %>%
  RunPCA(npcs = 30, verbose = FALSE) %>%
```

```
  RunUMAP(reduction = "pca", dims = 1:30) %>%
  FindNeighbors(dims = 1:30) %>%
  FindClusters(resolution = 0.5)

cowplot::plot_grid(DimPlot(LGE.neurons, reduction = "umap", group.by = "age"),
  DimPlot(LGE.neurons, reduction = "umap", group.by = "seurat_clusters"),
  FeaturePlot(LGE.neurons, features = c("Ascl1", "Gad2", "Tubb3", "Onecut2")),
  FeaturePlot(LGE.neurons, features = c("Zfhx3", "Zfhx4", "Nfia", "Nfib")),
  FeaturePlot(LGE.neurons, features = c("Ebf1", "Meis2", "Lhx6", "Isl1")),
  FeaturePlot(LGE.neurons, features = c("Pax6", "Satb1", "Sox14", "Hmx3")),
  nrow = 2
)
```

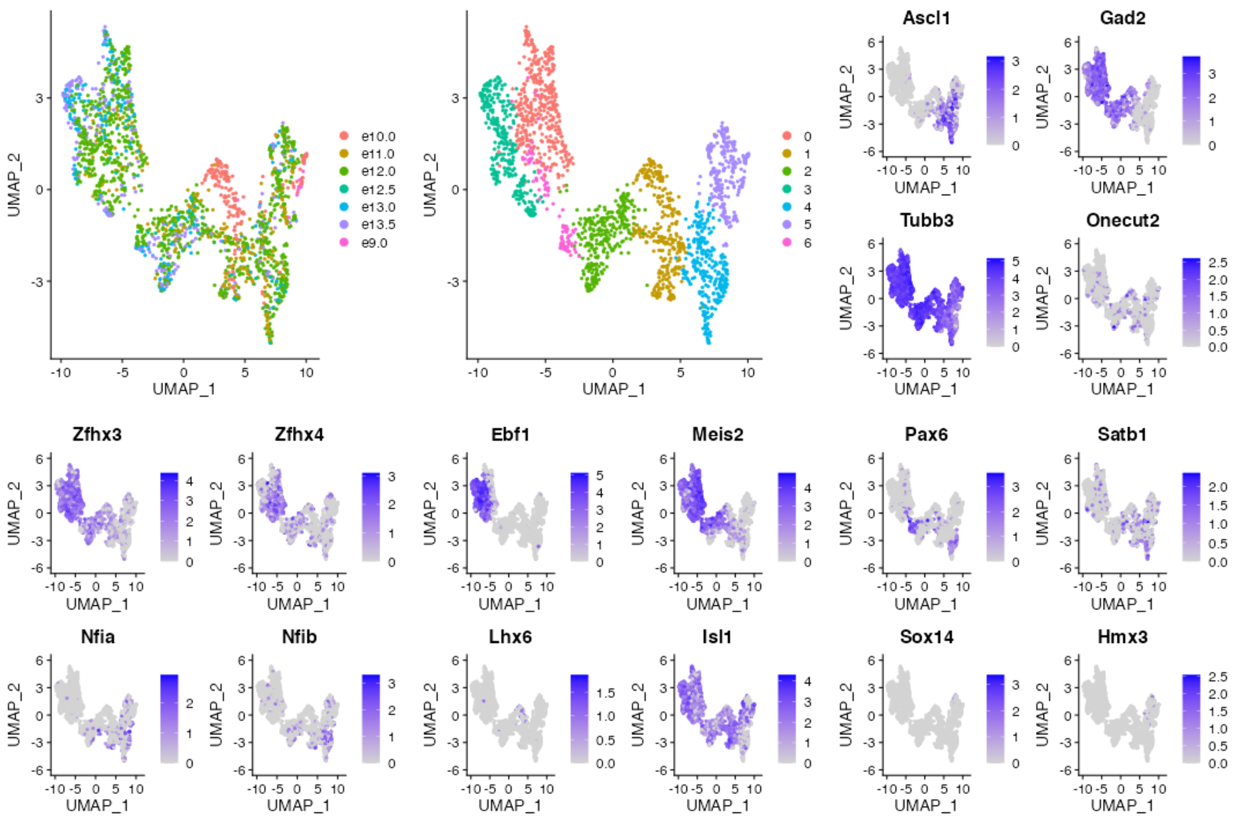

Pseudotemporal ordering using Slingshot

```
sds <- slingshot(Embeddings(LGE.neurons, "umap"),
  clusterLabels = LGE.neurons$seurat_clusters,
  start.clus = 5, end.clus = 0, stretch = 0
)

cell_colors_clust <- cell_pal(LGE.neurons$seurat_clusters, hue_pal())

plot.pseudotime.curves()
```

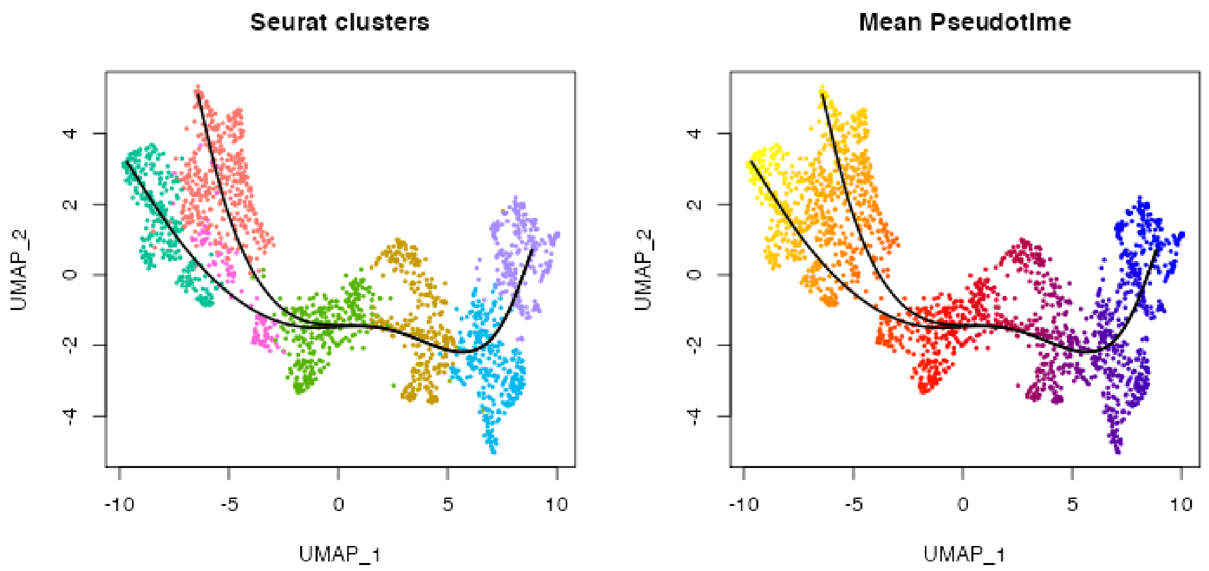

Plot pseudotime profiles of LGE neurons (Ebf1+)

```
plot.gene.in.pseudotime.loom(
  gene = c("Ascl1", "Tubb3", "Onecut2", "Zfhx3", "Zfhx4", "Nfia", "Nfib"),
  pt.mtx = slingPseudotime(sds),
  seurat.object = LGE.neurons,
  exclude.timepoint = c("e9.0"), ## no or few neurons at e9
  exclude.curve = NULL,
  plot.title = "Forebrain Inhibitory Neurons (Meis2+; Isl1+; Ebf1+)"
)
```

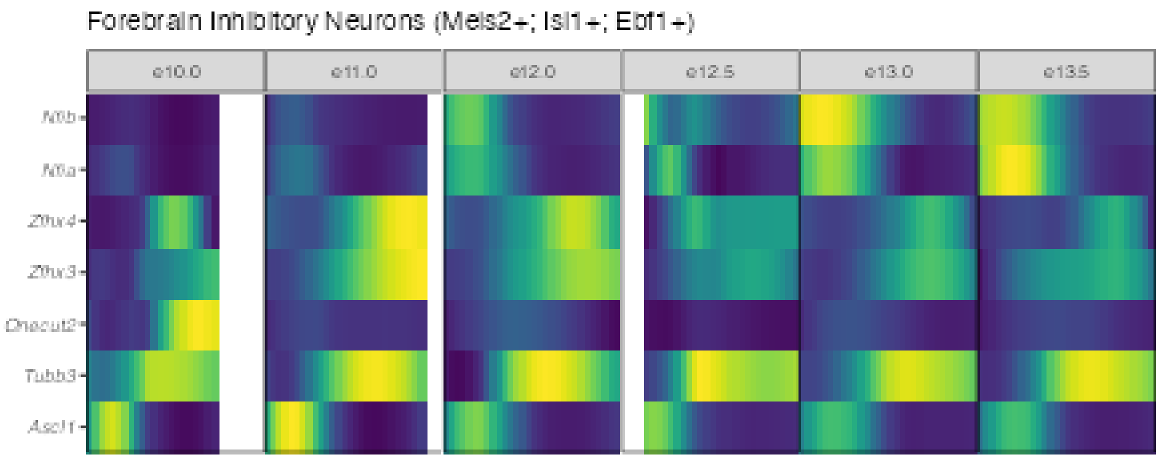


```
plot.expression.dynamics.from.Seurat(
  input = LGE.neurons,
  genes = c("Onecut2", "Pou2f2", "Zfhx3", "Zfhx4", "Nfia", "Nfib"),
  time = "age",
  title = "Forebrain Inhibitory Neurons (Meis2+; Isl1+; Ebf1+)",
  from.loom = TRUE
)
```

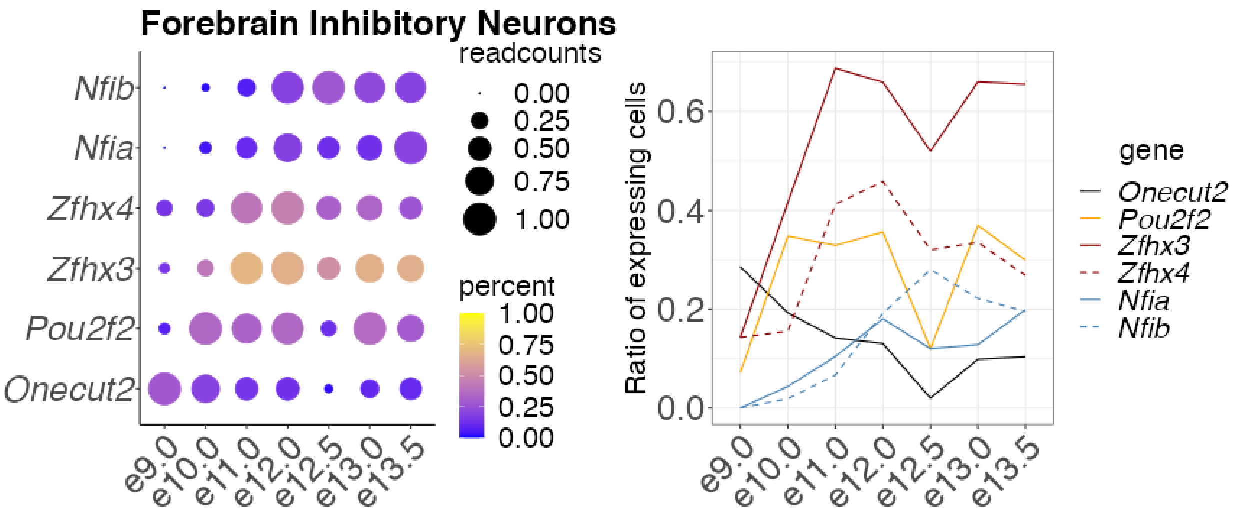


```
### Generate Forebrain-specific Seurat object

tissue <- "Midbrain"
celltype <- "Neuron"
timepoints <- c("e9.0", "e10.0", "e11.0", "e12.0", "e12.5", "e13.0", "e13.5", "e14.0")

tissue.id <- which(grepl(tissue, unique(sc.loom$col.attrs$Tissue[])) == TRUE)
cell.id <- intersect(
  which(sc.meta$tissue %in% unique(sc.loom$col.attrs$Tissue[])[tissue.id] &
    sc.meta$class == celltype),
  which(sc.meta$age %in% timepoints)
)

exp.mat <- sc.loom[["matrix"]][cell.id, ]

colnames(exp.mat) <- sc.loom$row.attrs$Gene[]
rownames(exp.mat) <- sc.meta$cellID[cell.id]

exc.seurat <- CreateSeuratObject(
  counts = t(exp.mat),
  meta.data = sc.meta[cell.id, ] %>%
    as.tibble() %>%
    tibble::column_to_rownames("cellID")
)

exc.seurat[["percent.mt"]] <- PercentageFeatureSet(exc.seurat, pattern = "^mt-")

exc.seurat <- exc.seurat %>%
  subset(subset = nFeature_RNA > 600 & nFeature_RNA < 6000 & percent.mt < 6) %>%
  SCTransform(vars.to.regress = "sampleID") %>%
  NormalizeData(verbose = FALSE, assay = "SCT") %>%
  ScaleData(verbose = FALSE, assay = "SCT") %>%
  FindVariableFeatures(selection.method = "vst", verbose = FALSE) %>%
  RunPCA(npcs = 30, verbose = FALSE) %>%
  RunUMAP(reduction = "pca", dims = 1:30) %>%
  FindNeighbors(dims = 1:30) %>%
```

```
  FindClusters(resolution = 0.5)

cowplot::plot_grid(DimPlot(exc.seurat, reduction = "umap", group.by = "age"),
  DimPlot(exc.seurat, reduction = "umap", group.by = "seurat_clusters"),
  FeaturePlot(exc.seurat, features = c("Neurog2", "Ascl1", "Gad2", "Slc17a6")),
  FeaturePlot(exc.seurat, features = c("Onecut2", "Zfhx3", "Zfhx4", "Nfia")),
  FeaturePlot(exc.seurat, features = c("Nfib", "Neurod2", "Neurod6", "Reln")),
  FeaturePlot(exc.seurat, features = c("Lhx6", "Meis2", "Foxg1", "Isl1")),
  nrow = 2
)
```

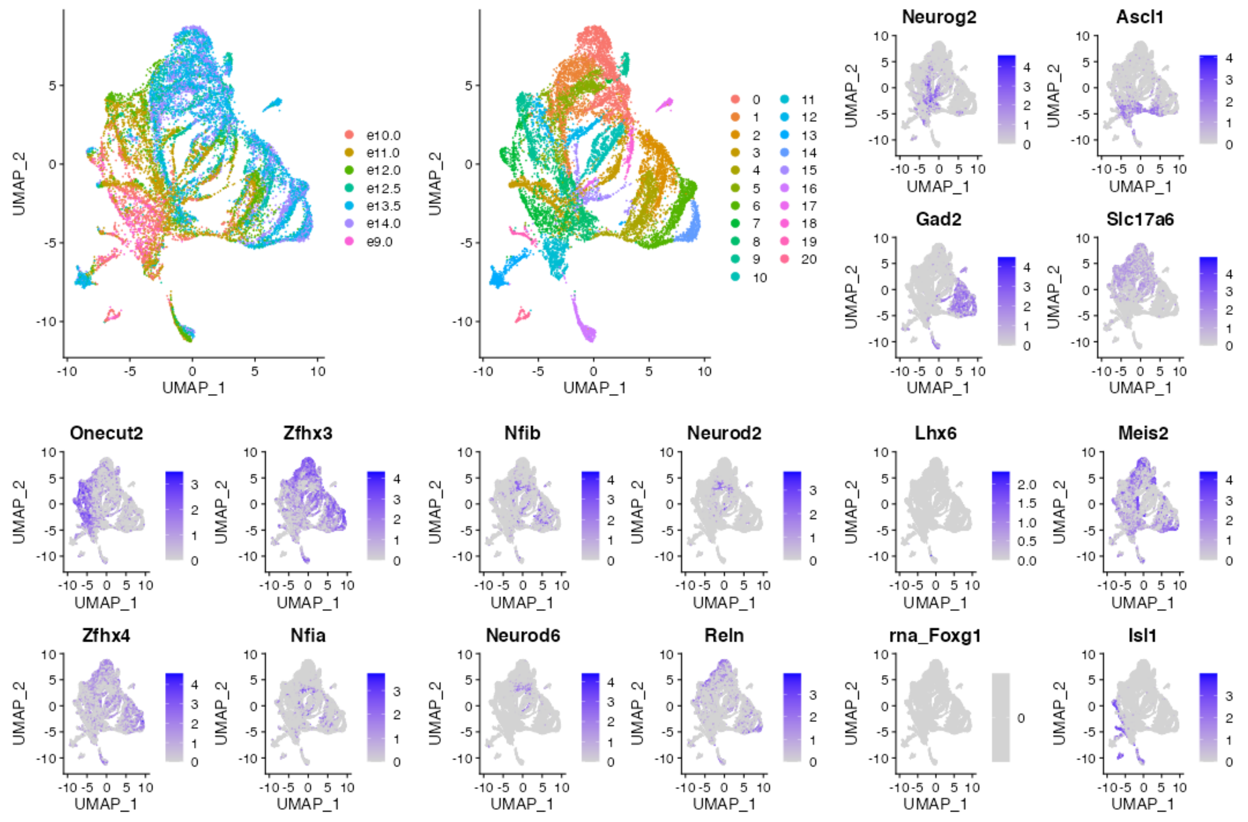


```
mb <- plot.expression.dynamics.from.Seurat(
  input = exc.seurat,
  genes = c("Onecut2", "Pou2f2", "Zfhx3", "Zfhx4", "Nfia", "Nfib", "Neurod2", "Neurod6"),
  time = "age",
  title = "Midbrain",
  from.loom = TRUE,
  colors = c("black", "orange", "darkred", "darkred", "steelblue", "steelblue", "limegreen", "limegreen
  linetype = c("solid", "solid", "solid", "dashed", "solid", "dashed", "solid", "dashed")
)

mb
```

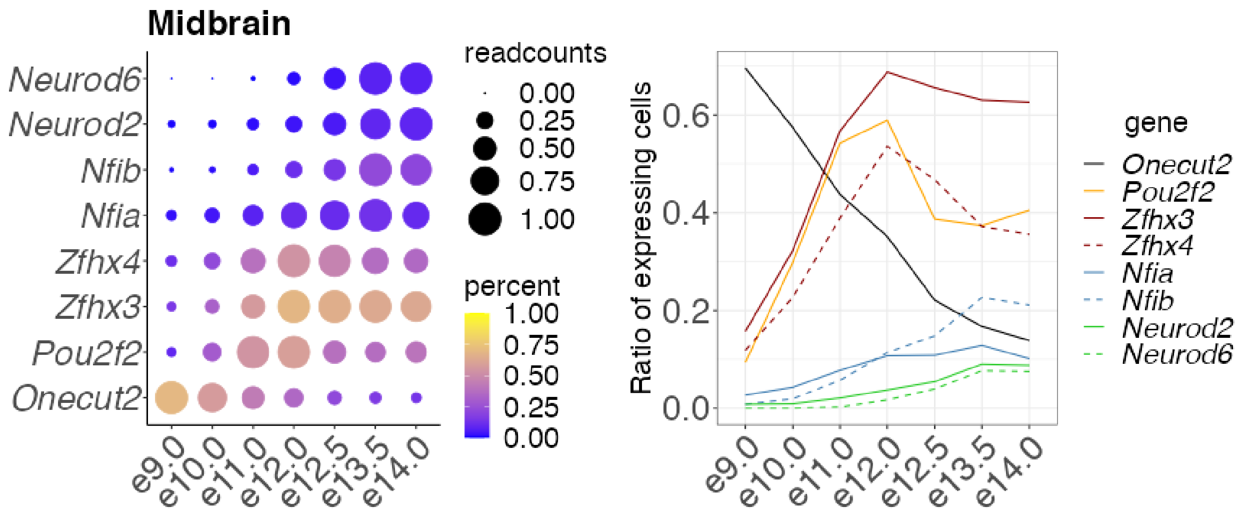


```
cowplot::plot_grid(fb, mb, hb, ncol = 1)
```

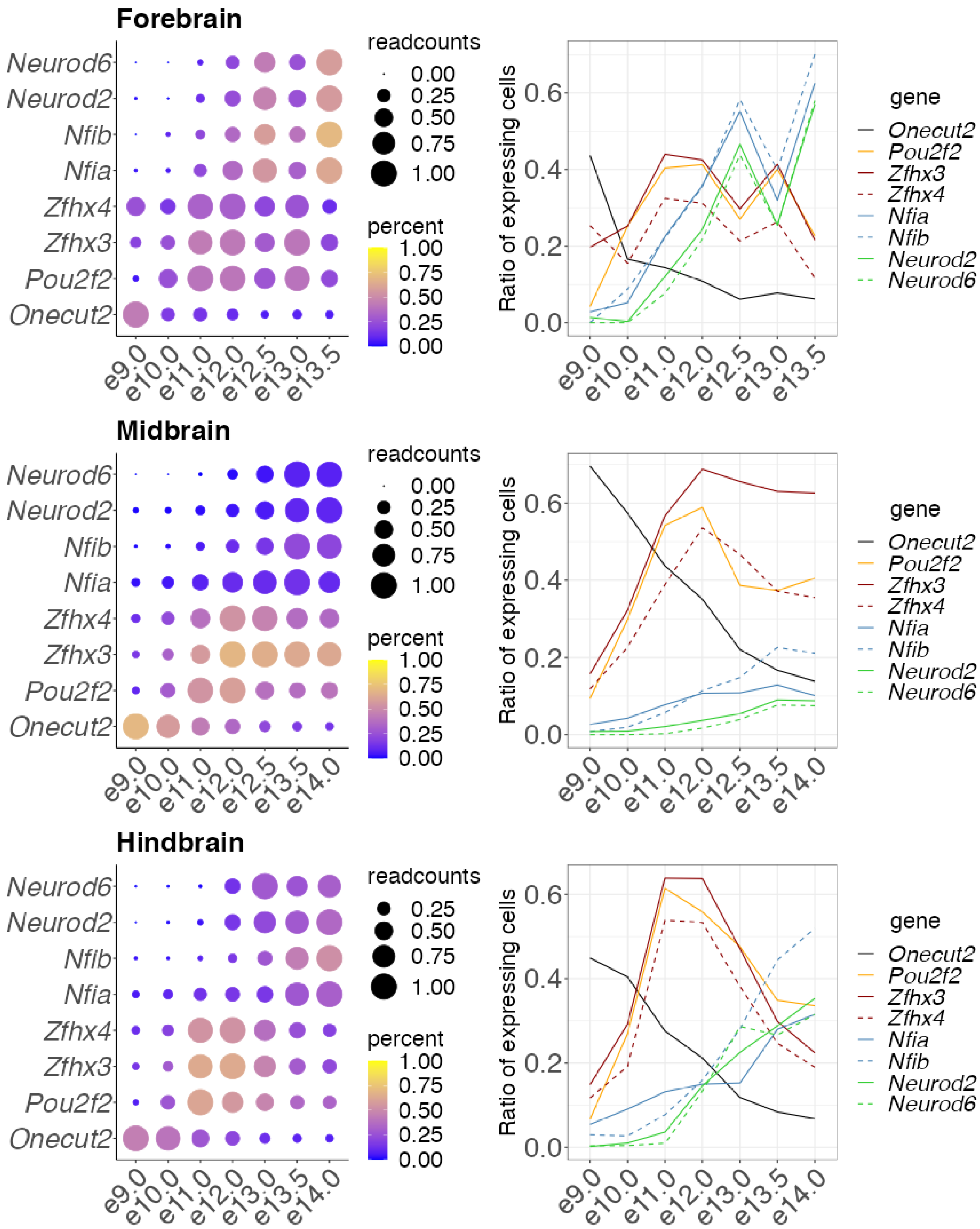
Forebrain
Midbrain
Hindbrain
Neurod6
Neurod2
Nfib
Nfia
Zfhx4
Zfhx3
Pou2f2
Onecut2
e9.0
e10.0
e11.0
e12.0
e12.5
e13.0
e13.5
e14.0
readcounts
0.00
0.25
0.50
0.75
1.00
percent
Ratio of expressing cells
gene
