# Supplementary material for: A shared transcriptional code orchestrates temporal patterning of the central nervous system
Source: PLoS Biol. 2021 Nov 12;19(11):e3001450. doi: 10.1371/journal.pbio.3001450 (PMC8612522; doi:10.1371/journal.pbio.3001450)
Supplement: Attachment — Submitted filename: 115415_1_rebuttal_2082180_qttmt4.docx [file pbio.3001450.s024.docx]

**Response to the referees**

**Reviewer #1 (Evidence, reproducibility and clarity (Required)):** 

Generation of neuronal diversity is regulated by regional and temporal mechanisms. 

In this manuscript, Sagner et al have focused on the latter, and describe a set of transcription factors displaying similar temporal expression dynamics throughout the developing CNS. The authors assign these temporal TFs in three groups based on the order of their expression, from early to late: Onecut-family; Pou2f2 and Zfhx2-4; and Neurod2/6 and Nfia/b. The different groups appear not to co-express, which suggests that these factors are indeed labeling different neuronal subtypes and are not being temporally expressed in the same neurons. 

This temporal behavior of these TFs was recognized and described already in a previous article from the Briscoe lab analyzing developing spinal cord at a single cell resolution (Delile et al., 2019). This work extends those findings to other regions of CNS. The authors use previously published single cell RNAseq data sets to identify a temporal TF code in the developing cortex, retina, midbrain and hindbrain. The authors also focus on TGFbeta as a possible signaling pathway regulating the switch between early-born and later born neuronal subtypes, and they analyze the function of Nfia/b for the generation of late-born neurons. These functional analyses were done in vitro. 

**Major comments:** 

1.) The temporal TFs have been histologically analyzed in midbrain and hindbrain in Figures 2 and 3. The authors claim that TFs belonging to different temporal groups are expressed in "largely non-overlapping populations". However, in Figure 2E and 2F, one can detect several neurons which co-express Pou2f2 and Onecut2 at E11.5, suggesting that Pou2f2 becomes upregulated in some of neurons also expressing Onecut2. At about this time Onecut2 expression starts being downregulated (according to Allen Brain Atlas; it is mostly gone in the midbrain by E13). Is this a staining artefact or is there some extent of co-expression?

We indeed observe rare co-expression between Onecut2 and Pou2f2. Quantification of the proportion of neurons co-expressing these markers suggest that these double-positives comprise less than 2.5 % of neurons at e11.5 (Figure R1C,D). It is currently unclear if these co-expressing neurons are generated when neurons switch from Onecut2 to Pou2f2 expression and may later resolve into one of these 2 states. As the cells co-expressing these markers are rare and the biological significance of this finding is currently unclear, we have not further investigated these.

2.) As the gene expression dynamics in midbrain and hindbrain change rapidly between E9-E13, a more highly resolved analysis dependent on scoring somite stages between the earlier timepoints could be important in order to get a more complete picture. Furthermore, although few Nfib+ cells coexpress Phox2b+ cells in the hindbrain, indicating neuronal identity, the majority does not. To exclude Nfia/b expression in glia at these stages¬, the authors could analyze co-expression with a neuronal marker such as e.g. Rbfox3. 

Following the referees’ advice, we stained hindbrain sections with another well-established nuclear neuronal marker, Lhx5, and observe wide-spread co-expression between Lhx5 and Nfib in numerous nuclei in the mantle layer (Figure R2A). However, Lhx5 is only expressed in neurons derived from specific dorsal-ventral domains in the hindbrain, thus it is expected that not all Nfib-positive nuclei in the mantle layer are labelled by this marker. We further show that the majority of Nfib-positive cells in the mantle layer do not express the progenitor marker Sox2 or the TF Sox9, which is critical for the transition from neuro- to gliogenesis (Figure R2A). Together these data strongly argue that the majority of Nfib-positive cells in the mantle layer are not glial progenitors.

We also note that we provide independent evidence by scRNAseq data analysis that Nfib is widely expressed in inhibitory and excitatory neurons in the hindbrain and other regions of the nervous system (Figure S4 and Supplemental Experimental Procedures). Moreover, we will add data to the manuscript that the expression of Nfia/b is maintained in neurons in the fore- and midbrain until late embryonic stages and that these Nfib-positive cells do not express markers characteristic for glial cells such as Sox9, Aldh1l1, S100b and Slc1a3 (also known as Glast) (Figure R6).

3.) One would assume that early vs late born neurons likely represent different neuronal subtypes not only in spinal cord but also in other brain regions. However these other regions have received considerably less attention than the spinal cord neuronal diversity, especially when it comes to histological validation of single cell sequencing data sets. Birth-dating the early and late born neurons in these other tissues by EdU injections would improve the study. 

We will perform EdU birthdating for Zfhx3 and Nfib positive neurons in the mid- and hindbrain to address this question.

4.) The authors suggest that TGF-beta signaling may regulate the switch from early-born to late-born neurons, and the in vitro data support this idea. However, considering that the temporal switch can be assumed to take place in neural progenitors, there is surprisingly little focus on these. Thus, there are a few key questions to address further: are the different neurons born from the same progenitor domain generated from identical progenitors, or is there a mixture of different progenitors within these domains?

We are surprised that the referee feels there is little focus on temporal patterning in neural progenitors. We include a main text figure that shows conserved expression dynamics of 33 TFs throughout large regions of the developing nervous system (Figure 5A-C). We also validate that the expression dynamics of several of these TFs are preserved in neural progenitors with different axial identities generated in the in-vitro differentiations (Figures 5D,E and S7B) and demonstrate that activating/inhibiting the TGFbeta pathway has opposing effects on the expression of early and late progenitor markers (Figure 6D,F-H). In our opinion these data provide compelling evidence for the existence of a conserved TGFbeta controlled temporal patterning program in neural progenitors.

This comment made us question whether there was too much data in the manuscript and splitting it into two studies might be sensible.

We note that further investigating how temporal patterning in neurons and progenitors are linked requires sophisticated genetic perturbations to manipulate temporal patterning specifically in these cell types. We do not have access to the appropriate transgenes at the moment. This also includes tools necessary to address the question the referee raises about whether these neurons are generated from identical or a mixture of neural progenitors. We agree that this is an extremely interesting question that is very high on our priority list. However, in our opinion generating the genetic tools and carefully analysing the resulting data goes beyond the scope of this manuscript and is probably better suited for an independent follow-up study.

5.) In line with the above, the authors speculate that because Zfhx3 is expressed earlier in dopaminergic neurons, compared to Nfia/b, dopaminergic neurons might represent a temporal neuronal subtype. However, Lmx1a+ floorplate in the midbrain, which generates dopaminergic neurons more caudally and glutamatergic neurons (e.g. the subthalamic nucleus) more anteriorly, can be divided at least into two distinct domains: a Corin1+ medial domain, and a Aldh1a1+/Wnt1+ lateral domain. In the antero-posterior direction, there are likely additional, more graded, subdomains. Thus, without fate-mapping, it is difficult to conclude whether Zfhx3 and Nfia/b expressing neurons are born from the same progenitors by temporal switching or from distinct domains/progenitors. 

We will co-stain dopaminergic neurons for the medial floor plate marker Sox6 (Panman et al.; Cell Reports, 2014, DOI: 10.1016/j.celrep.2014.07.016) and the temporal TFs Nfib and Zfhx3. The outcome of this experiment should allow us to conclude whether each of the populations of dopaminergic neurons generated by the medial and lateral floor plate express Zfhx3 and Nfib.

6.) The authors mention the well-known phenomenon of temporal generation of oculomotor / red nucleus neurons. It would have been interesting to see if the early/late TF code is applicable to these neurons. 

We thank the referee for this great suggestion. Based on the available literature, this switch occurs between e10.5 and e11.5 and should thus coincide with the switch from Onecut2 to Zfhx3-positive neurons. Consistent with this hypothesis, expression of the red nucleus neuron marker Pou4f1 is mutually exclusive with the expression of Onecut2 in the ventral midbrain, while most Pou4f1 neurons express Zfhx3 (Figure R2B). We note however, that we also find a few Zfhx3-positive neurons in this area that do not express Pou4f1. Interestingly, Pou4f1 and Onecut2 are co-expressed in subsets of neurons in the dorsal midbrain, suggesting that these TFs do not always mutually cross-repress each other. We propose to include these data together with the additional characterization of Nfib expression in the hindbrain (response to point 2) as an additional supplemental figure (Figure R2B).

7.) Although challenging manipulating TGF-beta signaling in an in vivo would obviously further strengthen the manuscript.

We agree with the referee but unfortunately have not been able to import the necessary mouse lines during the last year due to the COVID pandemic. However, thanks to the referees’ previous comment, we will provide evidence in the revised version of the manuscript that the induction of the red nucleus neuron marker Pou4f1 temporally coincides with the switch from Onecut2 to Zfhx3 expression in ventral midbrain neurons. Of note, the timing of Pou4f1 induction can be perturbed in-vivo by manipulating TGFbeta signalling (Dias et al., Dev Cell, 2014, DOI: 10.1016/j.neuron.2014.10.033), raising the possibility that perturbations of the TGFbeta pathway in this context will also lead to perturbations in the timing of temporal TF expression.

8.) The analysis of Nfia and Nfib inactivation in vitro is quite limited and the read-out depends on analyzing the expression of a single gene (Neurod2), another marker for the late born neurons. If Nfia/b act in neural progenitors to regulate the switch from earlier-born to late-born neurons, wouldn't the absence of this switch lead to continued production of earlier born neurons? In other words, if the proportion of NeuroD2+ neurons is reduced, what other type(s) of cells are increased? Or is neurogenesis / neuronal differentiation generally affected?

We will add data to main text Figure 7 that shows that the proportion of Zfhx3-positive neurons generated from Nfia/Nfib double mutant ES cells is not increased at day 11 (Figure R3C,D). These data suggest that while Nfia and Nfib promote the generation of Neurod2-positive neurons they do not seem to be required to terminate the competency for the generation of Zfhx3 neurons. Thus, the regulation of the temporal identities seems to be more complicated than in Drosophila, where loss of one temporal TF prolongs the production of earlier-born neurons.

Reviewer #1 (Significance (Required)): 

Temporal regulation remains a key question to address mechanistically in developmental biology and the data in this manuscript provides interesting and novel contributions. The work would have been strengthened if it included also in vivo functional analyses and genetic tools for fate mapping (see specific comments). In addition, the study emphasizes analyses of the spinal cord - which has been analyzed already in the above-mentioned article - whereas other CNS regions have received less attention. For this reason those results are somewhat less convincing. 

**Referee Cross-commenting** 

I agree with Reviewer 3. All reviews raise similar and reasonable concerns. In my original review I did not indicate the time required for revision. Considering the responses from reviewers I would say that a 1-3 month review process (perhaps a bit longer) would be sufficient to substantially improve the manuscript. However, suggested functional in vivo work that would be necessary to provide firm evidence for the suggested regulatory mechanism would require substantially longer time. 

**Reviewer #2 (Evidence, reproducibility and clarity (Required)):** 

Here the authors tackle a very important issue, namely temporal specification of neuronal subtypes in development. They start out based on their previous work with specific candidates that they verify by birthdating experiments in the spinal cord, as well as by staining at different developmental stages in midbrain and hindbrain sections. The authors further corroborate their candidates by analysis of various scRNAseq data. They also use differentiation from pluripotent stem cells into progenitors with different regional identities confirming early, middle and late expression of their candidate TFs. They then proceed beyond these TFs and identify a set of further TFs (33) showing temporal progression, even though many of these are related to glioagenesis and hence not novel. In the last part of their manuscript the authors progress to search for factors regulating this temporal progression and convincingly demonstrate a role of TGFb in vitro. Elevating TGFb levels promotes earlier appearance of the late TFs at mRNA lavel, and inhibition reduces and delays their expression. Finally, the authors generate Nfia/b knock-out ES cells and demonstrate reduced NeuroD2+ cells by FACS. 

To improve the quality of the manuscript I have the following suggestions: 

1) the read-outs of the temporal TFs differ throughout the manuscript, immunostaining, FACS, RT-PCR. To ensure comparable data I suggest to use at least one common read-out in all. As protein matters most, I suggest to complement Figure 6 by protein analysis, quantifying immunopositive nuclei for Onecut2, Zfhx3, Nfia or Nfib and Neurod2. As the authors show beautiful immunostaining in Figures 1-3, this should definitely also work in vitro.

The fact that we obtain consistent results using different assays is a strength, not a weakness, in our opinion. Moreover, flow cytometry is a well-established technique and widely used for the quantification of cells. The technique has several additional advantages over immunofluorescence. These are (1) that more markers can be analysed in parallel and in combination with life-dead cell dyes, (2) that it provides an unbiased quantification of cells within a differentiation, which is difficult by immunofluorescence due to the inherent spatial heterogeneities that emerge during the in-vitro differentiation as a consequence of the cells growing in colonies, and (3) that cells from the same dish can be split into different batches and therefore subsets of the same cell population can be stained for different markers. In response to the referees’ comment, we have performed a quantification of immune-positive nuclei by immunofluorescence. This analysis revealed similar expression dynamics for the temporal TFs as the flow cytometry data (Figure R4).

2) For Figure 7 please add at least example pictures of immunostaining and show the cells stained with the secondary antibody control to allow determining the correct gate selection.

We will add example pictures showing Neurod2-staining in Nfia/b double mutant and control differentiations. Gates for Neurod2 were determined based on Sox2+ neural progenitors in the same samples as for the rest of the manuscript (see Figure S6B). Neural progenitors lack Neurod2 expression and therefore serve as suitable internal controls. In response to the referees’ comment we have added the intensity distributions obtained from the neural progenitors and the intensities for which neurons were counted as positive to Figure 7 (blue lines and grey shading in Figure R3C).

3) The EdU birthdating experiments show early generated neurons several days after their birthdate versus late generated neurons only 1-2 days after their birth. The authors also mention that the factors persist for some while but show no evidence for this. I wiuld therefore recommend to add a comparable time window for the late born neurons, i.e. E12.5 + 4 days, E16.5 (as they show E9.5 EdU until E13.5). This allows to compare neurons at the same differentiation state. 

Experiments similar to those proposed by the referee have been recently conducted and published by the lab of Samuel Pfaff (Osseward et al.; Science; 2021; DOI: 10.1126/science.abe0690). At perinatal stages the temporal TFs also label different populations of neurons with different birth dates in the spinal cord. We will mention these results in our manuscript.

4) There are many beautiful temporal data sets of scRNA-seq from the Arlotta and the Jabaudon labs for the cerebral cortex development, but they are neither cited nor analyzed. As temporal progression of cortex neurons is as one of the best established models for temporal progression of neuronal subtype generation, it would be great if the authors could include some data on this region. There is also beautiful ES differentiation protocols of cortex neurons progressing in time from the vanderHaeghen lab that would be good to consider, at least for existing (sequencing) data. 

We propose to add an analysis of scRNAseq data from cortical neurons (Figure R5) as a main text or supplemental figure to the manuscript. This analysis shows that most cortical neurons express the same markers that are characteristic for a late temporal identity in other parts of the nervous system such as Nfia/b, Tcf4 and Neurod2/6 (Figure R5F). This data is consistent with the relatively late onset of neurogenesis in the cortex compared to other regions of the nervous system, a large body of previous work that shows expression of Nfia/b/x and Neurod2/6 in most cortical neurons and the wide-spread usage of Neurod6-Cre (also known as Nex-Cre) to label cortical excitatory neurons. Of note, our analysis also identified a population of Zfhx3-positive forebrain excitatory neurons (cluster 7 in Figure R5D) that, consistent with the idea of the temporal code, seem to be generated earlier than the Nfia/b/x Neurod2/6 cortical neurons (Figure R5C-F). Further analysis of the expression profile of this Zfhx3-positive population revealed the TFs Pbx3, Meis1/2, Tshz2 and Barhl2 as markers for these neurons (Figure R5E). These observations are consistent with a recent study by Moreau et al. (bioRxiv, 2021) who identified the same set of neurons from cortical scRNAseq data from e12.5 embryos and show that these neurons localize to the pallial-subpallial boundary.

5) The TGFb treatments are done exclusively in vitro, but knockout mice should be available to probe this hypothesis also in vivo. If not experimentally, the authors should at least discuss the evidence from the literature. 

We are surprised that the referee feels we do not discuss the evidence from the literature about the role of TGFbeta signalling in temporal patterning because we dedicate a large section of the Discussion to this topic. We also summarize the in our opinion most important pieces of previous evidence in the 2^nd^ but last section of the introduction. Furthermore, the referee did not indicate any specific literature or points she/he would like us to discuss in more detail. This makes it difficult to decide which other evidence the referee would like us to include.

6) The same applies for Nfia/b factors - they have been implicated actually also in late generated neurons, namely adult neurogenesis (Ninkovic et al. 2013) and in various defects in corticogenesis, but they are already expressed in early generated neurons in the cortex (see Figure 1 of Bunt et al., 2017). These findings should at least be discussed.

We will add data to the manuscript that argues that the majority of cortical neurons generally have a late-born temporal identity (Figure R5; see response to point 4). These observations are consistent with the late onset of neuron generation in the cortex relative to other regions of the nervous system and the wide-spread expression of Nfia/b in cortical neurons pointed out by the referee. We will also discuss the previous work about the role of Nfia/b in corticogenesis and adult neurogenesis.

Reviewer #2 (Significance (Required)): 

This manuscript tackles a very important issue, namely how temporal progression of generating neuronal diversity is regulated. This is a very broad conceptual issue of outstanding importance and relevance. Especially the concept that the same factors may act throught the nervous system, i.e. across differentially patterned regions, is fascinating and crucial for development and repair when specific neuronal subtypes are attempted to be regenerated. Especially across the entire neuraxis very little is known and this work hence proposes a pioneering concept. As the authors link their work even to the Dorsophila model, it would be fascinating to include discussion of other vertebrate models, like zebrafish or chicken. 

A recent preprint shows that the same temporal patterning we observe in the mouse is also present in the human spinal cord (Rayon et al., bioRxiv, 2021). We will mention these new findings in the discussion.

**Reviewer #3 (Evidence, reproducibility and clarity (Required)):**

The manuscript by Sagner et al identified a universal set of temporal transcription factors marking neurons born at different developmental times. Identification of such temporal program provides a powerful system that expands neuronal diversity in the CNS. The authors demonstrate that temporal progression is in part controlled by TGFbeta signaling and that late temporal factors Nfia/b are required for specification of late born neuronal identities.

The data are in agreement with prior studies that identified Nfi factors as determinants of late born neuronal cells and TGFbeta factors as key drivers of late born neuronal specification. Overall, generalization of these principles to the rest of the CNS is an important contribution that has implications not only to our understanding of how neuronal complexity is generated, but also provides guidance for controlled differentiation of specific neuronal cell types from pluripotent stem cells in vitro. The weakest point of the study is the analysis of published scRNA-seq data that is in places confusing and does not necessarily support the idea of a universal temporal program. Finally, the model would be much stronger if the identified temporally regulated transcription factors expressed in progenitors were functionally tested for their ability to control sequentially generated neuronal subtypes.

1. Conclusions based on EdU administration on day 12.5 with analysis on day 13.5 is complicated by the fact that EdU labeled cells are mostly progenitors that express Nfib and nascent neurons that might not yet downregulated this transcription factor. Do neurons labeled with EdU on e12.5 retain expression of Nfib when analyzed on e15.5 or later?

Recent work in the spinal cord by the Pfaff lab suggests that this is indeed the case and that these TFs label largely non-overlapping populations of neurons born at different developmental stages also in the perinatal spinal cord (Osseward et al.; Science; 2021; DOI: 10.1126/science.abe0690). We will mention this data in the manuscript.

2. How long do postmitotic neurons express the temporal transcription factors? Are these factors expressed in postnatal stages and do neurons retain mutually exclusive expression of Nfib and Zfhx3?

Recent work by the Pfaff lab suggests that the non-overlapping expression of late (Nfib/Neurod2) and intermediate (Zfhx3/Zfhx4) TFs is maintained in the perinatal spinal cord, and for Neurod2/Zfhx3 even up to P70 (Osseward et al.; Science; 2021; DOI: 10.1126/science.abe0690). These results suggest that neurons retain the mutually exclusive expression of the temporal TFs at least in some cases. To further explore if this is also the case in other regions of the nervous system, we analyzed midbrain (Figure R6A-C) and forebrain (Figure R6D-F) scRNAseq data from late embryonic stages (e16-e18) from La Manno et al. 2020. These data suggest that intermediate (Zfhx3, Zfhx4, Pou2f2) and late (Nfia, Nfib, Nfix, Neurod2, Neurod6, Tcf4) TFs stay highly anticorrelated in these regions of the nervous system too, suggesting that the mutually exclusive expression in these regions is also broadly retained at least until late embryonic stages. We propose to include this new data as an additional supplemental figure.

3. How do the authors reconcile expression of Nfib and Nfia in spinal motor neurons (Deneen et al., 2006; Matuzelski et al, 2020), considering that motor neurons are born early.

Glasgow et al. (Nature Neurosci; 2014; DOI: 10.1038/nn.4638) have demonstrated that the e96 distal Nfia enhancer is specifically activated in motor neurons and controlled by Lhx3/Isl1, main determinants of somatic motor neuron fate. Thus, the spatial and temporal gene regulatory networks seem to cross-regulate each other in this case, explaining why Nfia expression is wide-spread in embryonic motor neurons despite their early generation. Expression of Nfib may be downstream of Nfia or controlled by a similar yet uncharacterized distal enhancer.

4. The expression data from the retina, as presented in Figure S3D almost supports the hypothesis that Onecut, Pou2f2, Zfhx3, and Nfib are sequentially turned on in maturing neurons. This is mainly because the expression of early TFs (Onecut, Pou2f2) does not persist in a population of cells at the later time points (as would be expected based on the proposed hypothesis as shown in Figure S5C). For example RGCs on e14 express Onecut2 factor, but RGC in e18 samples are Onecut2 negative, suggesting that the cells downregulated expression of this transcription factor. This could simply be because Onecut and Pou2f2 are only transiently expressed in the early cell types.

We agree with the referee that the expression of some of the temporal TFs may only be transient. This is in fact well known for Onecut TFs in the spinal cord, which, as we mention in the manuscript, are also only transiently expressed there but control the localization and identity of neuronal subtypes in several cases. Moreover, many TFs that define spatial identities are also only transiently expressed but have important functions that define the subsequent development of the neuronal lineage (Lai et al.; Development; 2016 DOI: 10.1242/dev.139592). We will discuss this point more rigorously also in the context of the new data showing maintained expression of some of the other temporal TFs in other regions of the nervous system and the well-known maintained expression of the temporal TFs in Drosophila.

5. Retinal cells also seem to exhibit a highly cell-type dependent temporal code, as shown in S3D - late born ACs do not express Nfib factor but express Zfhx3. In contrast, late born rods and cones do not express Zfhx3 but express Nfib factor. This data is not consistent with the proposed universal temporal transcriptional program.

We partially agree with the referee. First, we would like to emphasize that while we say that this temporal program is widespread during embryonic development of the nervous system, we do not want to imply that it is absolutely universal and applies to every last neuronal cell type. We will rephrase the manuscript accordingly and also point out the example of the missing Zfhx3 expression in rods and cones the referee mentions. We note however, that other parts of the temporal code such as the expression of Onecut TFs in early cones and Nfib expression in late cones and rods seem to apply to photoreceptors.

We respectfully disagree that late-born amacrine cells do not express Nfi TFs. Subsets of glycinergic and non-glycinergic non-gabaergic (nGnG) amacrine cells, which are generated after gabaergic amacrine cells (Voinescu et al.; J Comp Neurol; 2009; DOI: 10.1002/cne.22200), do actually express Tcf4, Nfi TFs and Neurod2/6 (Yan et al., bioRxiv, 2020, DOI: 10.1101/2020.03.10.985770; Cherry et al., J Neurosci, 2011, DOI: 10.1523/JNEUROSCI.2555-10.2011). These cells are rare in the scRNAseq dataset we analyzed, probably because many other cell types are generated in the retina at the stage these cells emerge. We will cite this data in the revised version of the manuscript.

6. The global analysis of scRNA data in brain indicates that Onecut and Zfhx3 expression is extinguished in later timepoints (Fig 2A), but it is not clear whether this is due to a dilution of positive cells by later born neurons or due to a downregulation of expression. The authors should use scRNA data to define what fractions of inhibitory (and excitatory) neurons express temporal transcription factors at each developmental stage. Such analysis would provide a more direct support to the hypothesis that the temporal transcription factors define distinct subtypes of inhibitory (and excitatory) neurons.

For the forebrain, midbrain and hindbrain, the fraction of neurons expressing the respective temporal TFs at each developmental stage is indicated by the colour code in Figure 2A. In response to the referees’ comment we will add plots like the one shown in Figure R7 to the supplemental experimental procedures for forebrain, midbrain and hindbrain neurons and each of the neuronal subsets for which we perform a pseudotemporal gene expression analysis in Figure S5.

The role of the temporal TFs in specifying distinct subtypes of neurons has been previously demonstrated in several cases, which we cite in the introduction and discussion. This includes the role for Onecut TFs in specifying Renshaw cells and LMCm neurons in the spinal cord or early-born cell types in the retina, the role of Nfi TFs in specifying late neuronal identities in the retina or the role of Neurod2/Neurod6 in controlling neuropeptide expression in inhibitory neurons in the dorsal spinal cord. Moreover, recent work by Samuel Pfaff’s group showed that the temporal TFs Zfhx3 and Nfib/Neurod2 define projection and local neurons in the spinal cord (Osseward et al.; Science; 2021; DOI: 10.1126/science.abe0690). We will also add new data to the manuscript that the onset of expression of the red nucleus marker Pou4f1 in the midbrain correlates with the switch from Onecut2 to Zfhx3 expression in neurons (Figure R2B; see response to point 3 raised by referee 1). Together, these data strongly support the hypothesis that the temporal TFs define molecularly and functionally distinct subtypes of neurons in large regions of the nervous system in our opinion.

7. Similarly, it is not addressed whether the Phox2b neurons express first Zfhx3 and then transition to Nfib expression or whether these are two distinct populations of cells. Sequential birthdating studies with two thymidine analogues would resolve this issue.

We will provide EdU birthdating data for Nfib and Zfhx3 neurons in the hindbrain in the revised version of the manuscript. We further note that our analysis of the pseudotemporal gene expression dynamics along numerous neuronal differentiation trajectories (Figure S5) argues against wide-spread switching from a Zfhx3 to an Nfib-positive neuronal subtype. This is because such switching should result in Nfib expression typically occupying later pseudotime bins than Zfhx3 along the neuronal differentiation trajectories. However, after its onset Nfib expression is typically elevated in earlier pseudotime bins than Zfhx3 expression (Figure S5). These observations are consistent with the sequential generation of Zfhx3 and Nfib-positive neurons and argue against wide-spread switching from Zfhx3 to Nfib-expression in our opinion.

8. Considering the well-documented inside-out development of cortical plate, the authors should include immunohistochemical analysis of the temporal factors expression in neocortex and their co-localization with markers of pyramidal neurons populating individual cortical layers.

We propose to add an additional analysis of cortical scRNAseq data to the manuscript that addresses this point (Figure R5; see response to point 3 raised by referee 2).

9. Figure S5 is very difficult to interpret. It is unclear how the color scale represents pseudotime in D-F, and also unclear what the x-axis of these plots are. In B, yellow is a late timepoint while blue is early, but this does not seems to be consistent in D-F?

We thank the referee for pointing this out. The colour scale in B was supposed to represent pseudotime, while in the rest of the figure the same colour code was supposed to represent levels of gene expression. The x-axis in D-F represents pseudotime individually plotted for each timepoint. We have changed the colour code for panel B, clearly labelled the x-axes in the plots and added legends what the colour codes in the individual panels represent (Figure R8). We hope these changes make the figure easier to interpret.

10. The authors test the effect of Nfia/b knockouts on Neurod expression in Figure 7. It would be interesting to determine how loss of Nfia/b changes the proportions of neuron types produced in the differentiations. Do the progenitors remain "stuck" at the previous transcription factor Zfhx3 and produce more of those cells, or the proportions of cells do not change with the exception of a loss of Nfia/b cells?

We will add data to the manuscript that addresses this point (Figure R3C,D; see response to point 8 raised by referee 1). Interestingly, we do not observe an increase in the proportion of Zfhx3-positive neurons in the Nfia/Nfib double mutants, suggesting that while Nfia and Nfib seem to promote the expression of Neurod2 they do not seem to be required to terminate the production of Zfhx3-positive neurons.

11. Postembryonic neuroblasts in drosophila show great diversity in the transcription factors that constitute the temporal selector cascades. It is striking that the authors find the same transcription factors involved in the temporal cascades throughout the mouse nervous system. It might be interesting to bring this point up in the discussion. 

We agree with the referee and will discuss this point more extensively in the discussion.

Reviewer #3 (Significance (Required)): 

Highly significant findings that extend previously established role of Nfi factors and TGFbeta signals in the control on temporal progression in neurogenesis. 

**Referee Cross-commenting** 

All three reviewers raised very similar concerns that should be carefully addressed in the revision. I do not see any points that would seem unreasonable.

**Fig. R1: Quantification of Onecut2 / Pou2f2 double-positive neurons**

(A) Midbrain section at e11.5 stained for Onecut2 (red), Pou2f2 (green) and the progenitor marker Sox2 (blue)
(B) Nuclei in the section were segmented based on DAPI staining and cells classified as progenitors (blue) and neurons (salmon) based on Sox2 levels
(C) Levels of Onecut2 and Pou2f2 in individual neuronal nuclei plotted against each other. Cells were classified as Onecut2- and Pou2f2-positives based on manual chosen intensity thresholds. Note that only a small number of cells co-expresses Onecut2 and Pou2f2 (dark yellow).
(D) Fraction of double-positive neurons from 4 embryos each at e10.5 and e11.5. Note that the fraction of neurons classified as double-positives typically does not exceed 2.5 % of all neurons.
(E) Example images from the region boxed in A showing the behaviour of the classification (colour code as in C) overlaid over DAPI (all nuclei), Onecut2 and Pou2f2 staining.

**Fig. R2: Further characterization of temporal TF expression in the hind- and midbrain**

(A) Hindbrain sections at e13.5 stained for the progenitor marker Sox2, the gliogenic progenitor marker Sox9 and the neuronal marker Lhx5 (from top to bottom). Note that Nfib-positive cells in the mantle layer do not express Sox2 and Sox9, but many cells coexpress Lhx5.
(B) Midbrain sections at e11.5 stained for the red nucleus marker Pou4f1 and the temporal TFs Onecut2 and Zfhx3.

We propose to add this data as an additional supplemental figure to the current manuscript.

**Fig. R3: Updated version of Figure 7**

(A) Analysis of Nfia and Nfib ChIP-Seq data from the mouse cerebellum (Fraser et al., 2020) confirms binding of Nfia and Nfib to genomic regions in the vicinity of the Neurod2 gene.
(B) Analysis of ChIP-seq data from the ENCODE project (Gorkin et al., 2020) reveals accumulation of H3K27ac at the same sites between e11.5 and e13.5 in the different regions of the nervous system.
(C) Neurod2 (top) and Zfhx3 (bottom) intensity histograms from three control (left) and *Nfia; Nfib* double mutant (right) differentiations. Blue curves indicate intensity histograms from Sox2-positive progenitors that do not express Neurod2 and Zfhx3. Red curves show histograms from neurons, identified based on Tubb3 staining. Grey shading indicates the applied thresholds above which neurons were counted as Neurod2 positive.
(D) Quantification of the percentage of Neurod2- (top) and Zfhx3-positive (bottom) neurons at D11 in control and *Nfia; Nfib* double mutants differentiated in ventral conditions (n=6 for control and n=3 for *Nfia; Nfib* double mutants). The quantification reveals a strong reduction of Neurod2 neurons but no change in the fraction of Zfhx3 neurons in the absence of Nfia and Nfib. Significance was assessed by unpaired t-test with Welch’s correction.

We propose to replace Figure 7 with this updated version.

**Fig. R4: Quantification of temporal TF expression in the in-vitro differentiations by immunofluorescence**

(A) Dorsal differentiations stained for the temporal TFs Onecut1 (early; red), Zfhx3 (intermediate; blue) and Neurod2 (late; green) and the pan-neuronal marker Tubb3 (white).
(B) Quantification of Onecut1, Zfhx3 and Neurod2 neurons reveals similar expression dynamics of the temporal TFs as our characterization of flow cytometry.

**Fig. R5: Characterization of temporal TF expression in cortical neurons**

(A) UMAP plots of all e10 – e13.5 forebrain neurons in the dataset from La Manno et al. 2020. Cortical excitatory neurons are coloured in red.
(B) UMAP plots indicating the expression of marker genes characteristic for cortical excitatory neurons.
(C) UMAP plot of cortical excitatory neurons (red cells in A) colour-coded for the developmental stage from which these cells were obtained.
(D) Identification of different clusters of cortical excitatory neurons. Cluster 7 corresponds to the Zfhx3-positive population of neurons (see also F).
(E) Differential gene expression analysis comparing cluster 7 cells to the rest of the identified cortical excitatory neurons. The top 6 TFs upregulated in this cluster are indicated by the red box, the top 6 down-regulated TFs by the blue box. Note that this analysis recovers the subdivision between neurons expressing Zfhx3 and neurons characterized by the expression Nfia/Nfib/Nfix and Neurod2/Neurod6 we previously characterized throughout the neuraxis.
(F) UMAP plots indicating the expression of intermediate (Pou2f2, Zfhx3) and late (Tcf4, Nfia, Nfib, Neurod2, Neurod6) temporal TFs in forebrain excitatory neurons. Note that expression of Zfhx3 correlates with expression of Pbx3 in cortical neurons at early developmental stages (compare with colour code in C). Further note that the majority of cortical excitatory neurons expresses markers characteristic of a late temporal identity (Tcf4, Nfia, Nfib, Neurod2, Neurod6).
(G) Correlation rank plots for Zfhx3 (top) and Nfib (bottom). Expression of Zfhx3 and Nfib are strongly anti-correlated in cortical excitatory neurons similar to the rest of the nervous system (see Figure S4M).

We propose to add this data as an additional main text or supplemental figure to the manuscript.

**Fig. R6: Zfhx3 and Nfib expression are maintained until late embryonic stages in the mid- and forebrain**

(A,D) UMAP plots from late midbrain (A) and forebrain (D) neurons (e16 – e18) showing expression of the indicated markers. Note that expression of intermediate and late temporal markers (especially Zfhx3/4 and Nfia/b) stay highly anticorrelated in neurons. Nfia and Nfib-positive cells are neurons and not of glial origin because they express the neuronal markers Tubb3 and Elavl3 but not the glial markers Sox9, Aldh1l1, S100b and Slc1a3.

(B,E)	Heatmaps indicating Spearman correlation between intermediate and late temporal TFs in the late embryonic midbrain (B) and forebrain (E).

(C,F)	Correlation rank plots for Zfhx3 and Nfib indicate that these markers stay highly anti-correlated in the late embryonic midbrain (C) and forebrain (F).

We propose to add this data as an additional supplemental figure to the manuscript.

**Fig. R7: Indication of the fraction of temporal TF expressing neurons from scRNAseq.**

We will add diagrams like the one above that indicate the fraction of neurons expressing the respective temporal TFs in the scRNAseq data to the supplemental experimental procedures.

**Fig. R8: Updated version of Figure S5**

(A)	Sequential generation of neurons expressing temporal TFs should result in the capture of neurons at different stages of their differentiation trajectory in scRNAseq timecourse data.

(B) Pseudotime reconstruction of gene expression dynamics along a neuronal differentiation trajectory. Blue corresponds to early cells, red to intermediate and yellow to late cells along the differentiation trajectory. Arrows indicate predicted pseudotime trajectories.

(C)	Temporal TFs should be sequentially expressed in pseudotime.

(D)	Pseudotime reconstruction of gene expression for different neuronal lineages along the dorsal-ventral axis of the spinal cord reveals sequential expression of temporal TFs. The colour code indicates the normalized level of gene expression along pseudotime. Pseudotime was independently plotted for each timepoint along the x-axis from left to right.

(E,F)	Similar gene expression dynamics are observed when pseudotemporal gene expression is reconstructed for neuronal lineages in the hindbrain (E) and forebrain (F).

We propose to replace Figure S5 with this updated version.
